# Identification and in Silico Characterization of GT Factors Involved in Phytohormone and Abiotic Stresses Responses in *Brachypodium distachyon*

**DOI:** 10.3390/ijms20174115

**Published:** 2019-08-23

**Authors:** Feng Wen, Liangwei Xu, Yuebin Xie, Liang Liao, Tongjian Li, Mingliang Jia, Xinsheng Liu, Xiaozhu Wu

**Affiliations:** School of Pharmacy and Life Science, Jiujiang University, Jiujiang 332000, China

**Keywords:** GT factors, phytohormones, abiotic stresses, expression pattern, *Brachypodium distachyon*

## Abstract

GT factors play critical roles in plant growth and development and in response to various environmental stimuli. Considering the new functions of GT factors on the regulation of plant stress tolerance and seeing as few studies on *Brachypodium distachyon* were available, we identified GT genes in *B. distachyon*, and the gene characterizations and phylogenies were systematically analyzed. Thirty-one members of *BdGT* genes were distributed on all five chromosomes with different densities. All the BdGTs could be divided into five subfamilies, including GT-1, GT-2, GTγ, SH4, and SIP1, based upon their sequence homology. BdGTs exhibited considerably divergent structures among each subfamily according to gene structure and conserved functional domain analysis, but the members within the same subfamily were relatively structure-conserved. Synteny results indicated that a large number of syntenic relationship events existed between rice and *B. distachyon*. Expression profiles indicated that the expression levels of most of *BdGT* genes were changed under abiotic stresses and hormone treatments. Moreover, the co-expression network exhibited a complex regulatory network between *BdGTs* and *BdWRKYs* as well as that between *BdGTs* and *BdMAPK* cascade gene. Results showed that GT factors might play multiple functions in responding to multiple environmental stresses in *B. distachyon* and participate in both the positive and negative regulation of WRKY- or MAPK-mediated stress response processes. The genome-wide analysis of BdGTs and the co-regulation network under multiple stresses provide valuable information for the further investigation of the functions of BdGTs in response to environment stresses.

## 1. Introduction

As plants are anchored to the soil through their root systems, they have had to develop a series of adaptive mechanisms to help them avoid environmental stress. Stimuli-induced gene expression is a very efficient method to help plants respond to the challenges of the external environment. Transcription factors (TFs), which are ubiquitous in plants, are important factors in regulating gene expression by binding to plant-specific cis-acting elements in the promoter region [1]. They play crucial roles in influencing or controlling many important biological processes, including germination, growth, and signaling transduction and respond to environmental stresses [2]. There are more than 60 TF families in plants, for example, tomato genome encodes at least 998 TFs of 62 different families [3,4,5]. The physiological function of most TF families are being progressively defined, however, the researches on the GT factor family are limited, to date, despite a recent shift in attention [6]. GT factors were first discovered as light response related proteins that bind specifically to the GT element in the promoter of light-induced genes (e.g., *rbcS-3A*) [7]. The core sequence of the GT element, 5’-G-Pu-(T/A)-A-A-(T/A)-3’, was sufficient for light induction and provided the factor’s name [6]. Nevertheless, further studies in rice and *Arabidopsis* showed that some GT factors are not only involved in light-responsiveness at the transcriptional level, but also in abiotic stress responses [8,9].

The GT factor family was predominantly found in plants. As a class of light regulators, GT factors are encoded by a large gene family in various plant species, which occur only in plant. The *GT* genes have been systematically studied in model plants such as *Arabidopsis*, tomato, soybean, chrysanthemum and rice. For instance, there are 30 members in *Arabidopsis*, 36 in tomato, 63 in soybean, 20 in chrysanthemum, and 41 in rice [5,10,11,12,13]. Generally, these family members contain a conserved DNA-binding domain, which has three tandem helices (helix-loop-helix-loop-helix) that combine specifically with the GT elements, a light-responsive DNA element [14]. The amino acid content analyses showed that the DNA-binding domain of GT factors were rich in basic and acidic amino acids, as well as proline and glutamine residues [15,16]. This domain is not a completely new domain because it was similar to the individual repeats of the MYB family from which the trihelix may have been developed [17,18]. According to the changes in their alpha helix domain, they were previously divided into five subgroups, respectively referring as SH4, GT-1, GTγ, SIP1, and GT-2, with the name of each clade based on the first member identified [5]. For example, pea GT-1 factor was the first member identified, which specifically recognized and bound to the GT-elements of light-induced gene *rbcS-3A*’s promoter [7]. Later, the homologous *GT-1* genes were cloned in tobacco, *Arabidopsis*, and rice [19,20,21]. GT-1 clade proteins had one GT domain, while GT-2 types contained two DNA TF domains [17,22]. Strikingly, the GT domains also carried a conserved tryptophan closely upstream or within each amphipathic α-helices. A fourth amphipathic a-helix, with the general sequence (F/Y)-(F/Y)-X-X-(L/I/M)-X-X-(L/I/M) was exited in the GT domains, except SH4 clade but they carry an extended [6]..

Although earlier studies identified that this family was confined to a class of light regulators, recent studies in rice and *Arabidopsis* showed that the GT factors also played important roles in different processes of growth and development involving leaves, flowers, stomata, and seeds. For example, a GT-2 factor, PETAL LOSS (PTL), which was involved in pleiotropic phenotypes including dwarfism, curly leaves, and male sterility, was the first GT factor identified as being associated with floral organ morphogenesis [23]. GRY79, a putative metallo-β-lactamase-trihelix chimera was involved in chloroplast development at the early seedling stage of rice. A loss-of-function *gtl1* mutants were revealed to have larger trichomes and fewer stomata, and further study showed GTL1 was involved in response to abiotic stresses, since it could regulate water use efficiency and drought tolerance by modulating stomatal density [24]. Later, a large number of abiotic stresses-related GT factors was isolated. Four members of OsGTγ subfamily, OsGTγ-1, OsGTγ-2, OsGTγ-3, and OsGTγ-4, were found to be related to cold, drought, and salt stress response [9]. Recently, SIP1-1, a GT family member, played a role in ABA synthesis and signaling, and salt and osmotic stress response in *Brassica napus* [25]. A GT-1 subfamily member, ShCIGT, was proved to mediate cold and drought tolerance by interacting with SnRK1 in tomato. Moreover, many studies indicated GT factors not only participated in response to abiotic stresses, but also played roles in disease resistance [8]. There was evidence that transcript abundance of a rice *GT-1-like* gene, *rml1*, could be rapidly up-regulated in seedlings following infection with the rice blast fungus [26]. *Arabidopsis* ASR3 was rapidly phosphorylated upon MAMP treatment down-stream of MPK4, and acted as a transcriptional repressor to negatively regulate plant innate immunity [27]. GTL1 played a critical role in the MPK4 pathway and acted as a positive regulator of bacterial-triggered immunity and SA homeostasis [28].

Recently, a series of phosphorylation sites were identified in BdGT peptide sequences by large-scale phosphoproteome analysis, suggesting that BdGT factors might be affected through phosphorylation by PKs, which in turn regulated transcriptional activity [29]. It was reported that *BdTHX1*, a member of *BdGT* genes, which was highly co-expressed with the *BdCSLF6*, was speculated to be involved in the regulation of mixed-linkage glucan biosynthesis [30]. Considering the new functions of GT factors on promising candidates for regulation of plant stress tolerance and seeing as few studies on *Brachypodium distachyon* were available, it was of interest for us to isolate GT factors from *B. distachyon*. In this study, a total 31 GT factor family genes were identified from Bd21 genome, and the gene characterizations and phylogenies were systematically analyzed. Furthermore, an expression heatmap of *BdGTs* in response to different hormones and abiotic stresses was also exhibited, a predicted co-expression network was also discussed. The identification and systematical study for GT factors from *B. distachyon* will provide fundamental information for exploring the functions of GT factors in stresses resistance and phytohormone regulation during *B. distachyon*’s adaptation to challenges in the external environment.

## 2. Results

### 2.1. Isolation and Genomic Distribution

A total of 31 *GT* genes have been identified in *B. distachyon* Bd21genome database, and the information for these genes, such as gene names, Locus IDs, gene locations, peptide lengths, and parameters for the deduced polypeptides, are listed (Table 1 and Table S1). The 31 *GT* genes were renamed from *BdGT1* to *BdGT31* according to their order on the chromosomes from chromosomes 1 to 5, respectively, which were divided into five subfamilies, designated as clade GT-1, GT-2, SH4, SIP1, and GTγ. The shortest sequence had 198 amino acid residues, while the longest one had 1391 amino acid residues. The estimated protein molecular weights varied from 21.8 to 152.3 kDa, and the predicted isoelectric points fell in a range of 4.47 to 11.07.

**Figure 1:**
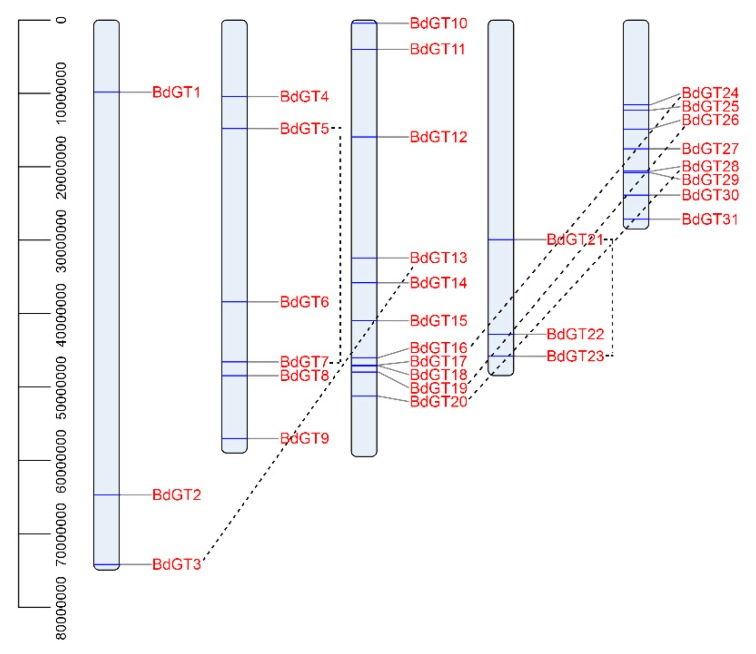
Chromosome distribution of *GT* genes in *B. distachyon*. The chromosome numbers are indicated at the top of each chromosome image. Gene duplication analysis of *BdGTs* was also presented with a dash line.

All of the *GT* genes were distributed on all five chromosomes throughout the *B. distachyon* genome with different densities, the number of *BdGT* genes per chromosome varied from 3 to 11. A maximum number of eleven genes were present on chromosome 3, representing 35.5% of the total *GT* genes, followed by eight on chromosomes 5, six on chromosomes 2, and three each on chromosomes 1 and 4 (Table 1 and Figure 1). As shown in Figure 1, six duplication events were found.

### 2.2. Structural Divergence Among the BdGTs

**Figure 2:**
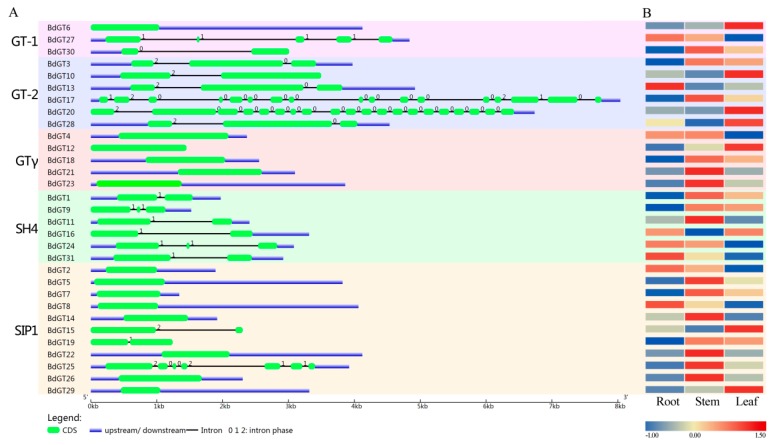
Gene structure and tissue-specific expression heatmap of *BdGTs*. (**A**) Exon-intron organization of corresponding *BdGT* gene. The exons and introns are represented by boxes and lines, respectively. (**B**) Analysis of the *GT* genes in different tissues of *B. distachyon*. Heatmap representation and hierarchical clustering of SIP1, SH4, GTγ, GT-1, and GT-2 genes in root, stem, and leaf.

Gene structure contains the information of possible genetic evolutionary events during gene family’s expanding. In this study, the exon and intron distribution of each individual *BdGT* genes was determined by alignment of the full-length cDNA sequences with the corresponding genomic DNA sequences. As shown in Figure 2, the *GT* genes had considerably divergent gene structures, and the number of *BdGT* exons was discontinuously distributed from 1 through 20. Arranging the gene structure with the subfamily distribution, we found that most of the *BdGT* exon-intron scatter was related to its classification. Closely related genes usually exhibited similar gene structures. For example, the *BdGT* genome sequences in the GTγ subfamily and most STP1 members had no introns and only one exon, and the members of SH4 subfamily contained one or two introns. Therefore, these results indicated that although the *BdGTs* exhibited considerably divergent structures among each subfamily, the gene structure within the same *BdGTs* subfamilies were still relatively conserved. This suggested that the evolution of these gene subfamilies was relatively conservative. In contrast, the structures of the various genes in the GT-1 and GT-2 subfamilies were relatively different, implying that the expansion of GT-1 and GT-2 subfamilies might be divergent during the process of evolution. Extensive studies have proven that the function of the gene was often correlated with its tissue-specific expression patterns. For example, the exocarp tissue of grape, which is involved in pathogen defense and pigment production, showed high mRNA abundance for genes involved with flavonoid biosynthesis, pathogen resistance, and cell wall modification [31]. *AtWRKY12* which has expression in pith and cortex cells of stem and hypocotyls, played a critical role in pith secondary wall formation [32]. To investigate the functional divergence of GT factors in *B. distachyon* growth and development, we detected the expression patterns of *BdGT* genes in root, stem, and leaf by qRT-PCR experiment, followed by a normal standardization method according to a previous report [33]. The results showed high alterations in the expression pattern among different BdGT subfamilies. For example, most of GTγ and SIP1 subfamilies members were detected at high abundance in stem tissues, while most of gene members in GT-2 subfamily were highly expressed in leaves (>2-fold compared with other tissues). For example, the expression level of *BdGT10*, *BdGT20*, and *BdGT28* were 3.16-fold, 199.9-fold, and 2.4-fold higher in leaves than that in stems. Almost all of *BdGT* genes except the SH4 subfamily were detected at a relatively low expression level (<0.5-fold) in root tissues compared with that in stems, for instance, the expression level of *BdGT20* (a member of GT-2 subfamily) was 124.5-fold lower in roots than that in stems. Most duplicated gene pairs (such as *BdGT5* and *BdGT7*, *BdGT19* and *BdGT26*, *BdGT20* and *BdGT28*, and *BdGT21* and *BdGT23*) presented largely similar expression patterns, implying their functional redundancy (Figure 2). Notably, the expression level of *BdGT3* was very low in root, but its paralogous gene, *BdGT3*, was extremely highly expressed in the same tissue, suggesting these two GTs might perform a different function in root growth and development.

**Figure 3:**
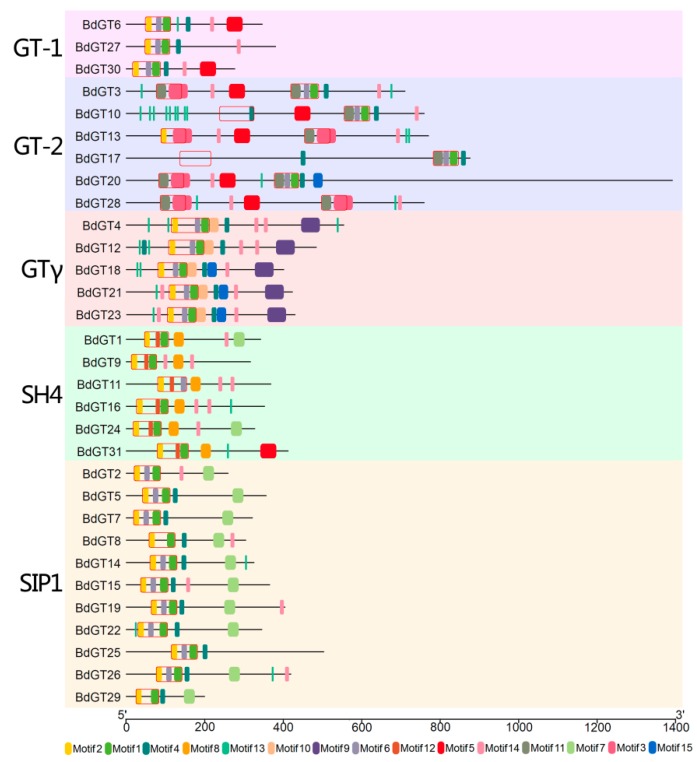
Protein structures of BdGTs in *B. distachyon*. Each different motif is represented by a specific color, and the conserved GT domain is represented by the red box.

To determine the functions of the GT factors, the BdGT motif composition was analyzed by amino acid sequence in the MEME program, accompanied by a NCBI-CDD annotation. Fifteen conserved motifs within the *B. distachyon* GT factors were identified. The GT factors of *B. distachyon* can be clearly classified into five subfamilies based on the composition of motifs (Figure 3). Generally, all GT factor family genes contained various trihelix DNA binding domains (WWW, WWF, and WWI) located at the N-terminus of the amino acid sequence, while there were two DNA binding domains in the GT-2 clade (Figure 3 and Figures S1 and S2). For example, the members in GT-1 and SH4 subfamilies contained a WWW type trihelix DNA binding domain, which meant conserved tryptophans (W) were located at the front of three individual amphipathic α-helix. The third α-helix contained conserved phenylalanine (F) in GT-2 (N-terminal trihelix domain) and GTγ subfamilies, while isoleucine (I) or valine (V) was existed in SIP1 subfamily members. The trihelix DNA binding domain was also annotated by NCBI-CDD database as the GT domain, which was composed of motif 2, motif 1, and motif 6, or other variations. Furthermore, the fourth amphipathic α-helix, with the general sequence (F/Y)-(F/Y)-X-X-(L/I/M)-X-X-(L/I/M), was shown as motif 4 (Figure 3 and Figures S1 and S2). Besides, it was not present in the SH4 clade, but they carried an extended third trihelix (motif 8). Consistent with the gene structure analysis results, the gene motifs and distribution patterns were closely related to their subfamilies. For instance, GT-2 contained two GT domains, the GTγ subfamily contained motif 9 and motif 10, and all SIP1 members contained motif 7.

### 2.3. Phylogenetics and Synteny Analysis of BdGTs

**Figure 4:**
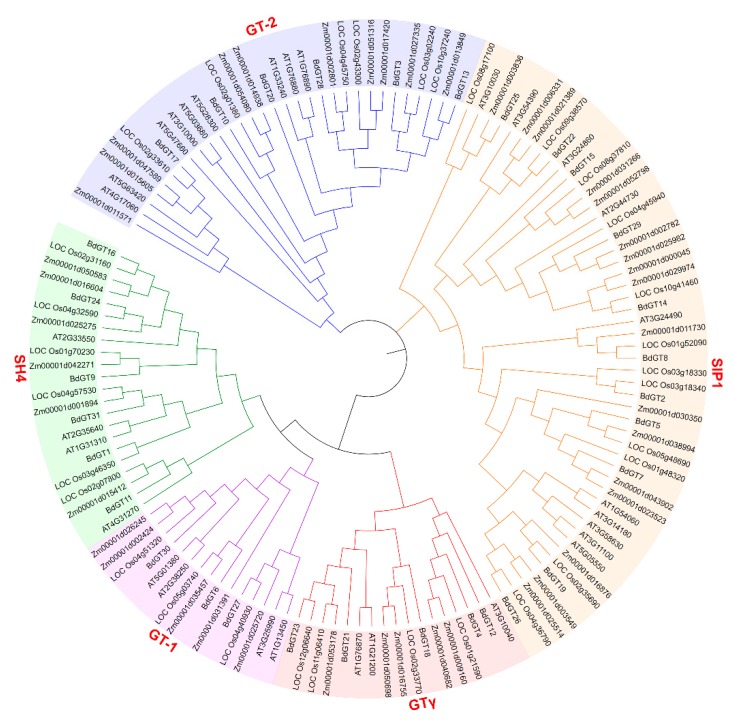
Phylogenetic analysis of *Arabidopsis*, maize, rice, and *B. distachyon* GT factors.

**Figure 5:**
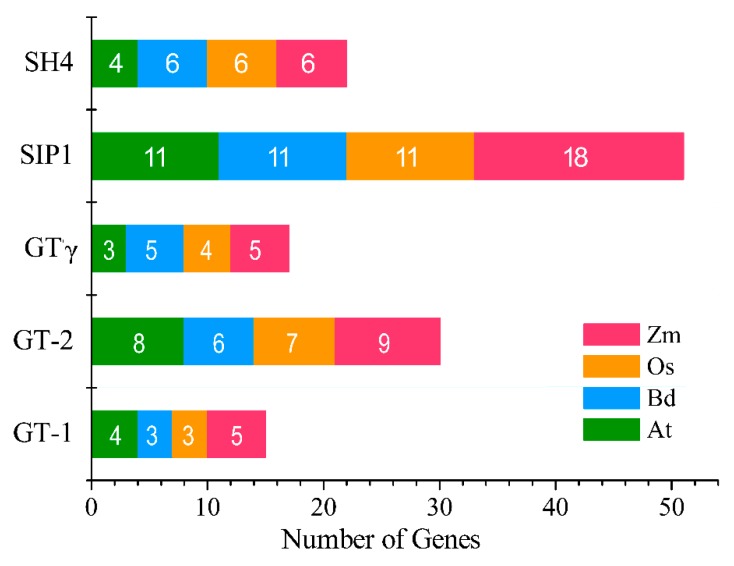
The distribution of *GT* genes in *Arabidopsis*, maize, rice and *B. distachyon*. The total number of SH4, SIP1, GTγ, GT-2, and GT-1 subfamily genes found in each genome is indicated in the bar.

To explore the function and phylogenetic relationship of BdGTs between dicots and monocots, the dicot model plant *Arabidopsis*, the monocot model plant rice, maize, and *B. distachyon* GT factors full-length amino acid sequences were used to construct an unrooted phylogenetic tree. The neighboring-joining phylogenetic distribution suggested that the organization of these BdGT proteins was very similar to each other in subfamily GT-1, GT-2, GTγ, SH4, and SIP1, implying that BdGTs within these classes were derived from a common ancestor (Figure 4). In general, all GTs and their subfamilies were present in monocots and dicots, indicating that the appearance of most of the GT factors in plants predates the monocot-dicot divergence and GT factors were conserved during evolution. The SIP1 clade was the largest subfamily in *B. distachyon*, containing 11 GTs, whereas the GT-1 clade was the smallest, consisting of three members, indicating that GTs were distributed unevenly in the different clades (Figure 5). The distribution of genes in each subfamily from *B. distachyon* was consistent with which from rice and maize, while it was very different with *Arabidopsis*. For instance, the GTγ subfamily contained the fewest member of GTs in *Arabidopsis*, and an obviously larger proportion of GT-2 members were also observed in *Arabidopsis* than in the monocot species, such as rice, maize and *B. distachyon* (Figure 5). Moreover, five AtGTs in the SIP1 subfamily was clustered into one sub-branch, which exhibited the difference between monocots and dicots, suggesting that these members might arise after dicot-monocot divergence. Since genes in the joint phylogenetic tree were fell as related sister pairs, twelve related sister pairs were observed between the *B. distachyon* and rice GT families, while two were found between the *B. distachyon* and maize GT families [34,35]. These results imply that *B. distachyon* may have a closer evolution relation with rice than maize.

**Figure 6:**
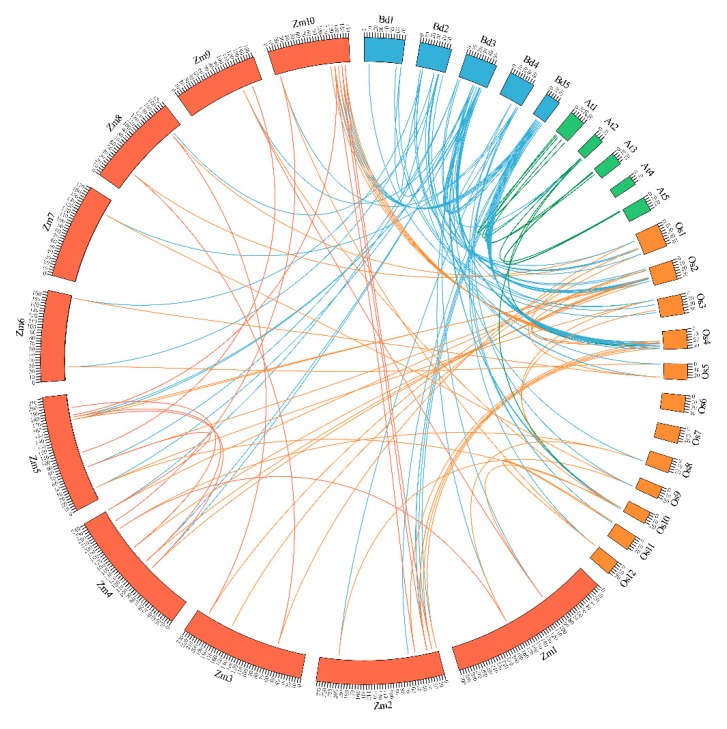
The syntenic analysis of *GT* genes among *Arabidopsis*, maize, rice, and *B. distachyon*. Schematic representation of 135 couples of duplicated genes was displayed on 5 *Arabidopsis* chromosomes, 10 maize chromosomes, 12 rice chromosomes, and 5 *B. distachyon* chromosomes by connecting lines using the CIRCOS software. The size of chromosomes was consistent with the actual pseudo-chromosome size. Positions are in Mb.

To further understand the gene duplication mechanisms of the *B. distachyon* GT factor family, a comparative syntenic analysis of *B. distachyon* associated with three representative species, including one dicot (*Arabidopsis*) and two monocots (rice and maize) were carried out, and the MCScanX results were represented by circos software (Figure 6). A total of 30 *B. distachyon GT* genes showed a syntenic relationship with those in rice, followed by maize (18), and *Arabidopsis* (2), indicating that in comparison with monocotyledonous plants, *B. distachyon GT* genes show a high evolution divergence with dicotyledonous plants. These results were also consistent with the result of phylogenetic analysis, suggesting that the evolution relationship between rice and *B. distachyon* was closer than that between *B. distachyon* and maize. Furthermore, some *BdGTs* were found to be associated with at least four syntenic gene pairs, such as *BdGT5*, *BdGT14*, *BdGT16*, *BdGT23*, *BdGT26,* and *BdGT29*. These genes might have played a crucial role in the *GT* gene family during evolution.

### 2.4. Expression Profile and Co-Regulatory Network of BdGT Genes in Response to Hormone and Abiotic Stresses

**Figure 7:**
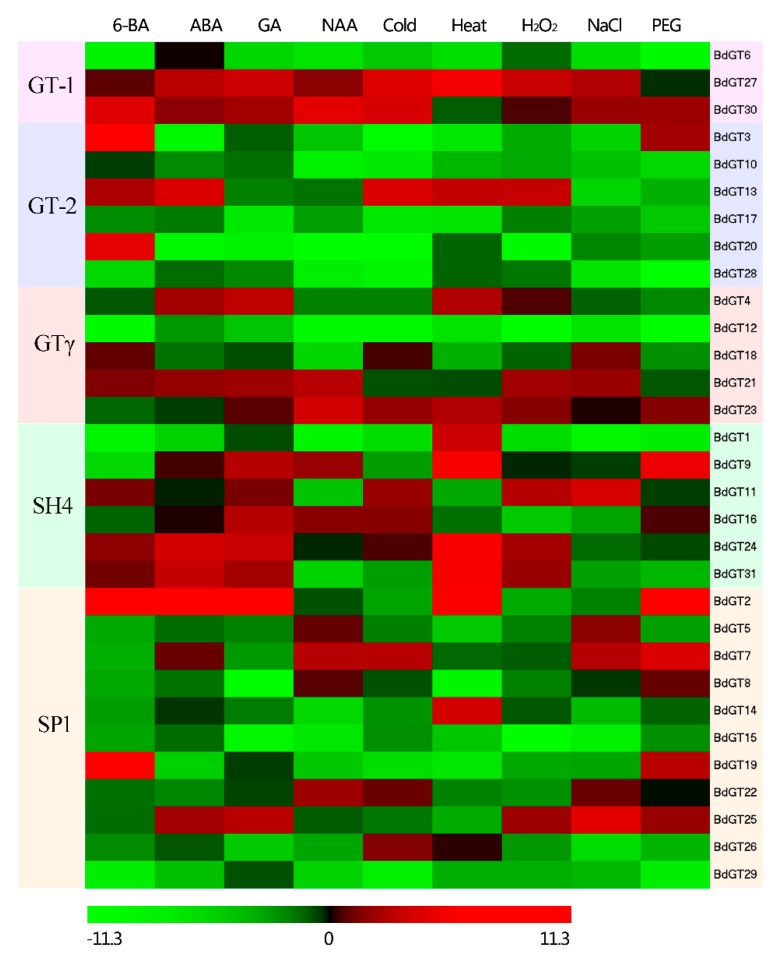
Expression patterns of *GT* genes in *B. distachyon* in response to phytohormone and abiotic stresses. Levels of up-expression (red) or down-expression (green) are shown on a log2 scale from the highest to the lowest expression of each *BdGT* gene.

Although the GT factors was firstly considered as light regulators involved in plant growth and development in plants, extensive research has revealed that these family genes also played critical roles under the changeable environmental conditions. To understand the expression profiles of *BdGT* genes in response to different environmental stimuli, the expression patterns of 31 *B. distachyon GT* genes were studied in response to various hormones (100 μM abscisic acid (ABA), 20 μM 6-benzylaminopurine (6-BA), 5 μM 1-naphthylacetic acid (NAA), and 10 μM gibberellin A3 (GA3)), and abiotic stresses (20% polyethylene glycol (PEG), 200 mM NaCl and 10 mM H_2_O_2_, 4 °C (cold), and 45 °C (heat)) treatments using qRT-PCR experiment. The heatmap represents the transcript expression fold change under different abiotic stresses and hormone treatments according to the qRT-PCR results (Figure 7). As shown in heatmap representation, differential expression levels of *BdGT* genes were exhibited under various treatment according to the cluster analysis results. Meanwhile, the expression profiling of the five subgroups of *BdGT* genes also showed great divergence. Under the four hormone treatments, less genes were induced by NAA treatment compared to the other hormone treatment. The results indicate that 20 (64.5%) genes were induced at least one phytohormone treatment, and three *BdGTs* (*BdGT21*, *BdGT27*, and *BdGT30*) were induced by these four type phytohormones. It seems that the GT-1 and SH4 clade genes are more sensitive to hormone treatments, while the majorities of GT-2 and SP1 clade genes were down-regulated after hormone treatments. Although the expression level of a large number of *BdGT* genes was decreased after hormone treatments, some *BdGTs* showed an extremely high expression level in response to hormones. For example, *BdGT2*, *BdGT3*, and BdGT19 were significantly up-regulated in the seedlings by treatment with 6-BA with *p*-value < 0.05, suggesting these BdGTs might play critical roles in the cytokinin-induced signaling pathway (Figure 7 and Figure S4). Under five abiotic stresses (cold, heat, H_2_O_2_, NaCl and PEG), most of *BdGT* genes were down-regulated by these abiotic stresses treatment. By contrast, most of SH4 clade genes were significantly induced in response to heat treatment and slightly induced by cold and H_2_O_2_ treatment but showed repression after PEG and NaCl treatments. In GT-2 clade, all the six *BdGT* genes (except *BdGT3* in response to PEG) were significantly down-regulated under PEG and NaCl treatments, as well as GA and NAA treatment. Additionally, *BdGT27* were significantly induced by heat and cold stress (*p*-value < 0.01), implying it might play critical roles in the external temperature perception signaling pathway.

**Figure 8:**
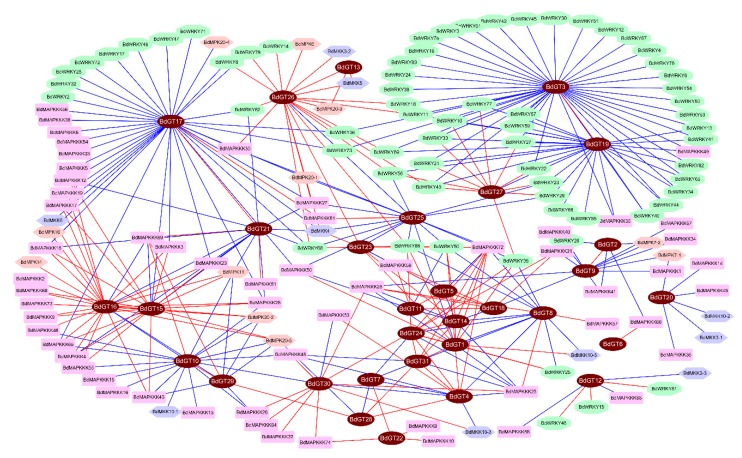
Regulatory networks of *GT* genes. Co-expression regulatory network among *BdGT*, *BdWRKY*, and *BdMAPK* cascade genes upon different stress treatments based on the Pearson correlation coefficients of the relative expression of genes.

To further understand the connection between *BdGTs* and *BdWRKYs* as well as that between *BdGTs* and the *BdMAPK* cascade gene, we constructed the co-expression regulatory network among these genes upon different stress treatments based on the Pearson correlation coefficients of the relative expression of genes. Results show that a large number of *BdGT* genes exhibited co-expression correlation with *BdWRKYs* and *BdMAPK* cascade gene, suggesting BdGTs might be involved in BdWRKY and BdMAPK cascade induced signal transduction pathway. As shown in Figure 8, *BdGT3* and *BdGT17*, as well as *BdGT19*, exhibited a strong negative co-expression correlation with a set of *BdWRKYs*, indicating that these BdGTs might be transcriptional repressors to negatively regulate the BdWRKY involved stress response in *B. distachyon*. *BdGT15* and *BdGT16* showed positive co-expression levels with a large number of *BdMAPKKKs*. Moreover, *BdGT10* and *BdGT29* exhibited negative expression correlation with some *MAPK* cascade genes, implying these BdGT might mediated the inhibition of MAPK activation. A set of *BdGTs* (*BdGT1*, *BdGT5*, *BdGT11*, *BdGT14*, *BdGT24*, and *BdGT31*) showed a similar regulatory pattern, implying they might be a functional redundancy. Interestingly, *BdGT26* showed a strong positive co-expression correlation with a set of *BdWRKYs* and *BdMAPK* cascade genes, suggesting that BdGT26 might play a key role in regulating the crosstalk between BdWRKYs and the BdMAPK cascade. The co-expression regulatory network could reveal a deductive signaling pathway of stress response in *B. distachyon*, which showed that the BdGTs might be involved in WRKYs- or MAPK cascade-induced stress response processes.

## 3. Discussion

Environmental stresses affect plant growth, development, and survival and limits the agricultural crop productivity [25,36]. To cope with various abiotic stresses, plants have evolved various adaptive mechanisms to respond stressful conditions by activating stress-responsive pathways [37]. Transcriptional regulation of gene expression plays a major role. During the evolution, the expansion of transcription factor gene families via genome duplication events support plants to adapt better to diversified environmental stresses. Early studies identified the GT factor family as a class of light regulators hence their functions in the regulation of light-responsive genes [6,20,38]. Nevertheless, recent studies exhibit strong evidences to prove that the GT factor family also played important roles in growth and development, as well as in response to environmental stimuli [6,13,23,27,39,40,41]. In this study, a total 31 *GT* factor family genes were identified in the *B. distachyon* genome, and they contained a high number of gene copies (Table 1). The *B. distachyon GT* genes were distributed on chromosomes with different densities (Figure 1). Only six duplication events were found, and tandem duplications were found among three *BdGT* gene pairs, such as *BdGT16* and *BdGT24*, *BdGT19* and *BdGT26*, and *BdGT20* and *BdGT28*. The results suggest *BdGT* genes were less conserved, implying most genes might not originate from the same ancestor, which was consist with the report in rice [12]. Gene synteny analysis showed that almost all *B. distachyon GT* genes (except *BdGT12*) showed a syntenic relationship with those in rice, maize, and *Arabidopsis*, implying this family genes had a high degree of retention following whole-genome duplication (Figure 6). In addition, a set of *BdGTs* were found to be associated with multiple syntenic gene pairs, suggesting these genes might play a crucial role in the *GT* gene family during evolution, and the functional requirement played important roles in both plants’ developmental processes and defenses during gene family expansion [42,43].

Although the GT factor family genes were not conservative during evolution, the most members of *B. distachyon* GT factor family gene contained a conserved DNA-binding domain, which was similar to the individual repeats of the MYB family, containing a typical three tandem helices (helix-loop-helix-loop-helix) structure [17,44]. Moreover, duplicated genes occurred in GT-2, SH4, and SIP1 subfamilies and exhibited a similar gene and protein structure. In fact, the gene structure and conservative domain analyses showed that *BdGT* genes within the same subfamilies (such as GTγ, SH4, and SIP1) were still relatively conserved (Figure 2 and Figure 3). The MEME analysis showed that the conservative GT domain distribution of each BdGT was related to its classification (Figure 3). Therefore, these conserved DNA binding domains might perform their physiological function in a group-specific manner. The gene structures among the various groups differed greatly from the conserved functional domains. For this reason, they may have different downstream regulatory genes and participate in different signaling pathways. For instance, according to our conservative domain analyses, GT-2 contained two GT domains, which were different from those in the GT-1 subfamily, implying a functional divergence might exist between GT-1 and GT-2 subfamilies [45]. Hence, the GT clade in *B. distachyon* was divided into the GT-1 and GT-2 subfamilies, which was consist with previous studies (Figure 4) [9,13]. Gene synteny analysis showed that all members in GT-2 and SH4 subfamilies exhibited a syntenic relationship with those in rice, but less close with those in maize, implying that these two subfamilies were expanded after BEP and PACCAD branches divergence. These results were also consistent with the genetic relationships between Pooideae and Oryzoideae that was closer than that between Pooideae and Panicoideae [46].

Firstly, the GT factors were considered as light regulators involved in plant growth and development. Recent studies reported that some GT factors were involved in the basic resistance to abiotic stresses [8,24,47]. Moreover, the phytohormones were considered as major modulators in plant adaptation and responses to various environmental stresses [48]. To further evaluate the possible functional divergence of *BdGT* genes during abiotic stress and understand the relationship between phytohormone homeostasis and abiotic stresses, the expression patterns of 31 selected *B. distachyon GT* genes were studied in response to various abiotic stresses (including cold, heat, H_2_O_2_, NaCl, and PEG) and four hormone treatments (including 6-BA, ABA, GA, and NAA) using a qRT-PCR experiment (Figure 7). The heatmap representation showed that the expression levels of most of *BdGT* genes were changed under abiotic stresses and hormone treatments (a fold-change greater than 2 or less than 0.5 with *p*-value < 0.05), suggesting that *BdGT* genes might play multiple function in response to various environmental stimuli. For instance, 20 *BdGT* genes were induced at least one phytohormone treatment, while 23 *BdGT* genes were down-regulated at least one phytohormone treatment. This was consistent with previous studies, which showed that some of the gene members in the GT-1, GT-2, and GTγ subfamilies were involved in the abiotic-stress response, acting as positive or negative factors to regulate the adapt ability of plants to stresses and hormones [8,9,47,49]. Meanwhile, the expression pattern of the five subfamilies of *BdGT* genes showed great divergence, which was consistent with the previous opinions that *BdGT* genes might perform their physiological function in group-specific manner. MAPK cascade was a pivotal phosphorylation pathway to transmit external or internal signals to downstream effectors [50]. Numerous reports pointed that WRKY transcription factors were one of the largest families of transcriptional regulators, which could form integral parts of signaling networks to modulate plant to adapt environmental stimuli [51,52,53]. To explore the potential regulatory networks between BdGTs and BdWRKYs as well as that between BdGTs and BdMAPK cascade gene, we constructed the co-expression regulatory network among these genes upon gene expression pattern. Results showed that a large number of *BdGT* genes exhibited co-expression correlation with *BdWRKYs* and *BdMAPK* cascade gene, suggesting these genes might be involved in same regulatory pathway.

## 4. Materials and Methods

### 4.1. Identification and Characterization Analysis of B. Distachyon GT Genes

All the *B. distachyon* genome sequence data were downloaded from Phytozome V12 (https://phytozome.jgi.doe.gov) [54]. The protein sequences of A. thaliana GTs were obtained from TAIR database (http://www.arabidopsis.org). To identify the GT factor family members in *B. distachyon*, the *Arabidopsis* and rice *GT* gene sequences were used as the query to perform a BLASTP search against 52972 sequences of the protein database of *B. distachyon*, with a cutoff *e*-value ≤ *e*^−10^. The SMART (http://smart.embl-heidelberg.de/) and InterPro (http://www.ebi.ac.uk/interpro/) online tools were used to analyze these potential sequences to validate the BLAST search [55]. The theoretical isoelectric point and molecular weight were estimated by pI/Mw tool (http://web.expasy.org/compute_pi), while WoLF PSORT was used to predict the subcellular localization of BdGT proteins (https://www.genscript.com/wolf-psort.html). All conserved domains were investigated by multiple alignment analyses using ClustalW, and the phylogenetic analysis for BdGTs was performed by using MEGA-X program by the neighbor-joining method, with bootstrap value from 1000 replicates indicated at each node with the following parameters: p-distance and pairwise deletion. 

### 4.2. Gene Structure and Chromosomal Locations

The *BdGT* gene structures were displayed by comparing the coding sequences and corresponding genomic DNA sequences with the Gene Structure Display Server tools (http://gsds.cbi.pku.edu.cn/) [56]. The chromosomal locations of the *BdGT* genes were determined using the *B. distachyon* genome browser and mapped by using a bioinformatics toolkit TBtools [57]. And then, gene duplication events were determined according to previous reports: (1) The alignable region between two genes was ≥80% of the longer gene; (2) the similarity between the two aligned genes was ≥ 70%; and (3) tightly linked genes on the same chromosome were considered as tandem duplication [58,59,60]. The Multiple Collinearity Scan toolkit (MCScanX) was used for the synteny analysis, and the result is graphic by Circos software (http://circos.ca/) [61,62].

### 4.3. Protein Structure and Conserved Motif Analysis

The MEME program (http://meme-suite.org/) was used to identify the conserved motifs of the *B. distachyon* GT factors with the following parameters: Any number of repetitions of a single motif, the maximum numbers of different motifs up to 15 motifs, the minimum motif width with six amino acids, the maximum motif width of a motif with 80 amino acids [63]. The details of the sequence logo of motifs were shown in Figure S3. The Batch CD-Search (https://www.ncbi.nlm.nih.gov/cdd) was used to identify the conserved GT domain in *B. distachyon* GT factors [64]. Subsequently, a bioinformatics toolkit TBtools was used to draw the diagram.

### 4.4. Expression Analysis of BdGTs

Seeds of *B. distachyon* Bd21 were germinated on 1/2 Murashige and Skoog medium (MS) solid medium and grew in temperature-controlled (25 °C) growth chambers under a 16-h light/8-h dark cycle. The Bd21 seedlings were used for tissue-specific expression analysis and stress or hormone treatments according to previous work with some modifications [65]. For tissue-specific expression analysis, 2-week-old seedlings were used to collect the roots, stems and leaves. For hormone and abiotic stress treatment, 2-week-old seedlings were treated in MS liquid medium containing 100 μM ABA, 20 μM 6-BA, 5 μM NAA, 10 μM GA3, 20% PEG, 200 mM NaCl, and 10 mM H_2_O_2_ for 3 h, respectively. Cold and heat treatments were achieved by placing 2-week-old seedlings in MS liquid medium at 4 or 45 °C for 3 h, respectively. Total RNA was extracted by the TRIzol method and treated with DNaseI to eliminate any DNA contamination. First-strand cDNA (20 µL) was synthesized according to the instructions for the PrimeScript™ RT Master Mix (Takara Biomedical Technology (Beijing) Co., Beijing, China). Gene specific primers for quantitative real-time PCR are listed in Table S2. The expression of *GT* genes was assessed upon the qPCR result analysis. Each experiment was repeated three biological replications. *BdActin* (Bradi2g24070) gene was the internal reference gene. For tissue-specific analysis, the average of total ΔCT value (ΔCT. average) was subtracted from all other ΔCT values to obtain second normal standardization, according to the previous method, using the formula: u = (ΔCT − ΔCT. average)/σ (in which, u is the value after normal standardization, and σ is the standard deviation) [33]. The *BdGT* gene expression profiles were calculated from the −ΔΔCT value (−ΔΔCT = (CT_control.gene_ − CT_control.actin_) − (CT_treat.gene_ − CT_treat.actin_)), and a heatmap was generated by PermutMatrixEN version 1.9.3 software (https://en.bio-soft.net/chip/PermutMatrix.html). Two tailed Student’s *t*-test (*p* 0.05) was used to determine the significant difference of relative expression of individual *BdGT* genes between control and different treatments (Microsoft Excel 2007). Fold-change greater than 2 with *p*-value of <0.05 was defined as up-regulated gene, while a fold change of 0.5 or less was used to define down-regulated genes when the *p*-value of <0.05 (Table S3). The expression level of *BdGT2*, *BdGT3*, *BdGT19*, *BdGT21*, and *BdGT30* under multiple hormones and abiotic stresses treatment were shown in Figure S4 by using semi quantitative reverse PCR.

### 4.5. Predicted Co-Expression Network

The Pearson correlation coefficients (PCCs) of transcript levels of gene pairs were calculated by Microsoft Excel 2007, based on log2-transformed quantitative Real-Time (qRT)-PCR data. For gene co-regulatory network analysis, the gene pairs, whose PCCs was greater than 0.8, were selected. Based on the PCCs of these gene pairs, the co-expression networks were represented by using Cytoscape [66].

## 5. Conclusions

The identification and systematical study of GT genes in *B. distachyon* can help scientists to better explore the functions of BdGTs in integrating light signaling pathways in *B. distachyon* in adaptation to vagaries of environments. In this study, 31 members of BdGT genes were identified. The gene characterizations and phylogenies have been systematically analyzed. A phylogenetic tree revealed that BdGT family members can be clustered into five subfamilies (GT-1, GT-2, GTγ, SH4, and SIP1), based upon sequence homology. Although the BdGT genes were less conservative between each subfamily, *BdGT* genes within the same subfamilies were still relatively conserved. Synteny results indicated that large number of syntenic relationship events existed between rice and *B. distachyon*, indicating that many consensuses in BdGT protein may have existed before the species divergence between rice and *B. distachyon*. The expression patterns revealed the involvement of BdGT genes in various phytohormones and in response to abiotic stresses. Moreover, the co-expression network implied that there was a complex regulatory network between *BdGTs* and *BdWRKYs* as well as that between *BdGTs* and *BdMAPK* cascade genes, and BdGTs might be both the activator and the repressor involved in WRKY transcription factors or MAPK cascade mediated stress response processes. Our study provided a systematical study of *GT* genes in *B. distachyon* under multiple phytohormones and stresses conditions, which is an important step for the further investigation of the functions of *BdGT* genes across different plant species.

## Figures and Tables

**Table 1:** GT factor family genes in *B. distachyon*.

Gene Name	Gene Locus	Chr	ORF (bp)	Exon No.	PI	MW (kD)	Length (aa)	Subcellular Localization	GT Domain
***BdGT1***	Bradi1g12900	Chr1	1026	2	8.68	37.20	341	nucl	44–146
***BdGT2***	Bradi1g65400	Chr1	780	1	9.65	27.84	259	chlo/mito	19–112
***BdGT3***	Bradi1g77610	Chr1	2130	3	8.75	75.46	709	nucl	76–162, 419–515
***BdGT4***	Bradi2g12030	Chr2	1662	1	6.28	59.29	553	nucl	109–262
***BdGT5***	Bradi2g16780	Chr2	1068	1	9.92	37.91	355	nucl	41–130
***BdGT6***	Bradi2g38230	Chr2	1038	1	6.07	38.46	345	nucl	50–163
***BdGT7***	Bradi2g46320	Chr2	963	1	9.8	35.03	320	nucl	17–106
***BdGT8***	Bradi2g48320	Chr2	912	1	6.57	33.82	303	nucl	57–153
***BdGT9***	Bradi2g59440	Chr2	948	3	5.75	34.50	315	chlo/nucl	12–145
***BdGT10***	Bradi3g00697	Chr3	2277	2	5.88	80.53	758	nucl	238–325, 555–643
***BdGT11***	Bradi3g05530	Chr3	1104	2	4.23	38.37	367	nucl/cyto	80–189
***BdGT12***	Bradi3g17539	Chr3	1452	1	7.62	52.38	483	cyto	102–251
***BdGT13***	Bradi3g30457	Chr3	2310	3	5.72	82.09	769	nucl	87–173, 454–541
***BdGT14***	Bradi3g33630	Chr3	975	1	9.53	34.30	324	nucl/chlo	60–152
***BdGT15***	Bradi3g38682	Chr3	1098	2	6.25	40.60	365	nucl	35–125
***BdGT16***	Bradi3g44370	Chr3	1056	2	5.12	37.76	351	chlo/nucl	26–148
***BdGT17***	Bradi3g45230	Chr3	2628	17	8.9	96.63	875	chlo	116–216, 782–865
***BdGT18***	Bradi3g45300	Chr3	1203	1	5.77	46.18	400	nucl	74–205
***BdGT19***	Bradi3g46210	Chr3	1212	2	6.54	43.22	403	nucl	62–147
***BdGT20***	Bradi3g50213	Chr3	4176	20	4.47	152.26	1391	chlo	82–168, 377–454
***BdGT21***	Bradi4g24750	Chr4	1269	1	7.01	47.83	422	nucl	103–234
***BdGT22***	Bradi4g37730	Chr4	1035	1	7.56	36.98	344	nucl	29–135
***BdGT23***	Bradi4g41830	Chr4	1290	1	5.76	49.11	429	nucl	98–229
***BdGT24***	Bradi5g08600	Chr5	981	3	6.3	35.24	326	nucl/cyto	17–134
***BdGT25***	Bradi5g08980	Chr5	1509	7	7.53	53.76	502	nucl	114–206
***BdGT26***	Bradi5g11070	Chr5	1257	1	6.97	45.34	418	nucl	75–160
***BdGT27***	Bradi5g13900	Chr5	1140	5	5.96	41.40	379	chlo/nucl	48–138
***BdGT28***	Bradi5g17150	Chr5	2277	3	6.11	81.89	758	nucl/pero	86–172, 497–584
***BdGT29***	Bradi5g17281	Chr5	597	1	11.07	21.82	198	chlo/nucl	23–98
***BdGT30***	Bradi5g20847	Chr5	828	2	8.97	32.09	275	nucl	17–107
***BdGT31***	Bradi5g25700	Chr5	1236	2	10.05	44.26	411	chlo/nucl	78–215

## Supplementary Materials

Supplementary materials can be found at https://www.mdpi.com/1422-0067/20/17/4115/s1.

## Abbreviations

TFs	Transcription factors
6-BA	6-benzylaminopurine
ABA	abscisic acid
NAA	1-naphthylacetic acid
GA3	gibberellin A3
PEG	polyethylene glycol
PCCs	Pearson correlation coefficients
