# Supplementary figures and images for: Identification and in Silico Characterization of GT Factors Involved in Phytohormone and Abiotic Stresses Responses in Brachypodium distachyon
Source: Int J Mol Sci. 2019 Aug 23;20(17):4115. doi: 10.3390/ijms20174115 (PMC6747514; doi:10.3390/ijms20174115)

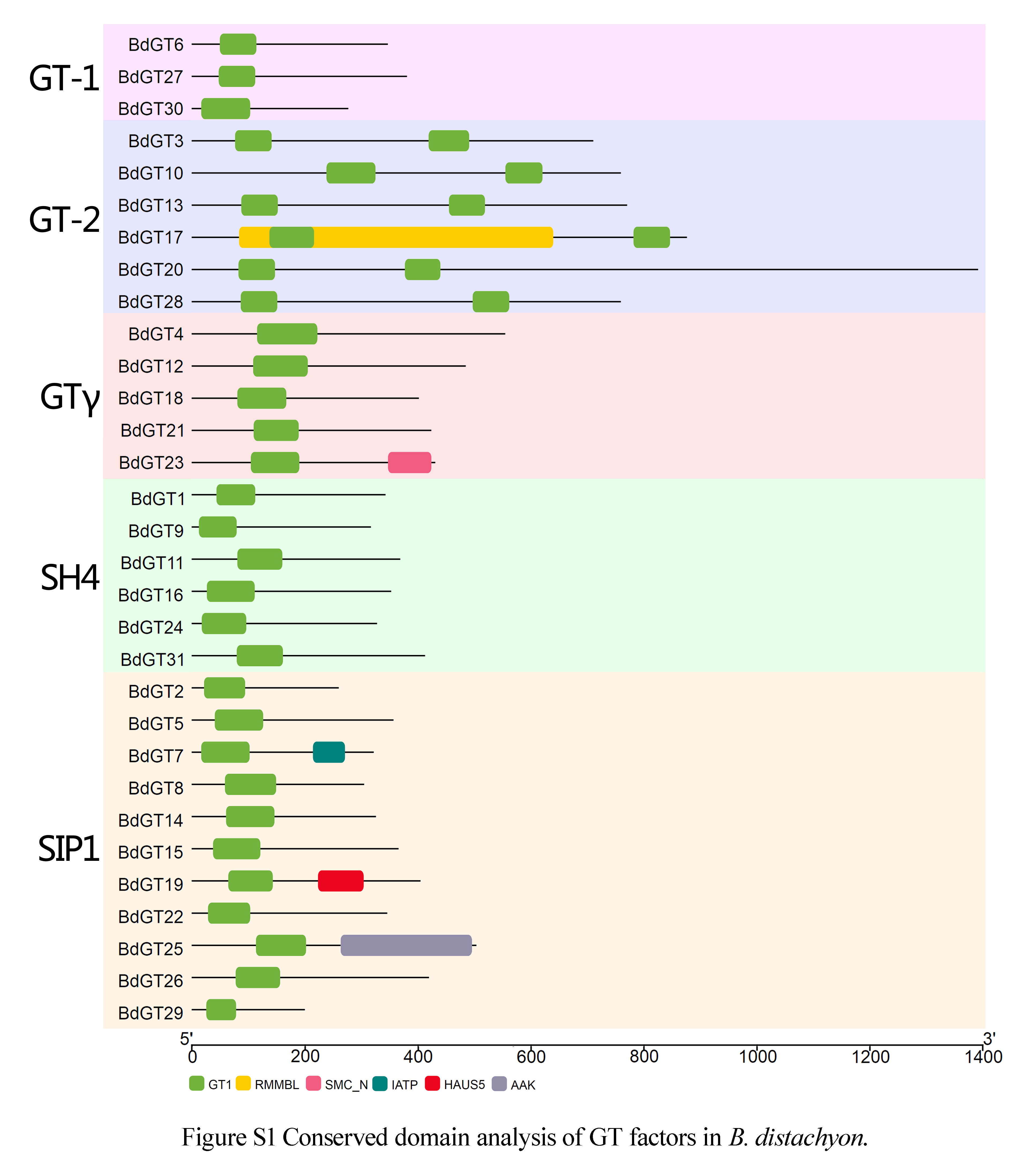

Supplement: Supplementary file 1 [file ijms-20-04115-s001.zip › supplementary file/Figure S1.tif]

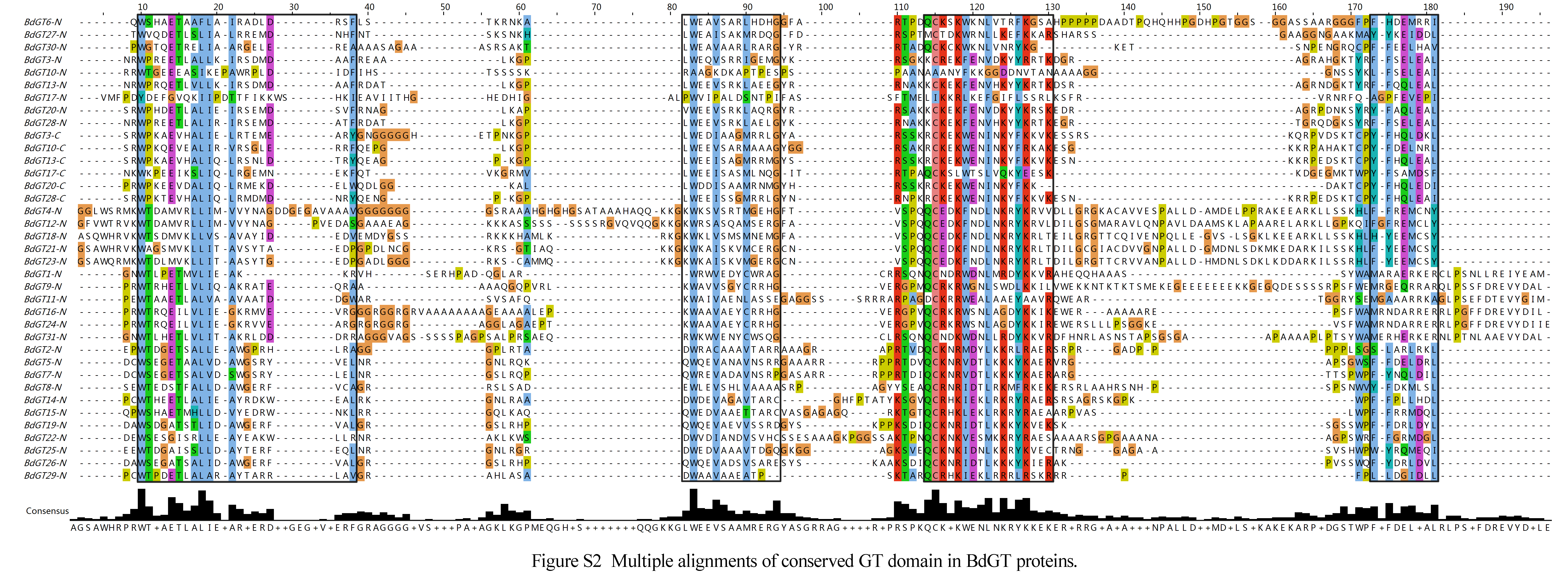

Supplement: Supplementary file 1 [file ijms-20-04115-s001.zip › supplementary file/Figure S2.tif]

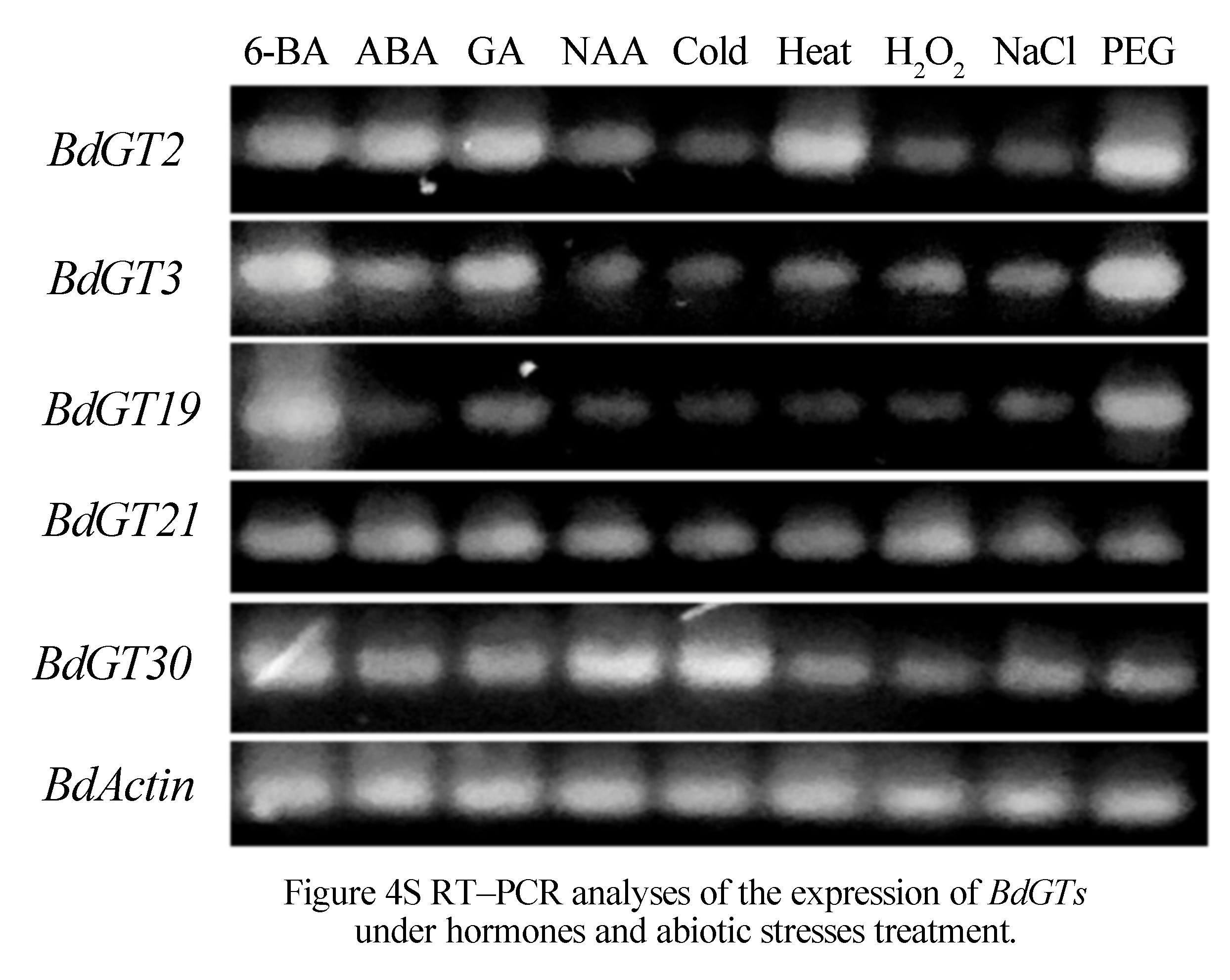

Supplement: Supplementary file 1 [file ijms-20-04115-s001.zip › supplementary file/Figure S4.tif]
