# Supplementary material for: Identification and in Silico Characterization of GT Factors Involved in Phytohormone and Abiotic Stresses Responses in Brachypodium distachyon
Source: Int J Mol Sci. 2019 Aug 23;20(17):4115. doi: 10.3390/ijms20174115 (PMC6747514; doi:10.3390/ijms20174115)
Supplement: Supplementary file 1 [file ijms-20-04115-s001.zip › supplementary file/Table S1.doc]

Table S1 The CDS and protein sequence of BdGTs.

| Gene name | Type | Sequence |
| --- | --- | --- |
| BdGT1 | CDS | ATGGACATGAAGCAGCAGGGATCCGCCGGCGTCATGGAGCGATCAGGCCAGCAAGGAGATCAGGACGCGGGCAGGAACCTGAGCGCGGCTGGCGCCATGACGCTGGCAACAGTGAGGGAGTACCGGAGGGGCAACTGGACGCTGCCGGAGACGATGGTGCTGATCGAGGCGAAGAAGCGTGTGCACTCGGAGCGGCACCCGGCGGACCAGGGCCTGGCCCGGTGGCGCTGGGTGGAGGACTACTGCTGGCGCGCCGGGTGCCGCCGGAGCCAGAACCAGTGCAACGACCGCTGGGACAACCTCATGAGGGACTACAAGAAGGTGCGCGCCCACGAGCAGCAGCACGCCGCCGCGAGCTCCTACTGGGCCATGGCCAGGGCGGAGCGCAAGGAGCGCTGCCTCCCTTCCAACCTCCTGCGCGAGATCTACGAGGCCATGGGCGAGATCGTCGAGAGCCGGCGGAGGTTGAGCTGCTACGGCGGCTCCGGCTCCGGCTCCGGCGTTTTGTTCTTGGGGGCGCCCTTGACTGCCACCAACGTCGGCGCCGACATCCCCGCCATGCAGGCCTCCCCGCTCGCCCAAGTCCCGCCGCCCCGGCCTTTCGAGCAAGGAACGCACTGCAGCGCGTCCGAGTCGCCGGAGAGGAAGAGGCTGCGGCAGTCGCTGGACGGGCAGCCCCGGAGCAGCACGCCGGCAGCGGCATCGGCATCGGCAGGAATACACCATCAAGAAGCCCAGCCCCTGCAGGAAGACGACGAGAGCTCGGGCGAGGACGAAGACGCCCTGGGCGGGGCGATCGGGCGGGGCGCGGCCATCCTGTCGGGGGCGCTGGAGAGCCGGGAGGCCGCGGAGGAGCGGCGGCACAGGGAGCTGATGGCCGTGGAGGAGCGGCGGAGCGTGGCACGGCAGGCGCGGCGCGAGGCCGGGGAGCAGTGCGTGGCCGGGCTGGCCGCTGCCGTGAGCCAGCTCGCCGGCTCCATGTTCGCGCTCGCCGCCAAGCGCAAGGGCCCCGCCGCGCCCAAGTGA |
|  | Protein | MDMKQQGSAGVMERSGQQGDQDAGRNLSAAGAMTLATVREYRRGNWTLPETMVLIEAKKRVHSERHPADQGLARWRWVEDYCWRAGCRRSQNQCNDRWDNLMRDYKKVRAHEQQHAAASSYWAMARAERKERCLPSNLLREIYEAMGEIVESRRRLSCYGGSGSGSGVLFLGAPLTATNVGADIPAMQASPLAQVPPPRPFEQGTHCSASESPERKRLRQSLDGQPRSSTPAAASASAGIHHQEAQPLQEDDESSGEDEDALGGAIGRGAAILSGALESREAAEERRHRELMAVEERRSVARQARREAGEQCVAGLAAAVSQLAGSMFALAAKRKGPAAPK |
| BdGT2 | CDS | ATGTCTGCCACCCGCCGCCGCCGCCCCTCGCCGCCTCCGCCGGCGTGGACCCCGGAGCCGTGGACCGACGGGGAGACCTCCGCGCTGCTCGAGGCCTGGGGCCCGCGGCACCTCCGCGCCGGCGGCGGGCCCCTCCGCACCGCCGACTGGCGCGCCTGCGCCGCCGCCGTCACCGCCCGCCGCGCCGCCGCCGGCCGCGCGCCGCGCACCGTCGACCAGTGCAAGAACCGGATGGATTACCTCAAGAAGCGCCTCAGGGCCGAGCGCTCCAGGCCGAGGGGGGCCGACCCGCCGCCGCCGCCGCTCTCGGGCTCCCTCGCCCGGCTCCGCAAACTCCTCAGCCAAGCCCCCTCGGTTCCCCACGGACTCGCGCCCCGCGTCACCGTCACGCCCAAGTCGGAGGAAGAGGACGACGACGACGACGAGGGGGAGGAGCAGAACGGCGGGGCTCCTCTGCACCGCGACTGGCCGCCGGTCCCCAAGCGGCGGCGGACCGCGGTGTCCTTGTCGCCGGTGAGCTCCGCCGCCGAGGGCCACCATCACGAAAACGGAGGGGTAGGCTGCACGGAGGTGGCGGCGGCTCTCGATAGGCTGGCGGGGACGTACGAGCGGGTTGAAGCGGCGAAGCAGAAGGAGGCGGCGCGGCTGGAGGAGCGGCGCCTGGAGGCCATGCGCGACCTAGAAATCGGACGGATGCGAGTTCTCGTCGACGTCGCCGTCCCCACCTCCGCCGGTACCGACAAAGCCGCCGCCGCCGCCGCCACACCGGCGGCGGCCTAG |
|  | Protein | MSATRRRRPSPPPPAWTPEPWTDGETSALLEAWGPRHLRAGGGPLRTADWRACAAAVTARRAAAGRAPRTVDQCKNRMDYLKKRLRAERSRPRGADPPPPPLSGSLARLRKLLSQAPSVPHGLAPRVTVTPKSEEEDDDDDEGEEQNGGAPLHRDWPPVPKRRRTAVSLSPVSSAAEGHHHENGGVGCTEVAAALDRLAGTYERVEAAKQKEAARLEERRLEAMRDLEIGRMRVLVDVAVPTSAGTDKAAAAAATPAAA |
| BdGT3 | CDS | ATGCAGCAGGAGCCGACGCCTGAGATGCCGCCATTCTCGCCGGCCGTGTGGCGGGGGCCCGCCGGGGCGCCGTCGCCCATCAGCAGCCGTCCGCCGGCGCCGGGACAACAGCAGCAGATGGTGGCGGCGGCCGACGAGCTCGGCGGCGGCGGCGGCGCGGCCGCGAGCGCGTCCGGGTCCGGCAGCCTGGACGGCGAGGAGGGCGGGTCGTCGGCGGCGGGGGGCAACCGGTGGCCGCGGGAGGAGACGCTGGCGCTGCTCAAGATCCGGTCCGACATGGACGCCGCCTTCCGCGAAGCCGCGCTCAAGGGGCCCCTCTGGGAACAAGTCTCCAGGAGGATCGGGGAGATGGGGTACAAGAGGAGCGGCAAGAAGTGCCGCGAGAAGTTCGAGAACGTGGACAAGTACTACCGCCGCACCAAGGACGGCCGCGCCGGCCGCGCACATGGCAAGACTTACCGCTTCTTCTCCGAGCTTGAAGCTCTCCACTCCATCTCCATCTCCTCCTCCGCCGCTCACCCGCCGCCCCTCCCCGCCCTCGCGCCACCGCCTTCGAAGCTCCCTGCACCCTCGCCGCCGCTCCCCACGGCTACATGCGCCATGGCAGCGCCGGGCGCGAGCTTGTCTTCGCTGGGAGGATCCGATGGGTCGGAAACTGATGACGACGAAATTGCGGCGGAGGGCTCCAAGAAGCGGAAGCGGCATAGCAGCAACTGGAGCGGCATTACTACTACTAGCAGCGGGGGCGGCGGCGGCAAGAAGAAGCAGGCGACGATGAGGCTTTTCGAGGGCATGATGCGGCAGGTGATGGAGCGGCAGGAGGCGATGCAGCAGCGGCTCCTGGACGCAATAGAGCGGCGCGACCAGGAGCGGCTCGCCCGCGAGGAGTCCTGGCGCCGGGGGGAGACCGCGCGCCTCGCCCGCGAGCAGGACGCGCTCGCCCGCGACCGCGCCGCCGCAGCCGCCCGCGACGCCGCCGTCGTCTCCTTCATCCACCGCGTCACCGGCCAGATGGCCGTCCTCCCGCCACCGCCGCCGGAGACGAAGACGGCGACCAAGGCCGCCATGCTCAAACGGCCGCCGCCAGCGCCGCCGTTGCAGCCGACCACGCCGCGGAATCAGCCCCAGCAGCCGCACGTCGTCATGCCGATGTCGGCGCCGGCATCGGCGTCGGCACCGGCACGGGCAGAGACGTCGCCGGCGCAGAGCAACGAGGTGGTGGCTGTCGGCGGCGTGGACACGGCGGCTGCGGCGTCGAGGTGGCCGAAGGCGGAGGTGCACGCGCTGATCGAGCTTCGGACGGAGATGGAGGCGCGGTACGGCAATGGCGGCGGCGGCGGCCATGAGACGCCCAACAAGGGCCCGCTGTGGGAGGACATCGCGGCGGGGATGCGGCGGCTCGGGTACGCCCGGAGCTCGAAGCGGTGCAAGGAGAAGTGGGAGAACATCAACAAGTACTTCAAGAAGGTCAAGGAGAGCAGCCGGAGCAAGCAGCGGCCCGTGGACTCCAAGACCTGCCCCTACTTCCACCAGCTCGACAAGCTCTACCGCACCAAGGCACAGAATGCTGCCGCCGCGTCTCAGAGCCCGACCGCGGTCACCGTGCTCGCCGCCGTGCCGCTCTCGTCCCAGACGCCGGCGCCGCATATCGTCGAGCACGGACCAGGCAAGAATCTCAGCAATGGTAATGGCAACAGCAACGGCATGCAAGCGAAGGCGAGCAATGGCAATGGCACAGCGGCGGCGAACAAGCTGGAGTCGAAGCAAGACGGCATGATCACGAAGGAGACGACGACGGAGCAGCATCCCCAGCCGGCGGGCAGCCCCATGGCGCCCATGAACCACAACCTCAACAACTACGGCAACGGCAGAACGGAGGTCATGCACGACGACATGGACGACATCGACAGCATGGACGACGACGACGACTTCGACAGCGACGACGAAGACGACGCCATTGGCGGCGGCCGCAACGAGCAGGTGCAGTCGTACGACATTCGTTTCCAAAGCCAGCAGCAGCAGCAGCAGCATCAGAACACTAATGGCGGCGGCAACCCTCCGGCGGCGCCTGCATCAGCGGCGGCGGCAAGTGGGGCGGCGCCGGCGCCGTTCTTGGCCATGGTCCAGTGA |
|  | Protein | MQQEPTPEMPPFSPAVWRGPAGAPSPISSRPPAPGQQQQMVAAADELGGGGGAAASASGSGSLDGEEGGSSAAGGNRWPREETLALLKIRSDMDAAFREAALKGPLWEQVSRRIGEMGYKRSGKKCREKFENVDKYYRRTKDGRAGRAHGKTYRFFSELEALHSISISSSAAHPPPLPALAPPPSKLPAPSPPLPTATCAMAAPGASLSSLGGSDGSETDDDEIAAEGSKKRKRHSSNWSGITTTSSGGGGGKKKQATMRLFEGMMRQVMERQEAMQQRLLDAIERRDQERLAREESWRRGETARLAREQDALARDRAAAAARDAAVVSFIHRVTGQMAVLPPPPPETKTATKAAMLKRPPPAPPLQPTTPRNQPQQPHVVMPMSAPASASAPARAETSPAQSNEVVAVGGVDTAAAASRWPKAEVHALIELRTEMEARYGNGGGGGHETPNKGPLWEDIAAGMRRLGYARSSKRCKEKWENINKYFKKVKESSRSKQRPVDSKTCPYFHQLDKLYRTKAQNAAAASQSPTAVTVLAAVPLSSQTPAPHIVEHGPGKNLSNGNGNSNGMQAKASNGNGTAAANKLESKQDGMITKETTTEQHPQPAGSPMAPMNHNLNNYGNGRTEVMHDDMDDIDSMDDDDDFDSDDEDDAIGGGRNEQVQSYDIRFQSQQQQQQHQNTNGGGNPPAAPASAAAASGAAPAPFLAMVQ |
| BdGT4 | CDS | ATGGAGAGAGGCGACGGAGCAGCAGCAGGAGGAGGAGGGATGGGGCCGCGGGGCGCGCCGGCGGCAATGCTGGGCATGGGGATGCAACAGTTCGTGTCGCAGCCGCACGCCGCCGCCCCCGTGTTCCAGCAGCCGGAGCACCTCCACGGCGGCGTCTTCGGCCAGCACCACCACCAACCCGTTCCGGCGCCGGCGAGGCAGCAGCCGCCGTCGTACTCGCCTTATCCGGCGGTGCCGGTCAGGGCGGGAGGAGGCCATCACGAAGAGGAAGCCATGGGCCACGGCGCCGGGAACGATGGCGTCGCGGCGCAGCAGCAGCAGCAGCCGGGGGGCCTCTGGTCGCGGATGAAGTGGACGGACGCGATGGTGCGCCTGCTGATCATGGTGGTGTACAACGCCGGGGATGACGGGGAGGGCGCCGTGGCCGCCGCCGTTGGAGGAGGAGGAGGAGGAGGAGGAAGCAGGGCGGCGGCGCATGGGCACGGGCATGGGTCGGCGACGGCGGCGGCGCACGCGCAGCAGAAGAAGGGCAAGTGGAAGTCGGTGTCGCGGACGATGGGGGAGCACGGCTTCACAGTGTCCCCGCAGCAGTGCGAGGACAAGTTCAACGACCTCAACAAGCGGTACAAGCGCGTGGTGGACCTGCTGGGCAGGGGCAAGGCGTGCGCCGTGGTGGAGAGCCCCGCGCTCCTGGACGCCATGGACGAGCTTCCCCCCCGGGCCAAGGAGGAGGCCCGCAAGCTGCTCAGCTCCAAGCACCTCTTCTTCCGCGAGATGTGCAACTACCACAACTCCCCCCACCCCCACGCCGCCGCCGCCGTTACGGTCGCCTCGCACCACGGCGCCGCCGTCCACGACCACGAGGGCGCCGCAGCATGCTTCCACCACCCGCAGCCGGTGGCATGTGCCTCGTCTGCTGCCGCTCTCCATGCTCTGGCTCCTTCCCCGGCGATGATGAACTCCTCCACGCGCACAGAAGGGGATGAGGAGGACGACGACAGCGAGAACGCGCACCCCCGAACAAGCAACGAGGTTGAAGAGATGGACGAAGAAGACGTCCTGGACGACGAAGAGGAGCAGGCGCCGGGGATCAAGAGCAAGCACCGCCGCTTCCACAGCCTCAACAGCAACGGGTTCCCCAAGCGCCGCCGGGGCGAGAGCAGCACGATGGAAGCAGAAGAGGACGGGAACAACAACGACAACACGGGGGCGGGCGAGGGGGAGGCGCCGTCGTCCGCGGGCGTGCAGCATCTCCAGAGCGAGCTGGCGGCCGCGGCGGGCGGCGGGGACCCGGAGCAGGCGCGGCGGTGGATGCGGCGGCGCGCGCTGGCGGTGGAGGAGCAGCTGCTGGCGTGCGACTACCGCGAGTACAAGCTGCACCGCCAGCGGCTCAAGTGGGAGCGCTTCTGCGCGGGCAAGGAGCGCGAGATGGAGCTGGCCAAGCTGAGGAACGAGCGCGCCCGCATCGACGGCCGACGCATGCTGCTCATGATCCAGCACAAGGAAATGGACCTGCTCAGGGGCTCCTCAACCGCCGGCGAGGCCTGCAACAACTCCTCCTCTGTTGACCACAACCCGCGCCAGACCCCGCCGTTCTCCGCCTTCCAGCAGCAGCAGCAGCTCGGCTCCAGCCCCTCCACCGCCGGCGGCCACCACAGCTAG |
|  | Protein | MERGDGAAAGGGGMGPRGAPAAMLGMGMQQFVSQPHAAAPVFQQPEHLHGGVFGQHHHQPVPAPARQQPPSYSPYPAVPVRAGGGHHEEEAMGHGAGNDGVAAQQQQQPGGLWSRMKWTDAMVRLLIMVVYNAGDDGEGAVAAAVGGGGGGGGSRAAAHGHGHGSATAAAHAQQKKGKWKSVSRTMGEHGFTVSPQQCEDKFNDLNKRYKRVVDLLGRGKACAVVESPALLDAMDELPPRAKEEARKLLSSKHLFFREMCNYHNSPHPHAAAAVTVASHHGAAVHDHEGAAACFHHPQPVACASSAAALHALAPSPAMMNSSTRTEGDEEDDDSENAHPRTSNEVEEMDEEDVLDDEEEQAPGIKSKHRRFHSLNSNGFPKRRRGESSTMEAEEDGNNNDNTGAGEGEAPSSAGVQHLQSELAAAAGGGDPEQARRWMRRRALAVEEQLLACDYREYKLHRQRLKWERFCAGKEREMELAKLRNERARIDGRRMLLMIQHKEMDLLRGSSTAGEACNNSSSVDHNPRQTPPFSAFQQQQQLGSSPSTAGGHHS |
| BdGT5 | CDS | ATGGAGGTGAGGGAGATGGCTGCGGCTGCTGCTGCGGCGGCGGCGGGAGGAGGGGGAGGAGGAGGAGGGGGAGGGTTTGGGAGGCTGCCGCCGCCCAACCCTAACCTGCCCTACCGGGAGGACTGCTGGAGCGAGGGCGAGACGGCGGCGCTGGTGGACGCGTGGGGCAGCCGCTACGTGGAGCTCAACCGCGGCAACCTGCGCCAGAAGCAGTGGCAGGAGGTGGCCAACGCCGTCAACTCGCGCCGCGGGGCCGCCGCGCGCCGCCGCCCGCCGCGCACCGACGTGCAGTGCAAGAACCGCGTCGACACCCTCAAGAAGAAGTACAAGGCCGAGCGCGTCCGCGGCGCGCCGTCGGGCTGGAGCTTCTTCGACGAGCTCGACCGACTCGTCGGCCCCACCCTCAACTCCGCCGCCAACAAGCGGCCCTCGCCGGCGATGGCCCCGCACCTCGCCCTGCCCGTCCATCCTTCTTCTACCGCCGCGAGGAAGTACCCGTCGCCTTCTTCCTCGCCGTCCCCGCCCCCGCCCATGGCTCTGCCCCTCCCCAGCTACCGCCGCGGGGCGCTGCTCCCTGCCGCTGCGTTCATCCAGCAGGAAGCCGCTGCCGCCGCTGCAGCGGTGTCTGATTCGGAGGACTCTGGTGATCATCCTGGTGGTAACAACAACAACAACCACCATGACAACTTGCGGAGGTCACCGTCGCGTTCTGTCTCGTCGGCGCGCTCCGGCGGTAGCAACAAGCGTCCCTTTCGTCGCGAGGCTGGCGGTGATGGGGATAGTGGTGTCAGCGAGCTGGCGAGGGCAATTGAGGCTTTTGCAGAGATGTATGAGCGCGTGGAGAGTGCGAAACAAAAGCATTCGCTAGAGATGGAACGCGAGCGAATTGACTTCCTGAAGCAGCTGGAGGTGAAGCGCATGGAGAACTTCGTCGATGCGCACGTGAAGCTCGCAAGGGTGAAGCGCACGAAGAAGAGCGGAGGGGCTGCATCCAATGGTGCTGGTGGAGTGGAGCTGATTTCATCCGTTGCTGCGCTGCCTTTCCTTTCCAACTCCAATTACCTCTGA |
|  | Protein | MEVREMAAAAAAAAAGGGGGGGGGGFGRLPPPNPNLPYREDCWSEGETAALVDAWGSRYVELNRGNLRQKQWQEVANAVNSRRGAAARRRPPRTDVQCKNRVDTLKKKYKAERVRGAPSGWSFFDELDRLVGPTLNSAANKRPSPAMAPHLALPVHPSSTAARKYPSPSSSPSPPPPMALPLPSYRRGALLPAAAFIQQEAAAAAAAVSDSEDSGDHPGGNNNNNHHDNLRRSPSRSVSSARSGGSNKRPFRREAGGDGDSGVSELARAIEAFAEMYERVESAKQKHSLEMERERIDFLKQLEVKRMENFVDAHVKLARVKRTKKSGGAASNGAGGVELISSVAALPFLSNSNYL |
| BdGT6 | CDS | ATGGATCCCTTCCACCACCTCAACACCGCCTTCTCCAACCCCTACCACCCGCTCCTCTCCTCCTCCCCTCCCCACCCCCACTACCCTCCCCCGCTCCCGCCGCCGCCAGCCGCGGCCGCGGAGCCGCCGGAGCGGGAGCGGCTGCCGCAGTGGTCCCACGCCGAGACGGCCGCCTTCCTCGCCATCCGCGCCGACCTCGACCGCTCCTTCCTCTCCACCAAACGCAACAAGGCGCTCTGGGAGGCCGTCTCCGCCCGCCTCCATGACCATGGCGGGTTCGCGCGTACACCGGATCAGTGCAAGTCCAAGTGGAAGAACCTCGTCACCAGGTTCAAGGGCTCCGCCCACCCCCCGCCGCCGCCCGACGCCGCCGACACGCCCCAGCACCAGCACCACCCGGGTGACCATCCCGGTACCGGCGGCAGCGGCGGCGCTTCGTCTGCGGCGAGGGGCGGCGGCTTCCCGTTCCACGACGAGATGAGGCGGATCTTCGACGCCAGGGTCGAGCGGGCGCGCGCATTGGAGGCGAAAAAGGCCAAGGGGAAGCACCACGCGCGGTCAGGGCACGATCATCTTCATCCGGATGATGCCGACGACGATGGCCAAGGGGATGAGGCCGGCGAGGAAGAAGACGAGGAGGCGGACATCCTGGAAGACGAAGAAGAGGAGAGTCGGGCAGCGGAGACGACGAGGGGTGCTGCTGGGCCTGGGAAGAAGCGGAGGAGGAAATCAACAGCGGCGCCCGCGGGGAACGAGCAGGGCGAGGTGGAGGCGATGCTGCGGGAGTTCATGCGGCGGCAGGCGGAGATGGATGAGCGGTGGGCGGAGGCCGCCGAGGCGCGCGACGCCGAGCGGCGCGCCCGCGAGGAGGAGTGGCGGGCCGCCATGGTCGCGCTCGGCGAGGAGCGGCTCGCGCTCGTGCGCCGCTGGCGGGAGCGCGAGGACGCGTGGCGGGCGCGCGCCGAGGAGCGCGAGGAGCGACGCCACCGGCTCATCGCCGCCCTGCTCGCCAAGCTCGGCGGCGGCGACACGTCCTGA |
|  | Protein | MDPFHHLNTAFSNPYHPLLSSSPPHPHYPPPLPPPPAAAAEPPERERLPQWSHAETAAFLAIRADLDRSFLSTKRNKALWEAVSARLHDHGGFARTPDQCKSKWKNLVTRFKGSAHPPPPPDAADTPQHQHHPGDHPGTGGSGGASSAARGGGFPFHDEMRRIFDARVERARALEAKKAKGKHHARSGHDHLHPDDADDDGQGDEAGEEEDEEADILEDEEEESRAAETTRGAAGPGKKRRRKSTAAPAGNEQGEVEAMLREFMRRQAEMDERWAEAAEARDAERRAREEEWRAAMVALGEERLALVRRWREREDAWRARAEEREERRHRLIAALLAKLGGGDTS |
| BdGT7 | CDS | ATGGACGGGAAGCTGCCGCCGCCGAATCCGAACCTGCCGTACCGCGAGGACTGCTGGAGCGAGGGCGAGACGTCAGCGCTCGTCGACTCCTGGGGGTCGCGCTACCTCGAGCTCAACCGCGGCAGCCTCCGCCAGCCGCAGTGGCGGGAGGTCGCCGACGCCGTCAACTCCCGCCCGGGGGCCTCCGCGCGCCGCCGGCCCCCGCGCACCGACATCCAGTGCAAGAACCGCGTCGACACCCTCAAGAAGAAGTACAAGGCCGAGCGGGCGCGTGGGACGACATCGCCCTGGCCCTTCTACAACCAGCTCGACATCCTCGTCGGCCCCACGCTCTCCGCCTCCGCCGCCGCCGCCAGCAAGAAGCCGTCCCCGCCCCGCGGCCTTCCCATGATGATGCGGAGGCGCCCGTCGCCGTCCCGCTCCCCGTCACCGCCCTCCCCGGCGCCGCCTATGGCGCTCCCCTTGCCCAACTACCGCCGCGGGTCCAACCTCCCGTCCGCCGACCTCTTCCATAAGGCGGCGGCGGCCGCCGCCGCCGCCGTGGATTCTGACTCGGACGATGGCTACAACAACAACAACAACTATGACGACGACGAGGGCTCCCAGCAATCGCCCTCGCGTTCTGTTTCGTCTCGCTCTGGCGGTAATGGTGCTCCTGCTCTTGGCGGAGGTGGCAGCAGCAAAAGGAAGAGAGGTGGGTCTGGAGGGTTTGGGGAGCTGGCCATGGCAATGGAGACTTTCGCTGAGATGTATGAGCGCATGGAGGCCGCCAAGCAAAGGCATGCGGAGGAGATGGAGAGACAGAGGATAAAGTTCCTCAAGGATCTGGAGCTGAAGCGGATGCAGGCTTTTGTGGACATGCAGCTTCAGCTTGCGAGGGTGAAGCACGCCAAGAATGGTACCTCAGAGATGCTCATGTCTCTAGCCGCACTGCCATTCCTCAGCAACCCTGCTTACCTTTGA |
|  | Protein | MDGKLPPPNPNLPYREDCWSEGETSALVDSWGSRYLELNRGSLRQPQWREVADAVNSRPGASARRRPPRTDIQCKNRVDTLKKKYKAERARGTTSPWPFYNQLDILVGPTLSASAAAASKKPSPPRGLPMMMRRRPSPSRSPSPPSPAPPMALPLPNYRRGSNLPSADLFHKAAAAAAAAVDSDSDDGYNNNNNYDDDEGSQQSPSRSVSSRSGGNGAPALGGGGSSKRKRGGSGGFGELAMAMETFAEMYERMEAAKQRHAEEMERQRIKFLKDLELKRMQAFVDMQLQLARVKHAKNGTSEMLMSLAALPFLSNPAYL |
| BdGT8 | CDS | ATGGACGACCCCGCCGCCGCTTCCCTCTCTTCGTCGCCCTCCTCCTCCGACGCCTCCCCCTCGCCACGCTCCAAGCGCCGCCGCACCGACCGCTACGCGCTCGGCTTTGAGTTCGCCCCGCGCCTTACGCCCTCGGCCGCTGCTCCGGCCCCGGCCTCGCGTAGCGCGTCCGAGTGGACGGAGGACTCCACCTTCGCGCTCCTCGACGCCTGGGGCGAACGCTTCGTCTGCGCCGGCCGCCGCAGCCTCAGCGCCGACGAGTGGCTCGAGGTCTCCCACCTCGTAGCAGCCGCCGCCTCCCGCCCTGCTGGATACTACTCCGAGGCGCAGTGTCGCAACCGCATCGACACCCTCAGGAAGATGTTTAGGAAAGAGAAGGAGAGGTCCCGCCTGGCTGCGCACCGCTCCAACCACCCCTCGCCCTCCAATTGGGTCTACTTCGACAAGATGTTGTCCCTCATGTGCCCAACGCCGCTACCGTTGCTACCTCGTATCGTGAAGCGCCGTCGCGACAGACAACCAGTGCCGCGTTATTCGTGGGGTGTGGATGTCGGGGAGTTTGTGCTGGCCGGATGTGGCGGTGCAGGGTCGGATGCGGAATTGGGGGGTGATGTGACTGGTGCACTGAAGGGGGAGGAGTTTGCAGTGCTGACGGAGTCAATTCGGAGGTTTGGGGAGGTTTACGAGAGGGTGGAGAGTAGCAAGAGGCAGCACATGGCGCAGTTGAAACGAATGCGGAGGGATATGCAAAGGGACCTTGAGGTGAGGTGGAGAGAGATATTGGAGAAGGCAGAAATGGAGATTGAAAGTATTGAGGAGGAGGAGGAGGGGGGGGATAATGCTAAGAAGAGGTTAGGAGACGACGATGGTGGTGAAAAGCATAACAATGGAGCTGTGGATGCTTCTCCGTAG |
|  | Protein | MDDPAAASLSSSPSSSDASPSPRSKRRRTDRYALGFEFAPRLTPSAAAPAPASRSASEWTEDSTFALLDAWGERFVCAGRRSLSADEWLEVSHLVAAAASRPAGYYSEAQCRNRIDTLRKMFRKEKERSRLAAHRSNHPSPSNWVYFDKMLSLMCPTPLPLLPRIVKRRRDRQPVPRYSWGVDVGEFVLAGCGGAGSDAELGGDVTGALKGEEFAVLTESIRRFGEVYERVESSKRQHMAQLKRMRRDMQRDLEVRWREILEKAEMEIESIEEEEEGGDNAKKRLGDDDGGEKHNNGAVDASP |
| BdGT9 | CDS | ATGGCGGGGGCGACGACGCCCCGGTCGCGGCTGCCGCGTTGGACGCGGCACGAGACGCTGGTGCTGATCCAGGCGAAGCGCGCGACGGAGCAGAGGGCGGCGGCGGCGGCGCAGGGGCAGCCGGTGCGGCTCAAGTGGGCGGTGGTGTCGGGGTACTGCCGGCGGCACGGCGTGGAGCGGGGACCCATGCAGTGCCGCAAGCGCTGGGGCAACCTCTCATGGGACCTCAAGAAGATCCTCGTCTGGGAGAAGAAGAACACGAAAACGAAGACGTCCATGGAGAAGGAAGGGGAAGAAGAGGAAGAAGAAGAGAAGAAGGGGGAAGGGCAGGACGAGTCTTCGTCCTCGCGGCCGTCGTTCTGGGAGATGCGCGGGGAGCAGCGGCGCGCCAGGCAGCTGCCGTCGTCCTTCGACCGGGAGGTCTACGACGCGCTCGTCGGCGCCGCCGCCTCCGTCAAGCTCCCGCCGGCGGAGTCCGGCGAGGAGATGGATCTCGACGACGACGAAGACGAGGACGGCGGCCATCAGCAGATTCCGATGCCGATGCCGATGCCGGCCCAGACGCCGCCGCCGCCGCCGGTCTTGGTCATGCCCATCTCAGCCAGGATGTACGTCCCGCCGGCCTCCTCCCAGCACGAATGCTCCGATCCAGCGACGGAGAGCGCGAAGAAGCCGGGGTCGGACAAGAACTCGACGTCGCAGCAGGACAGCGAGGCCACATTCGGCGCCGGAGTCGGGGCAGAAGGCACCGCAACGGCCGCGACAGCGACGACGAGCGTGATCGGGAGACAGGTGACGGAGGCGCTGGAGAGGGGCAACCGGGTGCTGGCGGAACAGCTGGAGGCGCAACGGGCGAGCGCGGAGTCCGACAGGGAGCACCGGGCGGCGCTGCTCGCCGCCGTCAACCGCCTCGCCGACGCCGTCTCCAGGATCGCCGACAAGCTCTGA |
|  | Protein | MAGATTPRSRLPRWTRHETLVLIQAKRATEQRAAAAAQGQPVRLKWAVVSGYCRRHGVERGPMQCRKRWGNLSWDLKKILVWEKKNTKTKTSMEKEGEEEEEEEKKGEGQDESSSSRPSFWEMRGEQRRARQLPSSFDREVYDALVGAAASVKLPPAESGEEMDLDDDEDEDGGHQQIPMPMPMPAQTPPPPPVLVMPISARMYVPPASSQHECSDPATESAKKPGSDKNSTSQQDSEATFGAGVGAEGTATAATATTSVIGRQVTEALERGNRVLAEQLEAQRASAESDREHRAALLAAVNRLADAVSRIADKL |
| BdGT10 | CDS | ATGCAGTCGGGGTACGGAGGGGTGTCGGAGTTCCAGCAGTTCATCATGGACGGCGGCTTCGCCATGGCCGCCGCGCCGCCGCCGCCTCCTACTCCGCAGCAGCAGCATGCGGCGGCGGCGGCGGCGGCGGCCGGAGGGCAGCAGGAGCTCGGGGCGCCGTTCAGGTACCAGCCGCTTCACCACCACGCCATGCATCCGCAGCAGCACCACCACCACGCGCCGCCCCAGATGCCGCCGCACTTCGCGCACTTCGGTGGCCCCACGGCGGCCGGCGGCGGGATCCCGTTCACGCAGCAGCTGCTCCACCACCAGGCGGCCGGGCAACAGCACCACCACCACCATCTCCAGCTCTTCCACGAGCAGCAGCATCACCACCACCAACAGCAGCAGCAACATCACAAACCTCAGCAGCCCCAGCCGAGGTGGGCGCCGCAGCAGCAGCAGCACCACCAGCACCAGCAGCAGCATCTGGGGCTGGACATGGAGGCGGCCGTGCCGGAGAGCACCCGCGCGGCGGCGGTTCCTTCTGGGATGCCGCCGTTCCTGGCCGCGGCCATGAACTTCAAGCTCGCGGTCGACCAGAGCGGCAGCGGCGCCACGGACGACGCCATGAACGACGGGGGCGGCGGGGGGAGCGGCATGATGCTCCACGGAGGAGGAGGGGGAGGCGCCGGACGCGGAGAAGACGAGGCGGCGACGGAGAGCCGGCTCCGGCGCTGGACCGGCGAGGAGGAAGCCTCCATCAAAGAACCCGCCTGGCGGCCGCTGGACATCGACTTCATCCATAGCACAAGCAGCAGCAGCAAGAGAGCCGCCGGCAAGGACAAGGCGCCCACGCCAGAGTCGCCGTCACCGGCCGCCAATGCCGCGGCCAATTACTTCAAGAAGGGCGGCGACGACAACGTAACCGCTAATGCGGCGGCGGCGGGCGGCGGTAATAGCAGCTACAAGCTGTTCAGCGAGCTGGAGGCCATCTACAAGCCGGGCAGCGGCGGCGCCGGACAGACCACCGGCTCCGGCTCCGGCCTGACCGGCGACGACAATGCCATGGCCGACCTCCCACACGCGCCTCCGCCCACAATGTGCCCCGACGGCGACGACGACATACTCCCCCATGCGCCGCCGCCGCAGCCGCAGGCCAACGCGTCGGAGACCTCCGCCGGAGAAGAAACAGAAACCCCTGCCGTGGTCCAGCCTCCTCAACCGCCGGCGTCAATGGCGGACGGGCGGCGCAAGCGGAAGCGGCGGCGGAGCCAGGAGGCGGCGACGGCGGCGTCGTCCTTCTTCGAGCGTCTGGTGCAGCGTCTGATGGAGCACCAGGAGTCCCTCCACGCGCAGTTCCTTTCCGCCATGGACCGCCGCGAGCGCGAGCGCGCCGCCCGCGACGAGGCCTGGCGCCGGCAGGAGTCCGAGAGCTCCGCCCGCGAGCGCGCCGCCCGCGCCCGCGACCGCGCCTCCGCCGCCGCCCGCGAGGCCGCCATCGTCTCCTACCTCGAGAAGCTCTCCGGCCACCCCATTGTCCTCCCTCCCCCTGCTCCGTCTTCCCCAGCTCCGGCGGCCTCCGAAGAAATAATCATCCCCCAGCCCGAGGCCAGCAGCGCCGTGGGCACGGAGCTCGTGCCGTACGACGCCGGCGTCCTGAGGTCGTCTTCGGCCTCGCCGTCGCGGTGGCCGAAGCAGGAGGTGGAGGCGCTGATCAGGGTGCGGTCGGGGCTGGAACGGCGGTTCCAGGAGCCAGGGCTGAAGGGGCCGCTGTGGGAGGAGGTGAGCGCGCGCATGGCGGCGGCGGGGTACGGCGGCCGGAGCGCGAAGAGATGCAAGGAGAAGTGGGAGAACATCAACAAGTACTTCCGCAAGGCGAAAGAGAGCGGCAAGAAGCGGCCGGCGCACGCCAAGACTTGCCCTTACTTCGACGAGCTCAACCGCCTCTACTCCGGCCGCGGCGGCGAAGCCATGGATGCTGCCGGCGATGGTGGCGCGGGCAAGGCGGCGAGCTCGGAGCTGCTGGACGCCGTTGTCAAGTGCCCTGCGGGAACGGCGGCGGGGGATCAGGCGGCGCAGTACGGCCCGCCGCCCGGGTTCGCGCCCGCCGGAGATGGGGAAGACGAAGGCCTCCGTGACATGGCCGTGGATGGCGGCGAGAAGTACGACGATGGGGAAGACGGTGAGATTGGGAGATCGGCGGGCGGGAGAGCCATGGCGATGGCGGATCAGGATCAGGACGAAGGTGAGAGCCATGGCGGACACCACGACGAGGACGACGAGGAACACCATTAG |
|  | Protein | MQSGYGGVSEFQQFIMDGGFAMAAAPPPPPTPQQQHAAAAAAAAGGQQELGAPFRYQPLHHHAMHPQQHHHHAPPQMPPHFAHFGGPTAAGGGIPFTQQLLHHQAAGQQHHHHHLQLFHEQQHHHHQQQQQHHKPQQPQPRWAPQQQQHHQHQQQHLGLDMEAAVPESTRAAAVPSGMPPFLAAAMNFKLAVDQSGSGATDDAMNDGGGGGSGMMLHGGGGGGAGRGEDEAATESRLRRWTGEEEASIKEPAWRPLDIDFIHSTSSSSKRAAGKDKAPTPESPSPAANAAANYFKKGGDDNVTANAAAAGGGNSSYKLFSELEAIYKPGSGGAGQTTGSGSGLTGDDNAMADLPHAPPPTMCPDGDDDILPHAPPPQPQANASETSAGEETETPAVVQPPQPPASMADGRRKRKRRRSQEAATAASSFFERLVQRLMEHQESLHAQFLSAMDRRERERAARDEAWRRQESESSARERAARARDRASAAAREAAIVSYLEKLSGHPIVLPPPAPSSPAPAASEEIIIPQPEASSAVGTELVPYDAGVLRSSSASPSRWPKQEVEALIRVRSGLERRFQEPGLKGPLWEEVSARMAAAGYGGRSAKRCKEKWENINKYFRKAKESGKKRPAHAKTCPYFDELNRLYSGRGGEAMDAAGDGGAGKAASSELLDAVVKCPAGTAAGDQAAQYGPPPGFAPAGDGEDEGLRDMAVDGGEKYDDGEDGEIGRSAGGRAMAMADQDQDEGESHGGHHDEDDEEHH |
| BdGT11 | CDS | ATGGATCCCAACGACGCCGGCCTGCCGTGCATCGTCCAAGCCCTGGCCTCCATCGATCCCAGCGACACCACCGTCCTGCCCTGCATCGTCCAAGCCCTAGCGGCGCTCCCCCCGTCGCCACCCACCCCCAAAACCCCAGCCCCTGCCACTCCCAGAGCCCAGCCGCCGCCGCCGCCCGCCACCGAATCCTCCAACTCCATGCCCCCGAGCCCCCGCCGCACGCGCTCCGGCCGCGCGCCCGAGTGGACCGCGGCCGAGACCCTCGCGCTCGTGGCCGCCGTCGCCGCCACGGACGACGGCTGGGCCCGCTCCGTCTCCGCCTTCCAGAAGTGGGCCATCGTCGCCGAGAACCTCGCCTCCTCCGAGGGGGCGGGAGGCTCGTCCTCCAGGCGCAGGGCGAGGCCCGCCGGCGACTGCAAGCGGAGATGGGAGGCTCTGGCCGCGGAGTACGCGGCGGTGCGGCAATGGGAGGCGCGGACCGGGGGCAGGTACTCGGAGATGGGCGCTGCGGCGAGGAGGAAGGCTGGTCTCCCCTCCGAGTTCGACACCGAGGTGTACGGCATCATGGACGCGCTCCTTGCGGATTCCGGCGATGCCGGGGGAGGTGGGGGCAAGGAGGGCTTGGTCGGTGCCGGTGGTGGTGGTGGTGCTGATGTGGGAGAAGAAGAGGGTGATGGTGTTGGAGTTGGAGAAGAGGAGGAAGATGACGATGACGACGATGAGAAGGAGGAGGAGTTGGAAGGTGATGACAATGAGGGGGAGATGCAGGTGGATGGTGGAAATGCGAATGCTTCTGATGATTTGGAATACTGCGAGACAGAAACACAGAACGAGCCGAAAAAGAGCCAGATAGAGGCATGGGAACTGGCCAACAAGTTAAATGATAATGCGCAGCATATCCATATGATATTAGAAGGCGAGGTCGGCGATGATGCTGGCCAGAATCATGCCCCTGCCGATCCAATCTCGCCCAACGCAATGGAGAGCACTCGCCAGAAAGCTGATGAGCTGATCAAATCGCTCGGTGGGCTTGTGAGCTACCTGAACCAATTCACTGATCTGATCAAAGATAACGGGTTTGAGGATGTAGTTGGTGTCAACTGA |
|  | Protein | MDPNDAGLPCIVQALASIDPSDTTVLPCIVQALAALPPSPPTPKTPAPATPRAQPPPPPATESSNSMPPSPRRTRSGRAPEWTAAETLALVAAVAATDDGWARSVSAFQKWAIVAENLASSEGAGGSSSRRRARPAGDCKRRWEALAAEYAAVRQWEARTGGRYSEMGAAARRKAGLPSEFDTEVYGIMDALLADSGDAGGGGGKEGLVGAGGGGGADVGEEEGDGVGVGEEEEDDDDDDEKEEELEGDDNEGEMQVDGGNANASDDLEYCETETQNEPKKSQIEAWELANKLNDNAQHIHMILEGEVGDDAGQNHAPADPISPNAMESTRQKADELIKSLGGLVSYLNQFTDLIKDNGFEDVVGVN |
| BdGT12 | CDS | ATGGAGGGAGCAGGAGCAGGAGCAGGGATGTGGCCGCAGGGGGTGCTGGCGTCGTCGGCGGCGGCAATGCTGGGCCTAGGCACGGGGCCGCAACAGCAACAACAACAACAAGCCGTCAGCCAGCTGCCGCAGTACTCCCTGCTGCAGCAGCTCCAGGGCGGCGGCTTCCAGCACCACCAACCCCAACCCGTGCCCATGCCGGTGCCGCCGGAGCCGGAGCCGGAGTCGCCGGTGCCGCCGGAACCGGAAGAGGAGACGACGGACGGCGCCGGCGCCGGGAACGACGATGGCGGCGTCCCGGGGGCGGGGTTCGTGTGGACGCGGGTGAAGTGGACGGACGCCATGGTCCGGCTCCTGATCATGGTCGTCTACAACGCCGGCCCCGTGGAAGACGCCTCCGGCGCCGCGGCGGAAGCGGGCAAGAAGAAGGCGTCTTCTTCGTCTTCGTCTTCGTCTTCGCGGGGAGTGCAAGTGCAGCAGGGGAAGAAGGGGAAGTGGAGGTCGGCGTCGCAGGCGATGTCGGAGCGCGGGTTCGCGGTGTCGCCGCAGCAGTGCGAGGACAAGTTCAACGACCTTAACAAGCGCTACAAGCGCGTCCTGGACATCCTGGGCAGCGGCATGGCGCGCGCCGTCCTCCAGAACCCCGCGGTCCTGGACGCCGCCATGAGCAAGCTCGCCCCCGCCGCCAGGGAGCTCGCGCGCAAGCTGCTCGGCCCCAAGCAGATCTTCGGCTTCCGCGAGATGTGCCTCTACCACAACGCCGTCGCAGCCACCGGCGCAGCCGCAGCCGCGTCGAAGCCCCCGAAGCCGGTAAAGCCGGTAAAGCCGGTGAAGCCAGTGGTCACCGCCGCTCTCCCGACGATCAACGAGGTTGAAGACGAAGAAGACGAAGAGGCCATGGGGTTCGGGCGCCGGAACCCGAAGCGGCGCCGCGGGGAGAGCAGCAAGGCGAAGGGGGTTGCCGTTGCCGAGGAAGAAGAAGAAGCTGATCCGGAAGAAACGGAGGAGGACGAGGATGCGCCATTGTTGTCCGCCATGGCCGTGCCGCAGCTGCAGAGCAAGCTGGACGCGATGGGGCCACCCGGCGGCGGCGGGGACCTGGAGGAGGCGCGGCGGTGGCTGCAGCGGCGCGCCATGGAGGTGCAGGAGAAGCTGAAGGCGTGCGACCGCCGCGACAAGGAGCTAGACGCGCACCGGAAGAAGTGGGAGCGGTTCCGGGAAGTCAAGGAGCAGGAGATGGACCTCGAGGAGCTGCGGATCATCCACGCACGACGCAAGCTGCTCGTGCTCAAGCAGAAGGAGCTCGACCGGAAGATCGGCGAGCGGCAGAGAGCGCGGGCCTTCCAGCAGAAGCAGAAGGAGAAGGAGAAGCAGGTGGGCTCCGGCCCGGCCTTTGGGGGCTGCTCAGTGCCGTTGTTTCCTACTCCTACTTGTGTTGCGCATTTTTCTTGA |
|  | Protein | MEGAGAGAGMWPQGVLASSAAAMLGLGTGPQQQQQQQAVSQLPQYSLLQQLQGGGFQHHQPQPVPMPVPPEPEPESPVPPEPEEETTDGAGAGNDDGGVPGAGFVWTRVKWTDAMVRLLIMVVYNAGPVEDASGAAAEAGKKKASSSSSSSSSRGVQVQQGKKGKWRSASQAMSERGFAVSPQQCEDKFNDLNKRYKRVLDILGSGMARAVLQNPAVLDAAMSKLAPAARELARKLLGPKQIFGFREMCLYHNAVAATGAAAAASKPPKPVKPVKPVKPVVTAALPTINEVEDEEDEEAMGFGRRNPKRRRGESSKAKGVAVAEEEEEADPEETEEDEDAPLLSAMAVPQLQSKLDAMGPPGGGGDLEEARRWLQRRAMEVQEKLKACDRRDKELDAHRKKWERFREVKEQEMDLEELRIIHARRKLLVLKQKELDRKIGERQRARAFQQKQKEKEKQVGSGPAFGGCSVPLFPTPTCVAHFS |
| BdGT13 | CDS | ATGCAGCAGCAGCAGGGAGGAGGAGGAGGGCCGGGGCAGCAGTTCGGCTTGCATCCGCCGGAGATGCCGCCATTCTCGCCGGCCGGGCAGCGGATCTCGATGGCGGAGGCGCCCTCGCCCATCAGCAGCCGCCCCCCGGCCCCACCGGGTCAGCAGCAGCTCAGCAGCAACGAGCTAGCGGGCGCCGCCGCCGCCATGAGCTTCGACGAAGAGGCGCTGGCGGCCGGCGAGGAAGGCGGGGGCGGCGGCTCCGGCGGCAACCGGTGGCCGCGGCAGGAGACGCTGGTGCTGCTCAAGATCCGGTCCGACATGGACGCCGCCTTCCGGGACGCCACCCTCAAGGGACCGCTCTGGGAAGAAGTCTCCAGGAAGCTGGCGGAGGAGGGGTACCGGCGGAACGCCAAGAAGTGCAAGGAGAAGTTCGAGAACGTGCACAAGTACTACAAGCGCACCAAGGACAGCCGCGCCGGCCGCAACGACGGCAAGACCTACCGCTTCTTCCAGCAGCTCGAGGCCCTCCAAGGCGCCACGCCCGGAGCCGGGGCGTCCTCGGTCCCGCCGCCGGCCACCGCCGTGCGGGCCCCGGCCGAGCCTCCGCCGCAGCCTGTTGTTGCTGGTGCCATGCCGACGCCGATGGGCGTCGGCAATCTGAGCTTCTCGACATCCAACACGGAGGAATTCTCCGAGGACGAGGACGAGGAAGATGACTCGGACGACGAGGGCACGGACGACATGGCCGTGGTTGGGAACAAGAGGAAGCGGATGTCGTCGGACGGAGTCGCGGCGGCGGGGGGGCATAATAACAAGAAGATGATGCGCTTCTTCGAGGGGCTGATGCGGCAGGTGATGGAGCGGCAGGAGGCGATGCAGCAGCGGTTCCTGGAGGCCATCGAGAAGCGGGAGCAGGACCGGATGATCCGGGAGGAGGCCTGGCGGCGGCAGGAGATGGCCCGCCTCGCCCGCGAGCAGGAGACCCTCGCCCAGGAGCGCGCCATGGCCGCCTCCCGCGACGCCGCCGTGCTCGGCTTCATCCAGAAGATCACGGGCCAGTCCGTCCCCATGCCCATGGCGCCGCCGCCGCCCTCCATCGCCTTCATGCCGCCACCGCCGGCGGGGTCCCACCCCACGCCCATCTCCTTCTCCGCCGCGCCGCCATCGTCGTCCCAATCGCCGGCCACGCAGGCCTCGCCGCGGCCGCAGAAGCCGCCAATGCCGCTGCCCACGCCGGCGCCGCAGAAGACGCCGGTGCCGGCCACACCGCCGCCGCAGCAGCAGAGCGGCGGCATGGAGATGGTGGTCTCGGCGCCGGCGGGCGGGGAGCTGCAGCTGCACGACGGCGGGTCGGGGTCGGCTTCGTCTTCGAGGTGGCCCAAGGCGGAGGTGCACGCGCTGATCCAGCTGCGGAGCAACCTGGACACGCGGTACCAGGAGGCCGGGCCGAAAGGGCCGCTGTGGGAGGAGATCTCGGCGGGGATGAGGCGGATGGGGTACAGCCGGAGCTCCAAGCGGTGCAAGGAGAAGTGGGAGAACATCAACAAGTACTTCAAGAAGGTCAAGGAGAGCAACAAGAAGCGGCCCGAGGACTCCAAGACCTGCCCCTACTTCCACCAGCTCGAGGCGCTCTACCGCAACAAGCAGCAAGCCGCGCTCACCTCGCCATCCGCCGCCGCGGCGCCCTTGCCGGCCCTTGCAGCTCCCCCGCCGCCGGAGCCGTTCACCGTCGCGGCGCCCATCTCGCAGACGCCGCCGACGACGACGCACGCCTTGCAGCAGCCGGCCGCGGCGAAGAACGGCGCGGGCAACGGGCACGGGAACGGGAACGGCAGCGGCGTCGCAGCCTGCTCAGAAGGAGGCAGCGTCTCGGCCGGTGGAATGCAGGCAAGCAACGGGTTCTTCGGCGAAGCCGGCGTGGCGGCGAAGAAGCCAGAAGGCATGATGAAGGAGACGATCATGGAGCAGAGGCAGCAGGCACAGGCGGCCCCGATCAGCAGCAGCTACAACAACCGAGCCGGCGTCGTCGACAGCGACAACAGCATGGACGACGAAGACGACTACGACGACGAAGACGAGGAGGACGACGATGACGACGACGTGGACGGGAACAAAATGCAGTACGAGATCCAGTTCCAGAGCCAGCAGCACCACCAGCTTCATCAACAGCACCACCAGCACAACAACCACAACGTCGTCAGGCCGAACGCCGGCGCGGCCGGCGGCGGCAACCAGCCAGGCGCCGCTGCTCCTCCCAGCGCGGCATCGGCGGCGGCCGCGACGACGACGGCCGGGTCTTTCCTGGGCATGGTCCAGTAG |
|  | Protein | MQQQQGGGGGPGQQFGLHPPEMPPFSPAGQRISMAEAPSPISSRPPAPPGQQQLSSNELAGAAAAMSFDEEALAAGEEGGGGGSGGNRWPRQETLVLLKIRSDMDAAFRDATLKGPLWEEVSRKLAEEGYRRNAKKCKEKFENVHKYYKRTKDSRAGRNDGKTYRFFQQLEALQGATPGAGASSVPPPATAVRAPAEPPPQPVVAGAMPTPMGVGNLSFSTSNTEEFSEDEDEEDDSDDEGTDDMAVVGNKRKRMSSDGVAAAGGHNNKKMMRFFEGLMRQVMERQEAMQQRFLEAIEKREQDRMIREEAWRRQEMARLAREQETLAQERAMAASRDAAVLGFIQKITGQSVPMPMAPPPPSIAFMPPPPAGSHPTPISFSAAPPSSSQSPATQASPRPQKPPMPLPTPAPQKTPVPATPPPQQQSGGMEMVVSAPAGGELQLHDGGSGSASSSRWPKAEVHALIQLRSNLDTRYQEAGPKGPLWEEISAGMRRMGYSRSSKRCKEKWENINKYFKKVKESNKKRPEDSKTCPYFHQLEALYRNKQQAALTSPSAAAAPLPALAAPPPPEPFTVAAPISQTPPTTTHALQQPAAAKNGAGNGHGNGNGSGVAACSEGGSVSAGGMQASNGFFGEAGVAAKKPEGMMKETIMEQRQQAQAAPISSSYNNRAGVVDSDNSMDDEDDYDDEDEEDDDDDDVDGNKMQYEIQFQSQQHHQLHQQHHQHNNHNVVRPNAGAAGGGNQPGAAAPPSAASAAAATTTAGSFLGMVQ |
| BdGT14 | CDS | ATGTCGTCGTCCTCGTCGCCCAAGCCTGACGCCGTCTCCTCCCCGGAGCTGCCCCCGCTGGCCGCGCCCAACATCGCGGCGGCGGCGGCCGCCGCCGCGGCGGCGGCCTCCTCGTCCGGCGGCATGGGGCTCTCCGGGTCCGCCGTCGGGGTCGGGTCCGGCCGCCGCCTGCCCCCTCCCTGCTGGACGCACGAGGAGACCCTGGCGCTCATCGAGGCCTACCGCGACAAGTGGGAGGCGCTGCGGAAGGGGAACCTGCGGGCCGCCGACTGGGACGAGGTCGCCGGCGCCGTGACCGCCCGCTGCGGCCACTTCCCCACGGCCACATACAAGTCCGGCGTGCAGTGCCGCCACAAGATCGAGAAGCTCCGCAAGCGGTACCGCGCCGAGCGGTCCCGTTCCGCGGGGAGGTCCAAGGGGCCCAAGTGGCCCTTCTTCCCGCTCCTCCACGACCTCGCTGGCGGGGGCGCGCCCGATCCCAGCCCCAACCCCATCATCAAGATCAAGCCCAGGGCCGGCGGCGGCACCCCCGCGTCTCCTTCTTCACCCATGTCCTCGCCCTCGTCCGAGGAGGCTGGCCGGAGCAGGAGCCTGCACGGCCTCATCTCCAACGGCGGAGGCGGGAGCGGCCTGCGGTTTACGATCCCCAAGGGGTCGCGCACGAAGCCGGTGGGTGCGCCACGTGAGGCCAGGGCAGACAGGGGCGATGAGGACCCTGAGGCGGAGGCCATGGCGGAGGTGGCGTCGGCACTGAGGGCCGTTGGGGAAGGATTTCTGAGGATGGAGGAACGCAGGCTGGAGATGTCGCTACAGATGGAGAAGGAGAGGATGGATTCAGAGATGAAGCGCACCCAGACGCTGCTCGACGCACAGCAGCTCTTCGTCGAGGCCTTCCTCGGCAAGCAGCAGCAGCCGCACCACAAGAAGTCCAAGCTGGCCGCCTTTGCTACGGCTGCTGCCATGGAGGAAGATTGA |
|  | Protein | MSSSSSPKPDAVSSPELPPLAAPNIAAAAAAAAAAASSSGGMGLSGSAVGVGSGRRLPPPCWTHEETLALIEAYRDKWEALRKGNLRAADWDEVAGAVTARCGHFPTATYKSGVQCRHKIEKLRKRYRAERSRSAGRSKGPKWPFFPLLHDLAGGGAPDPSPNPIIKIKPRAGGGTPASPSSPMSSPSSEEAGRSRSLHGLISNGGGGSGLRFTIPKGSRTKPVGAPREARADRGDEDPEAEAMAEVASALRAVGEGFLRMEERRLEMSLQMEKERMDSEMKRTQTLLDAQQLFVEAFLGKQQQPHHKKSKLAAFATAAAMEED |
| BdGT15 | CDS | ATGGATTCCGACGACCCGCGCCACGACCTGCGCGGCCGGGCCCCTGCCCCTGCCCTGCCCGGTGCCAGCGGGGGCGCGACGACGAAGAAGAGCGGGCCGGGGCAGCCCTGGTCGCACGCGGAGACGATGCACCTCCTCGACGTCTACGAGGACCGCTGGAACAAGCTGCGGCGGGGGCAGCTCAAGGCGCAGCAGTGGGAGGACGTGGCGGCCGAGACCACGGCCCGCTGCGTCGCCAGCGGCGCCGGCGCCGGCCAGCGCAAGACGGGCACGCAGTGCCGCCACAAGCTCGAGAAGCTCCGCAAGCGGTACCGCGCCGAGGCCGCCCGCCCCGTCGCCTCGCTCTGGCCCTTCTTCCGCCGCATGGACCAGCTCGAGCGCGGCCCGCGCGCCGCACTCTCCCCGCCCGCCGCCGCGTCCGACGACGAGGGGGAGGAGGAGGAGGAGGAAGAGGAAGAAGGGGAAGAAGATGATGAAGACGAGGAAGGGCAGCGGCAGGTGGAGGAGCCGGAACTCGTCCCGCGGAACAGCAAGAGCATCAACGGCATCCTCCGGGACGGCGGCGGCGGGTTCAGGGGCTTCACCTCCCGCCCGCCGCAGCCACAGCAACCGCCGCCGCCGTCGCCTTGTCTGACGCTGTCCAACGCGCCTCCGCGGAAGCGGGTCGCCTACGAGGCGTTCCAGGCCCAGGCGGCCGCGGACAGGGTGAAGGCAGAGGAGGCTCAGGCCGACCGCTCCGGTGCCGTGGCCCACGGGCCGTTCTCGGACGTGCTGAGGGACTTCGGCGAGGGGCTCATGCGGCTGGAGCGGCGGCGCATGGATGTGCAGTGGGAGATCGAGCGCGGGTGGAAGGAGGTCGAGGCCCGCCACAGCAGGATGCTCCAGGACGCCCAGCGGAGGATTCAGGACACCATAGCCGCCGTCGCCGCGTCGGCCTCGGGGAAGAAGGCCAGGAGGGACCATGGCAGCAGCGGGGACGACGACTTCGCAAGCCAGATGAAGATCCGGCAGCGGTTGGTGGCAAAATTCTTCCAGACGGCGTGGACCAGCGGAAAAATCGGCCGCTCTACGAAGGCAGAGGAGTATGTCGACCTG |
|  | Protein | MDSDDPRHDLRGRAPAPALPGASGGATTKKSGPGQPWSHAETMHLLDVYEDRWNKLRRGQLKAQQWEDVAAETTARCVASGAGAGQRKTGTQCRHKLEKLRKRYRAEAARPVASLWPFFRRMDQLERGPRAALSPPAAASDDEGEEEEEEEEEGEEDDEDEEGQRQVEEPELVPRNSKSINGILRDGGGGFRGFTSRPPQPQQPPPPSPCLTLSNAPPRKRVAYEAFQAQAAADRVKAEEAQADRSGAVAHGPFSDVLRDFGEGLMRLERRRMDVQWEIERGWKEVEARHSRMLQDAQRRIQDTIAAVAASASGKKARRDHGSSGDDDFASQMKIRQRLVAKFFQTAWTSGKIGRSTKAEEYVDL |
| BdGT16 | CDS | ATGTCCAACGCCGGCGACCCCGCCGGCGCCGCGGCAGCGGCGGCGGCCACCCCGTCCGGCCGCGCCCCGAGGCTGCCGCGGTGGACGCGGCAGGAGATACTGGTGCTCATCGAGGGCAAGCGCATGGTGGAGGTCCGCGGGGGCGGGCGAGGCGGGAGGGGCCGCGTGGCGGCGGCTGCGGCTGCGGCGGCCGGGGAGGCGGCGGCGGCGCTGGAGCCCAAGTGGGCTGCGGTCGCCGAGTACTGCCGGCGTCACGGCGTCGAGAGGGGCCCCGTGCAGTGCCGCAAGCGGTGGAGCAACCTCGCCGGGGACTACAAGAAGATCAAGGAGTGGGAGCGGGCGGCCGCCGCCGCGCGGGAGCCCTCGTTCTGGGCCATGCGGAACGACGCGAGGCGGGAGCGCCGGCTCCCCGGGTTCTTCGACCGCGAGGTGTACGACATACTCGAAGGCCGGGGCCGGGGGATCGTCGCCGGGACCTCAGGCGGTAATGCCGCGGGGGAGGAAGAGACGGCGGTGCGCGTGGACGAGGAAGAAGAGGAGGAGGAGGAAGAGAAAGGTAAGGAGAAGGAGGTGGGGGTAGCAGAGGAACCCGTGTTTGACAGTGGCCGCGCGGCCGGGGACGACACGCTGTTCTCGGAGGACGAAGAGGGGGAGCTGGAGGAGACGCCGGCGACGACGCAGGCATCCCCGCCGGCGGTTGTCGCCTTGCCGATATCCGCAGGCACATCATCAAAAGACAAGCAACCAAGGCAGCAAGGCCAAGGCGCTGAAAGAGGAGCATCACCGACGCAACAGCAGCAGCAGCAGCAGAGCGGGCAGAAGAGGCAGCGGACAGGGGACGGCGACAGCGAGGCCGGAGAAGAAGGCACGGCAGGATTGCAGGGCAAGCTACTGGAGATCCTCGACCGGAACAACCGGATGGTGGCGGCGCAGCTGGAGGCGCAGAATGTGAACTGGGAGCGCGACCGGGAGCAGAGGAGAGACCAGGCTGACAGCCTCGCCGTCGTGCTCGGCAGGCTCGCCGACGCCCTCGGCAGGATCGCCGATAAGCTCTAG |
|  | Protein | MSNAGDPAGAAAAAAATPSGRAPRLPRWTRQEILVLIEGKRMVEVRGGGRGGRGRVAAAAAAAAGEAAAALEPKWAAVAEYCRRHGVERGPVQCRKRWSNLAGDYKKIKEWERAAAAAREPSFWAMRNDARRERRLPGFFDREVYDILEGRGRGIVAGTSGGNAAGEEETAVRVDEEEEEEEEEKGKEKEVGVAEEPVFDSGRAAGDDTLFSEDEEGELEETPATTQASPPAVVALPISAGTSSKDKQPRQQGQGAERGASPTQQQQQQQSGQKRQRTGDGDSEAGEEGTAGLQGKLLEILDRNNRMVAAQLEAQNVNWERDREQRRDQADSLAVVLGRLADALGRIADKL |
| BdGT17 | CDS | ATGGTCGCGCTCGCCTCCCTCTCGTCTCTATGCCCCTGCGGCCTCGCTCGCCGACGTTCCGCCTCCGCCTCCGCCTCCACCTCTACCTCCATCTCATGCTGCGCGGTCGCAACTCCTCCCTCCGGCAAAGGGTCTCACGAATCTAGAATACCCCGTAGAAGGTTTAGGAGAACCGAGGGTGCCACTAAGAGCATGGAAGATTCAGTCAAGCGAAAGTTGGAGCAGTTTTACGAAGGGTTGGATGGTCCGCCCCTCCGAGTCCTCCCGATAGGTGGTCTTGGGGAAATCGGCATGAACTGCATGCTCGTGGGGAACTATGACCGCTATATTCTGATTGATGCAGGGGTTATGTTTCCAGACTATGATGAATTTGGGGTGCAAAAGATTATCCCAGACACAACATTTATCAAGAAATGGAGCCACAAAATTGAAGCAGTTATTATAACACATGGCCATGAAGATCACATTGGTGCGTTGCCTTGGGTTATCCCAGCACTTGATTCGAACACACCGATTTTTGCATCGTCTTTTACTATGGAGCTCATAAAGAAGCGCTTGAAGGAGTTTGGGATTTTCCTCTCATCCAGGCTTAAGAGTTTTAGAGTTAGAAATAGATTTCAGGCTGGACCATTTGAAGTGGAACCTATCCGTGTAACTCATTCAATTCCGGACTGCTGTGGCCTAGTGCTTCGCTGTGGTGATGGTACAATTTTCCATACCGGTGACTGGAAAATCGATGAATCACCAGTAGATGGAAAAATATTTGATCGAGAAGCATTGGAGGAGCTCTCCAAAGAAGGCGTTACTCTGATGATGAGCGACTCAACGAATATCCTATCCCCTGGAAGATCCACCAGCGAATCTGTTGTTGCTAGTTCGTTGTTGCGTCATATTTCAGAAGCAAAAGGAAGAGTAATTACAACACAATTTGCTTCAAATATACATCGTATTGGGAGCATCAAAGCTGCTGCAGACTTAACTGGTCGAAGACTGGCATTTGTTGGAATGTCACTCAGGACATACCTTGAGGCTGCTTTCAGGGATGGAAAGGCACCGCTAGATCCATCAACCTTGGTTAAGGCAGAGGACATGGATGCATATGACCCTAAAAGTCTTTTAGTCGTTACTACAGGTTCACAAGCAGAGCCACGTGCAGCTCTGAATCTTGCTTCTTATGGGGGAAGTCATGCTCTTAAACTATCCAAAGAGGACGTCCTTTTGTATTCAGCTAAGGTTATTCCTGGAAATGAGTCTCGGGTAATGAAAATGTTAAATCGCCTGACTGATCTTGGCCCAAAAATTATTATGGGTAAAGACTCTGGCCTCCATACCTCTGGGCATGCCTATCATGATGAGCTGGAAGAAGTTCTACAAATCGTTAAACCGCAGCATTTCTTGCCTGTTCATGGGGAACTCCTGTTTCTCAAAGAACATGAATTGCTTGGAAGATCAACTGGCATACGACATACTACTGTAATTAAAAATGGGGAGATGCTCGGTGTGTCTCATCTAAGAAACAGAAAGGTTTTGTCCAATGGGTTTGTTGCATTAGGAAAGCAAGAATTTAAGCTAATGTATAGTGATGGCGATAAGGCTTTTGGAACGTCCACTGATCTCTGCATTGATGAGAGATTAAGAATTGCATCTGATGGCATTATTTTCGTCAGCATGGAGATCTTCCGACCTCAGAAGGAACTTGCTTCACCACAGTCAGGCTTGAAAGGGAAATTTAAAATAACAACACGGTGTCTATGGCTTGATAATGGAAGACTACTCGATGCACTTTACAAGGCTGCACATGCTGCACTATCAAGTTGTCCTTTGAATTGCCCACTTAGTCACATGGAGAGGATGGTATCTGAAATCTTAAGGAAAATGGTAAGGAAGTACAGTGGGAAGAGGCCTGACGTCATTGTGGTTGCTTCCGAGAACACCACGATAGGTTTCACAGAAGAGGTAACAAATAAATCACCCGGGAAGTTTGGGCCTTTCTCAGCTACTAGGCATCTTAGCAGGAGTCCAGATAGGAGTCTTGAAGATAGTGATAAAACACGGCTAGATAACCCGGAGGGGGAAGCTGCAGATAGCACTCCTGAGGTCATGAGAACAACATCTGACGATGTAACCACTAGCACCAATGGTGAATCATTCTTCTCTTCAGATTTGCATCAGCCTAAAGCACTGGATCATTTTTGGGAGTCATTCAAATCCCCTACTGCAGTAAAAATCGCAAGAATTGTCAATGCCTCAGCTCAAGGGAACAAACCAAAGCTCGGCAAGATCAGCATAATTGATAAGGACTCCAGCATGTCGGTTCCTGCTCCAGCTAAATCTCCGAGAAAAAACAAGTGGAAGCCTGAGGAAATTAAGAGCTTAATACAGCTGCGTGGTGAAATGAATGAGAAATTCCAAACAGTGAAAGGAAGGATGGTTCTGTGGGAAGAGATATCTGCCAGCATGTTGAACCAGGGGATAACTCGTACACCGGCGCAGTGCAAATCTCTCTGGACATCATTGGTTCAAAAATATGAGGAGAGCAAGAAGGATGGAGAAGGCATGAAGACATGGCCGTATTTCTCCGCTATGGATAGTTTTCTGTCATGCGAAGGGGAAATGGCAACCAAATGA |
|  | Protein | MVALASLSSLCPCGLARRRSASASASTSTSISCCAVATPPSGKGSHESRIPRRRFRRTEGATKSMEDSVKRKLEQFYEGLDGPPLRVLPIGGLGEIGMNCMLVGNYDRYILIDAGVMFPDYDEFGVQKIIPDTTFIKKWSHKIEAVIITHGHEDHIGALPWVIPALDSNTPIFASSFTMELIKKRLKEFGIFLSSRLKSFRVRNRFQAGPFEVEPIRVTHSIPDCCGLVLRCGDGTIFHTGDWKIDESPVDGKIFDREALEELSKEGVTLMMSDSTNILSPGRSTSESVVASSLLRHISEAKGRVITTQFASNIHRIGSIKAAADLTGRRLAFVGMSLRTYLEAAFRDGKAPLDPSTLVKAEDMDAYDPKSLLVVTTGSQAEPRAALNLASYGGSHALKLSKEDVLLYSAKVIPGNESRVMKMLNRLTDLGPKIIMGKDSGLHTSGHAYHDELEEVLQIVKPQHFLPVHGELLFLKEHELLGRSTGIRHTTVIKNGEMLGVSHLRNRKVLSNGFVALGKQEFKLMYSDGDKAFGTSTDLCIDERLRIASDGIIFVSMEIFRPQKELASPQSGLKGKFKITTRCLWLDNGRLLDALYKAAHAALSSCPLNCPLSHMERMVSEILRKMVRKYSGKRPDVIVVASENTTIGFTEEVTNKSPGKFGPFSATRHLSRSPDRSLEDSDKTRLDNPEGEAADSTPEVMRTTSDDVTTSTNGESFFSSDLHQPKALDHFWESFKSPTAVKIARIVNASAQGNKPKLGKISIIDKDSSMSVPAPAKSPRKNKWKPEEIKSLIQLRGEMNEKFQTVKGRMVLWEEISASMLNQGITRTPAQCKSLWTSLVQKYEESKKDGEGMKTWPYFSAMDSFLSCEGEMATK |
| BdGT18 | CDS | ATGGAGGGGAATTTGCCACCAAGAACATTGATGCCTGGAGCCCTTTCTGATGTGGAGGCGTCGTTCGAACCGCACCAGCAGCAATTTCAGTTCGCCCAGCCCCAGCTTCATCAGGGTATGTTTGCAACCCCTGCGAATCCGGTGCAGGAATTAAGCAATGGGGTGAAGACCTCGCAGAGCGACGAGGACGATGCCCTCCACCATGACCGCGGTGGCAAGGCGGCCTCCCAGTGGCACCGGGTGAAGTGGACCAGCGACATGGTCAAGCTGCTAGTTTCGGCCGTGGCGTACATCGATGAGGATGTCGAGATGGACTATGGTTCGAGCAGGAAGAAGAAGCACGCCATGTTGAAGAGGAAGGGCAAGTGGAAGCTGGTCTCCATGTCAATGAACGAAATGGGGTTCGCGGTGTCGCCACAGCAGTGTGAGGATAAGTTCAATGACCTGAACAAGAGGTACAAGAGGCTGACTGAAATTCTTGGCCGGGGAACAACTTGCCAGATCGTCGAGAACCCTCAGCTTCTTGAAGGGGTCAGCCTCTCAGGAAAGCTCAAGGAGGAAGCAAGGAAGCTTCTGAGCTCAAAGCATCTGCACTATGAGGAGATGTGCTCCTACCACAACCAGAACAGGCTGTGTCTGCTTGAAGATCCGGTCCTTCAGAAGTCACTGCGTTTAGCGCTCAGGAGTAAAGATGATCATGAAAAGAAGAGCTCTATTGGTGATGATGATGAGGATGATGAGATGATGCTTTCTGATGACGATGAAGAAGACGACGAATTCAATGATGACACAGAGGCAAGTGCTGAAGATCATCATCACAGAAGTCACGGCACAGAGAGGCTGCAGCTTGATTATGGCGAGACATGGTCTGGATCACATCTGTCAGAAGTTGCTGCTATTGACATGAACAAAATGCTCTGCGAAAGATCTGGCGGTCTATCTGCTGAGAAGGATTCCCCTGGTACACATGAGATTCAAATTTTGAAACAGCATCTGAAGTTAAAAGCTCAAATGCTGAAATTTAAGCAGCGACAATTCAAGTGGCTGAGGTTCAGCAAGGAGAAGGACAGGGAACTGGAGAAGATGAGGCGGGAAAACGAGAAGATGAGACTGGAGAATGAGCGGTTGGAGCTGGAGCTGACGCTTAAAGGATTAGAGATGGGTATCAAACCAAAGATAGTTATTGGAGACTGGAAATGGTAA |
|  | Protein | MEGNLPPRTLMPGALSDVEASFEPHQQQFQFAQPQLHQGMFATPANPVQELSNGVKTSQSDEDDALHHDRGGKAASQWHRVKWTSDMVKLLVSAVAYIDEDVEMDYGSSRKKKHAMLKRKGKWKLVSMSMNEMGFAVSPQQCEDKFNDLNKRYKRLTEILGRGTTCQIVENPQLLEGVSLSGKLKEEARKLLSSKHLHYEEMCSYHNQNRLCLLEDPVLQKSLRLALRSKDDHEKKSSIGDDDEDDEMMLSDDDEEDDEFNDDTEASAEDHHHRSHGTERLQLDYGETWSGSHLSEVAAIDMNKMLCERSGGLSAEKDSPGTHEIQILKQHLKLKAQMLKFKQRQFKWLRFSKEKDRELEKMRRENEKMRLENERLELELTLKGLEMGIKPKIVIGDWKW |
| BdGT19 | CDS | ATGGACGACGACGGCGCCGCCTCCCCGTCGCCATCGCCCTCCCCATCGCGCTCCTCGTCTCCGCTCCCGCTCGCCGAACCCGTCACCGTCGCCGCCGTGCCCCCCGGCCACCTCGCCGTCGCCCTCCCCATCCAGAAGCCTGCCCCCTCCAGCGGCGGCGGCGGCGGCGGAGGGGGCCGAGAGGACGCCTGGAGCGACGGCGCCACCTCCACGCTGATCGACGCCTGGGGCGAGCGCTTCGTCGCGCTCGGTCGCGGCAGCCTACGCCACCCACAGTGGCAGGAAGTTGCCGAGGTCGTCTCCTCCCGCGACGGCTACTCCAAGCCGCCCAAGTCCGACATCCAGTGCAAGAACCGCATCGACACCCTCAAGAAGAAGTACAAGGTCGAGAAGTCCAAGTCGGGCTCCTCATGGCCATTCTTCGACCGCCTCGACTACCTCCTCGCCCCCGTCCAGAAGCTCGGGGGCAATTCTGGCCGGGCTGCAGGAAATTCGGGCTCCTCTAACCTCGCCGAGCGGACAACCGCTGTTATGATTCCGCGTTTCAACTTCCCTACGCGCAGCCGTCTAAAGCGCAGGCTGCCGTCGCCGCCCCAGGCCTCAGCATCGTCGGATTCGTCCGATGGGTTCCCACCTGAGCCACCGGTTGAGGCTGCCAACGGCAAGAGACAGCGTGTCGAGGAGGCTGTGAATGGAGCAGATAGCAGCGTCCGTGTGCAGGGTCTGCGCGACCTGGCGCAGGCGATTCGGCGGTTTGGTGAGGCGTTTGAACGTGTTGAGGCCTCGAAAAGGGAGCATGAGCTCCGGATGGAGCGGGACCGCCTGAATGCTGCCCGCGAGCTAGAGGACCAGCGTGTGCAGTTCTTCCTCAAGATGCAGATGGAGATCTCAAAGGCCAACAATGGTACCGCACTTGGTCCCCTTGCTGCCGTTGCTGCTGCCATGATTGGCAATTCAGCACCTGCCACAGTCCCCGCCGTTGCTGATGGCAACGGCTCCAGGAGAACATCTATGGCACCTGATGTCGGGACCAGCAGCAATCACCATGTCCGGTACCGTGTGAAGGATGGCAGGCATCATCAATCCTTGCAACACCCATCCTACCAGTACAACCAGAACAATGCCGGCGAGGATGCCACAGGCCATGGTAGTGGCAGCAACGACAACAAAGAAGATGATGAGGTGGAAGATGAAGAGGAAGAGAGCCAGTAG |
|  | Protein | MDDDGAASPSPSPSPSRSSSPLPLAEPVTVAAVPPGHLAVALPIQKPAPSSGGGGGGGGREDAWSDGATSTLIDAWGERFVALGRGSLRHPQWQEVAEVVSSRDGYSKPPKSDIQCKNRIDTLKKKYKVEKSKSGSSWPFFDRLDYLLAPVQKLGGNSGRAAGNSGSSNLAERTTAVMIPRFNFPTRSRLKRRLPSPPQASASSDSSDGFPPEPPVEAANGKRQRVEEAVNGADSSVRVQGLRDLAQAIRRFGEAFERVEASKREHELRMERDRLNAARELEDQRVQFFLKMQMEISKANNGTALGPLAAVAAAMIGNSAPATVPAVADGNGSRRTSMAPDVGTSSNHHVRYRVKDGRHHQSLQHPSYQYNQNNAGEDATGHGSGSNDNKEDDEVEDEEEESQ |
| BdGT20 | CDS | ATGGCCGGCAGCGTCCCTTCTCCGCAGCCGCCTCCGGTGCCTGCTGCCGTGTCTCTCAGCCTTGTTCCGTCTCCTCCGTCTTCTGTGCAGACGCCACCGTCGGTGACGAATTCTGAGGTTTTGCCAGCCGCTGGCACTGGAGCTAGCACCGGCCATGGCGTCATCAACTTGAACGACATCAATCTCGTGGACGGCCTCCGTGGCAGAAGCACCGGCGAGGCTGGAGCAGGAGGGTCTGGCTGCAGCCGCTGGCCACACGATGAGACGCTGGCGCTTATCGAGATCCGGTCGGAAATGGACAGCGTCTTCCGCAACGCCGGCCTCAAAGCGCCCGTTTGGGAGGAGGTCTCCAGGAAGCTAGCGCAGCGAGGCTACCGCAGGAGCGCCAAGAAGTGCAAGGAGAAGTTCGAAAACGTTGACAAGTACTACAAGCGCTCCAAGGAAGATCGTGCCGGCAGGCCGGACAACAAGAGCTACCGCTACTTCGCCCAACTCGAAGCACTCCACGCTGCAGACCCGCCGCAGCACCAGGCCGCGCCAATGGCCATGGCGGCTGCAGCTGCGAGACCGTTGGCGATGGCCCATCCTGGCGCTGGCCTGCTGCTGCCGGACCACGGCTTCTGGTCCAATACGGAGCCAGACTCAGACAATGAGTCCGATGACGAGGAGGAAGGGGGCGGTGGTATCAGTTACAAGATGTTGACGGTGCTCGTGAAGCGTCTGATAAAGCAGGTCACGAAGAGGCAAGACGCAATGCAGCAACTACTCCTTGATAAGCTCGAGAGGTGGGAAGCAGAGCGCACTAAGCGAGAGCAGGCATGGCGGGCGCATGAACTCACCCGCATAAAGTGCGAATGGGAGCAACTCGCCCAGGAGCGAGCTACCGGAGCCACCCGCGACGCTGCCCTGATCATGTTACTCCAGCGCATCATCACCAGCGGGAACTCCGTATCGCTCCCTCCTTCCTTCAGCTCCTTAGCTGCGGCCAACAAACAAAAGGCGCCAGTGATACAGAATACCTCTCAGATACATCATCACATTGAAGCATTAATCGCCTCAATGCAGTCAGTGGTTGCGAGCCGGTCAGTAGGAGAGAGCAGCGAGGGGGATAATAATGGGAGCGGTATGCCACGATGGCCCAAGGAGGAAGTCGACGCGCTAATACAATTGCGGATGGAGAAGGACGAGCTTTGGCAGGATTTGGGAGGCAAGGCTCTACTGTGGGATGACATTTCTGCAGCCATGCGGAACATGGGGTACCACCGGAGCTCCAAGCGGTGCAAGGAGAAGTGGGAGAACATCAACAAGTACTTCAAGAAGGACGCCAAGACGTGCCCTTACTTCCACCAACTTGAGGATATCTACCGCAAGAGGCAGCAATTCACCATGACCGGCAACGGCGGCAGCTCGAGCACTGCCCCCGATGACACTACGGTTACCGTCACCTTCTCGGAGCAGCCTGTCTTGGAGCAGAACCACCAATGTGATCTTCAAGGTAACATCGGCAATAACTTTGGTATGATGGTTAACGACGGTGGGGAGAACTCACATGCCTCACCATGCAACGACGGCCACACGGCAATGCACACAACCGTGGTTGATGACATTGCTATAAACGAGGAGGAGAACACCCATCGCGAGCTGGAAGATAAGATCAACAATGACTTGGGCATGGTGGAAAACATTGAAGGGGGAAACTTGCATACCACACTGAGCAACGACGGCGATACGGCAATGGTCACTGTCGTGCTTGATGATAGCTCTATAAACAAGGAGGAGAATCCCCATCGTGAGCTCAGAGTTAGGATCAGCAATAAATTCAACATGATGGGTAATGACGGAGGGGAGAACTTGCTTGCCCCATCGAGAAACGGTGGGGATACACCAATGGACAACAACATGCTTGATGACGACGCTAAAAACAAGGAGAACCCCTCGCGTGAGCTCGAAGTTAAAATTAGCAAAGATTTCAGCATGGTAGATAATGGCGGAGGCGAGAACTTGCATGCCCCACGGAGCAAAGACGGCGCTACAACAGTGGTTACAGCCGTGCTTGATGAAAGCGCAATAAACATTGAGGAGAACTTGCTTGCCCCACCCTGCAATGGCGGCGATATGGCAATTGACACGGGCGTGATTGATGGTAGCTCTACAAATAAGGAGAACCGCTCACGCGAGCTCGAAAGTAAAATTAGCAATGACTTCATCGCGATGGATAATGTTGGAGGGGAGAATTTGCTTTCCCCACCGAGCAACAACGATGATACGGTAGTGGATACATCCGTGCTTGATGAAAGCGCAATAAACAAGGATAAACCCTCATGCGCGATCGAAGGTAAGATTAACAATGACTTCGCCATGAGGGATAACATCGGAGGGGAGAACTTGATTGCCCCGAGCAACAACGGCGATACGATTGTGGATACAACCTTGCTTCATGACAGTGTTGTAAACAAGGAGGAGAACCCTTTGTACGAGCACGAAGTTAATATCAACAATCATTTCAGCATGATGGGTAACGGCGGAGTGGAGAACTTGCTAGCCCCACCCAACAACAATGGCGAGATGGCAACAGACACAGTCGTGCTTGATGACAATGCTATAAACAAGGAGGAGAATCCCTCACAGGAGCTCGAAGGTACGATTAGCAATGACTTTGCCATGATTATTAATGGCGGAGGAGAGAACTTGCTTTCCCCACTGAGAAATGATGGTGATACGACAGTGGATACAACCGTACTTGATGGCAGCATTGTAAACAAGGAGGAGAACCCTGGGCACGAACACGAAGTTAATATCAGCAATCATTTCGGCATGATGGGTAGCAGCGGAAGGGAGAACTTGCTGGTCCCACCCAGCAACAATGGTGAGATGGCAACGGATACAGTCGTCCTTGATGACAATGCTATAAACAAGGAGGAGAATCCTTTGCGTGAGCTCAAAGGTAACATTAGCAATGACTTTGCCATGATGATTAACGGCAGAGGGGAGAACTTGCTTTCCCCACCGAGAAACGATGATGACACGACAGTGGATACAGCCGTGCTTGACGAGAGCGCAATAAAAAAGGAGGAGGACCCCTGGCGCAATCTCGAAGGTAATATCAACAATGACTTCAACATGATAGGTAATGATGGAGAGGAAAACTTGCTTGCTGCACCCTGTAATGGCGTGGATACAGCAATGGACGCAACAGTGCTTTATGACAACGCTATAAACCATGAGAAGAACCCATTGCGCGAGCTTGAAGATAAGACCATTAATGAATTTGCCATGATGGAAAATAGTGGAGAGGAGAAATTGCTTGCCTCATCAAACAACGGCGGCGATACGGCAATGGACACAACCATGCTTGATGACAGCACTATAAACAAGGAGGAGAACCCCCGGAGAGAGCTGAAAGGTAATGTCAGCAATGACTTCGACATGATGGGTAGCGACGAAGGGGAGAACTTGCTTGCCCCACCGAGCAATAGTGATGATATTGCAATGGACACCTCCGTACTTGATGACATCCCTATAAACAAGGAGAACCCCTTGCACGAGCTCGGAGGTAATATCAACAATGAATTTGTCATGATGGGTAACGATGGAGGGGAGAACTTGTTTGCCCCACCCAGCCACAACTGTGATACAACAATGGACACAGCCGTGCTTGATGACAGCGCTATAAACAAGGAGGAGAACCCCTGGCGTGAGCTCGAAGGTAACATCCGCAGCGACTTCAGCATGATGGATAACGGCGGAGGGGAGAACTTGCTTGCCCCACCCAACAACATCGGCGATAAGGCAATGGACACAACCGTGCTTGATGATAGCGCTATAAACAAGGAGAACCCCTGGCGCGAGCTCGAAAGTAAGATCAACAATGACTTCAAGATGATGGATAACGGCGGATGGGAGAACTTGCTTGCCCCACCTAGCAACAACGACGATACTACAATGGACACAGGTGTGCTTGATGACAACCCTGTAAACAAGAAGAAAGAAGACATTGTGATGACGACTGAGGCAAACATGCAGCCTCAGCAGAAGGACTTTACAGATTGGGAGGAGACGTGTAGCGACGACATCGAAGGCAACTACACCCATGAAGATCGTGACAATGGTGGTGGCAGCAGCGACAATGAGAATGATAAAATGAAATGCATCGTGGAGCTCCGGAAGCAACAATAG |
|  | Protein | MAGSVPSPQPPPVPAAVSLSLVPSPPSSVQTPPSVTNSEVLPAAGTGASTGHGVINLNDINLVDGLRGRSTGEAGAGGSGCSRWPHDETLALIEIRSEMDSVFRNAGLKAPVWEEVSRKLAQRGYRRSAKKCKEKFENVDKYYKRSKEDRAGRPDNKSYRYFAQLEALHAADPPQHQAAPMAMAAAAARPLAMAHPGAGLLLPDHGFWSNTEPDSDNESDDEEEGGGGISYKMLTVLVKRLIKQVTKRQDAMQQLLLDKLERWEAERTKREQAWRAHELTRIKCEWEQLAQERATGATRDAALIMLLQRIITSGNSVSLPPSFSSLAAANKQKAPVIQNTSQIHHHIEALIASMQSVVASRSVGESSEGDNNGSGMPRWPKEEVDALIQLRMEKDELWQDLGGKALLWDDISAAMRNMGYHRSSKRCKEKWENINKYFKKDAKTCPYFHQLEDIYRKRQQFTMTGNGGSSSTAPDDTTVTVTFSEQPVLEQNHQCDLQGNIGNNFGMMVNDGGENSHASPCNDGHTAMHTTVVDDIAINEEENTHRELEDKINNDLGMVENIEGGNLHTTLSNDGDTAMVTVVLDDSSINKEENPHRELRVRISNKFNMMGNDGGENLLAPSRNGGDTPMDNNMLDDDAKNKENPSRELEVKISKDFSMVDNGGGENLHAPRSKDGATTVVTAVLDESAINIEENLLAPPCNGGDMAIDTGVIDGSSTNKENRSRELESKISNDFIAMDNVGGENLLSPPSNNDDTVVDTSVLDESAINKDKPSCAIEGKINNDFAMRDNIGGENLIAPSNNGDTIVDTTLLHDSVVNKEENPLYEHEVNINNHFSMMGNGGVENLLAPPNNNGEMATDTVVLDDNAINKEENPSQELEGTISNDFAMIINGGGENLLSPLRNDGDTTVDTTVLDGSIVNKEENPGHEHEVNISNHFGMMGSSGRENLLVPPSNNGEMATDTVVLDDNAINKEENPLRELKGNISNDFAMMINGRGENLLSPPRNDDDTTVDTAVLDESAIKKEEDPWRNLEGNINNDFNMIGNDGEENLLAAPCNGVDTAMDATVLYDNAINHEKNPLRELEDKTINEFAMMENSGEEKLLASSNNGGDTAMDTTMLDDSTINKEENPRRELKGNVSNDFDMMGSDEGENLLAPPSNSDDIAMDTSVLDDIPINKENPLHELGGNINNEFVMMGNDGGENLFAPPSHNCDTTMDTAVLDDSAINKEENPWRELEGNIRSDFSMMDNGGGENLLAPPNNIGDKAMDTTVLDDSAINKENPWRELESKINNDFKMMDNGGWENLLAPPSNNDDTTMDTGVLDDNPVNKKKEDIVMTTEANMQPQQKDFTDWEETCSDDIEGNYTHEDRDNGGGSSDNENDKMKCIVELRKQQ |
| BdGT21 | CDS | ATGGAAGGCAGCAGCCTACCCCCTGGAAACCCGATGCAAGTAACTCCTTATGGTAGTTTGAATGGACATGGCAACTCAATGCAAATGCATGCTCCATGCTCAGGAAAGCAGCCATTTAACCAATCTCTGATGCTAGGGAGCTTCGCAATGCCTGTCAACCGGATTCAAGACCCTGACAGATTACCCAGATTTCAATTCGGAGAACATGGAAAAGTTGACCACCACCAGCACAGTCATCGCTCAAAGAACTGCCTCAGTGATGAGGAGGAGCATGATATGACCGAGGATGTGGGTGGCAAAGGAAAGAATGGCTCCGCATGGCATCGGGTGAAGTGGGCAGGTTCAATGGTTAAGCTTTTGATTACTGCAGTATCTTACACCGCGGAGGATCCTGGGCCTGATCTGAACTGCGGGAAGAGGAGTGGTACAATAGCACAGAAGAAGGGCAAATGGAAAGCAATATCAAAGGTCATGTGTGAAAGAGGCTGCAATGTGTCACCGCAGCAGTGTGAGGATAAGTTCAATGACCTGAACAAGAGATACAAAAGGCTTACAGATATCCTTGGTTGCGGTATTGCTTGCGATGTTGTGGGCAATCCGGCACTTCTTGATGGCATGGATAATCTTTCTGATAAGATGAAAGAAGACGCAAGGAAGATACTGAGCTCTAAGCACTTGTTCTATGAGGAGATGTGTTCTTATCATAACAACAACCGTGTGAGTTTACCAGAAGATCTCGAGTTTCAGCACTCACTACAGCTTGCTCTTAGATGTAAAGAGGACCGTGGTCCGAGGAAGGATACCAGTGGAGAGGATCAAAGTGCAGATTCTGATTACGAGGAGAATGATGAATGGCATCAAGCGGTCCGTACCAACATGAATAAAAGGATGTGCCATACGGTGGATCATGTTGATGTGGGTTTCGTCCCTTCAAGCTCGAACAATGGCAGTGGGAGGTTCGATCCCCATGGCATCACATTGGACATCAACAAAGGTTTACCAGATGGAACCAACTTGCCTTCTCTGCAAAAGGACTTGGCTTCACAAGCACTGGAGCTTCAGGAACGTCGACTGCAGATTGAAGTAAACGAACTGGCAATAACAAAGCGACGTCTCAAGTGGGAGAGATTCAAGAAGCAGACGGAGAGGGAAATGAAAAGGATGGAACTGGAGAACGAACGGATGATGATCATGAACAAGCGGTCACAACTTCTGCTAAGACACAAGGAGCTCGAGCTCATGGCAAAAGGCGATCCCAATCATGCATGA |
|  | Protein | MEGSSLPPGNPMQVTPYGSLNGHGNSMQMHAPCSGKQPFNQSLMLGSFAMPVNRIQDPDRLPRFQFGEHGKVDHHQHSHRSKNCLSDEEEHDMTEDVGGKGKNGSAWHRVKWAGSMVKLLITAVSYTAEDPGPDLNCGKRSGTIAQKKGKWKAISKVMCERGCNVSPQQCEDKFNDLNKRYKRLTDILGCGIACDVVGNPALLDGMDNLSDKMKEDARKILSSKHLFYEEMCSYHNNNRVSLPEDLEFQHSLQLALRCKEDRGPRKDTSGEDQSADSDYEENDEWHQAVRTNMNKRMCHTVDHVDVGFVPSSSNNGSGRFDPHGITLDINKGLPDGTNLPSLQKDLASQALELQERRLQIEVNELAITKRRLKWERFKKQTEREMKRMELENERMMIMNKRSQLLLRHKELELMAKGDPNHA |
| BdGT22 | CDS | ATGGATGGCCTTACAGACGCCGACGTCGCGCCTCTGGCGGCGGCGGCGGCGGCGGCCCCGCAGCAGCAGCAGCAGCAGAAGCGGGACGAGTGGAGCGAGAGCGGCATCTCGCGGCTGCTCGAGGCCTACGAGGCCAAGTGGCTGCTCCGGAACCGCGCCAAGCTCAAGTGGAGCGACTGGGTCGACATTGCCAACGATGTGTCGGTGCACTGCTCCTCCGAGAGCGCCGCCGCGGGGAAGCCCGGCGGCAGCAGCGCCAAGACCCCCAACCAGTGCAAGAACAAGGTGGAGTCCATGAAGAAGCGGTACCGAGCTGAGTCCGCCGCTGCGGCGCGCTCTGGCCCTGGCGCCGCTGCCAATGCCGCCGGCCCGTCGTGGCGGTTCTTTGGCCGCATGGATGGACTGCTCAAAGGGCCGGGCGCTGGTTCCTCCGGTCAGGTGCAGGCTGAGTTAAGCAACGGTGTAGACCTTCGAGTCCCCCCAGCGAAAGCAGAGCCGGAGGTTGAGGTGGAAGGTGGTCTTCTCCGGCAGCTGCCAGATGCAGGGCCAAGTGCACTTTCCGAGCTGGTGAATGCAGATGGAAATGGTTTTGCGACAGAGAGAGCCGAGAAGGTTGATTGCAGCATGCAGAAAGATAGCAGAGCTGTAGATAGTGATGCCAATGTGAGCTCCCCGAGGAGCAAGGAGGCGAAAGAGGACGTCGAGGAGGTGGGCAAGGTCTGGGACATGCCTAAGAAGAGGAAGAGTTCAGAATTTGATATAGGGAAGAGCATTGAGTTGCTGGCTAGCTCATTCCTGAAGATTGAGCAGGCCAGGATGGAGATGTACAGGGAGACTGAGAAGATGAGGGTTGAGGCTGAGATTAAGAAGGGCGAGATGGAGCTCAAGAGAACGGAGATCATGGCGAAAACACAACTGCAGATTGCTAAACTTTTTGCCAAGAGATTGAAGGAGTGCAGCAGGACTGGTGGTAGCTCGTCAGTAGCAGCTGATGTGGATACCCTTACCAAGAAAGGCGAAAATGGTTCAGGGTGA |
|  | Protein | MDGLTDADVAPLAAAAAAAPQQQQQQKRDEWSESGISRLLEAYEAKWLLRNRAKLKWSDWVDIANDVSVHCSSESAAAGKPGGSSAKTPNQCKNKVESMKKRYRAESAAAARSGPGAAANAAGPSWRFFGRMDGLLKGPGAGSSGQVQAELSNGVDLRVPPAKAEPEVEVEGGLLRQLPDAGPSALSELVNADGNGFATERAEKVDCSMQKDSRAVDSDANVSSPRSKEAKEDVEEVGKVWDMPKKRKSSEFDIGKSIELLASSFLKIEQARMEMYRETEKMRVEAEIKKGEMELKRTEIMAKTQLQIAKLFAKRLKECSRTGGSSSVAADVDTLTKKGENGSG |
| BdGT23 | CDS | ATGGAAGGGAATAACCTACCCTCTGGAAACTTTATGCAAGAGACAGTTCGTAAACCAATGCAAATGCGTTCTCTAAACTCTGGAAATCAGGGCTTCAACTCTCAGATACCTGTTTTGTTCCCCATGCCTATGAACCAGGTCACAGACTCCGATCACTTGTCAGAACTTCAGTTTGGAGAACGCGGGAAGAGTGATCACCATCACCACCATCAAACGAAGGACTCCATGAGTGATGATGAGGAGCATGATCTGAATGAGGATGCCACTGATGGCCACATTGGCAAGGGAAAGAAGGGATCTGCATGGCAGCGGATGAAGTGGACTGATTTAATGGTTAAGCTTTTAATTACTGCAGCGTCTTACACCGGGGAGGATCCAGGAGCTGATTTAGGAGGTGGGAGGAAGAGCTGTGCAATGATGCAGAAGAAAGGCAAGTGGAAGGCAATATCAAAGGTCATGGGCGAGCGAGGCTGCAATGTGTCACCACAGCAGTGCGAGGATAAGTTCAATGACCTCAATAAGAGATACAAAAGGCTTACGGATATCCTTGGTCGGGGCACAACTTGCAGGGTAGTGGCAAATCCAGCACTTCTGGATCACATGGATAATCTGTCTGACAAGTTGAAAGATGATGCAAGGAAGATATTAAGCTCAAGACACTTGTTCTATGAGGAGATGTGCTCCTACCATAATAACAACAGGGTTAGTTTGCCTGAAGATCCTGCACTGCAGCGTTCGCTGCAGCTCGCTCTTAGGTGTAAGGAAGAAAATGATCTGAAGAGAGGAGTAAGTGGAGACGCTGATGAAGATGACCAAACTGCAGAGTCTGATTCTGAAGAGGATAATGATGAGGAGCATCATCCGACACATAGCAGCAAGGGGGCCCTACCCATGCATAAAAGGATGCGGCATATGACGGATCATGAGGATGTAGGTTTTGGCAACTCTTCTAGCTCACATGACTGTAGCGAGAGGACTAATCTGCACGGCATTGCACTAGATATCAACAAAGTTTTTCTAGATGGAACTAGCTCGGCTTTGGCACAAAAAGACTTGGCATTACAATTAGAACAGCTTGAGAAATATCGCTTCCAAATTGAAATTCAGGAACTGGAGCTTGCAGAACAACGCTTCAAATGGGAGCAATTCTGTAAAAAGAAGGACAGGGAACTTGAAAGGATGGAATTGGAGAGTGAACAGATGAGGGTTGAGAATAAGCGCCTGGCGCTGGAGGTAAGGCGCAAAGAGTTAGAGTTTGAACTCAAGGTGAAAGGAAATGACAATCATTGA |
|  | Protein | MEGNNLPSGNFMQETVRKPMQMRSLNSGNQGFNSQIPVLFPMPMNQVTDSDHLSELQFGERGKSDHHHHHQTKDSMSDDEEHDLNEDATDGHIGKGKKGSAWQRMKWTDLMVKLLITAASYTGEDPGADLGGGRKSCAMMQKKGKWKAISKVMGERGCNVSPQQCEDKFNDLNKRYKRLTDILGRGTTCRVVANPALLDHMDNLSDKLKDDARKILSSRHLFYEEMCSYHNNNRVSLPEDPALQRSLQLALRCKEENDLKRGVSGDADEDDQTAESDSEEDNDEEHHPTHSSKGALPMHKRMRHMTDHEDVGFGNSSSSHDCSERTNLHGIALDINKVFLDGTSSALAQKDLALQLEQLEKYRFQIEIQELELAEQRFKWEQFCKKKDRELERMELESEQMRVENKRLALEVRRKELEFELKVKGNDNH |
| BdGT24 | CDS | ATGTCTGGCGCGGGCGAGGGAAGCGGCGGGGGGCGCGCGGCGCGGCTGCCGCGGTGGACGCGGCAGGAGATACTGGTGCTGATCGAGGGGAAGCGGGTGGTGGAGGCGCGCGGGCGGGGCCGCGGGGGCAGGGGCGCGGGAGGGCTAGCGGGGGCGGAGCCGACCAAGTGGGCGGCGGTGGCGGAGTACTGCCGGCGGCACGGCGTGGAGCGTGGCCCCGTGCAGTGCCGGAAGCGGTGGAGCAACCTGGCGGGGGACTACAAGAAGATCAGGGAGTGGGAGCGCTCCCTGCTGCTGCCGTCCGGGGGCAAGGAGGTGTCCTTCTGGGCCATGCGCAACGACGCGCGCAGGGAGAGGCGGCTCCCGGGCTTCTTCGACCGCGAGGTCTACGACATCATCGAGGGCCGCGGCGGCTCCCGGAATGCCGCGGCCCAGGCCCAGGTGGTGGTGGTGGAAGAGGAGGGGAAGGAGAAGCCGGCCCAGGCCTTGAACAGCGGCCGTGGTGTCCCTGAGGAGGGGCTTTTCACGTCCTCGTCCGCCTCGTCTGAGGATGACGACGACGAAGCATCCGTCGCGCCGCCGGCTCCGGTTCTTCCGTCTCCGTCTCCTTCTCCGGCCCCTGCCCACGCGGTGGTAGCCGTGACGGTACCCGACAAGAAGAACGAGGCACCTGTGCAAGATAGCTCAGAGAAAGCTACATCCAAAGGCAAGCAACAAGAACAAATTATGGACGATCCACCACCAGAGCAAGGCGGGCAGAAGAGGCAGCGCAGCGATGACACCACAGGAGAAGCAACTGACCTGCAGGGCCAACTTATTGAGATTCTAGATCAGAGCAGTCGGATGGTGGCGGCGCAGCTGGAGGCTCAGAACATCAACTGCCAACTGGATAGAGAACAGAGGAAAGACCAAGTGAGTAGCTTGCTCAGTGTGCTTGGCAAGGTGGCCGATGCCCTCTACAGAATCGCTGATAAGCTGTAA |
|  | Protein | MSGAGEGSGGGRAARLPRWTRQEILVLIEGKRVVEARGRGRGGRGAGGLAGAEPTKWAAVAEYCRRHGVERGPVQCRKRWSNLAGDYKKIREWERSLLLPSGGKEVSFWAMRNDARRERRLPGFFDREVYDIIEGRGGSRNAAAQAQVVVVEEEGKEKPAQALNSGRGVPEEGLFTSSSASSEDDDDEASVAPPAPVLPSPSPSPAPAHAVVAVTVPDKKNEAPVQDSSEKATSKGKQQEQIMDDPPPEQGGQKRQRSDDTTGEATDLQGQLIEILDQSSRMVAAQLEAQNINCQLDREQRKDQVSSLLSVLGKVADALYRIADKL |
| BdGT25 | CDS | ATGGCCTCCTGCGACGACGACTTCGGCCTCCTCGGTGACGACGCCCACCCCGCTCCCCAGCCTTCCGCCCAGCCGCCCCCGCCGCAGCAGGCCCAGACCTTCTGCTTCGTCGACGCCCCGGCCACGGGCTCCGGCTCCGGCCCCTTCGTGCCGGCCCAGGAGGAGGGCAACCACAGCTCCGAGCGCGGGAAGGCGTCGCACCACTCCAAGAGGCGGAGGGAGCGCGCGGAGGAGTTCAGCGATGGGGGCGAGTACTGTTCCTACATCAGCAGCAGCGCCGGGGGGAGGAAGGGCCGTGGCGGCGGCGGCGGCGGTTCCTCCGACTACCGGAAGGACCGGGAGGAGTGGACGGACGGCGCCATCAGCAGCCTCCTCGACGCATACACGGAGCGGTTCGAGCAGCTCAACCGCGGGAACCTGCGGGGGCGGGATTGGGAGGACGTCGCCGCTGCCGTGACCGACGGGCAGGGCAAAGGCGGCGCTGGCAAGAGCGTTGAGCAGTGTAAGAATAAGATCGACAACCTCAAGAAGCGCTACAAAGTAGAGTGCACACGCAACGGCGGCGCCGGTGCCGCCTCTGTCAGCCACTGGCCCTGGTACAGGCAGATGGAGCAAATCATCGGCAACTCCTCGTCGCCCGGCACCTCCAAGCCACTGGCGCCCACCAACGACGAGAAGCCGCGGCAACTGCTGCAGAACGCCAACAAAAGGTACCCTTCTTCCGGTACTGGCCCTCCTACCATGGTTCCCTGTTCCAGATTGACCCCTCTCTCAAATCCGAAATGGAAGAGGGTGCTCCTAAAGATTGGGGGCACTGCATTGGCTGGAGAAGCTCCTCACAATGTTGACCCAAAGGTAATCATGCTGATTGCCAGAGAAGTTCAAGTGGCGTGCCGCCATGGTGTTGAGGTGGCTATTGTCATGGGAGCTCGGAATATATTCTGTGGCGACAATTGGGTTGCTGCCACAGGCACTGATAGAGCTTCCACACACCCTGTTGGAATGATGGCAGCAGTGATGAATGCAGTATTGCTTCAAGCATCACTAGAAAAAATAGGTGTGGAGACACGAGTCCAGACTGCACTGATGATGCAAGAGGTAGCAGAACCATACATAAGAAGGCGAGCCATGCGCCATCTTCAAAAAGGAAGGGTAGTTATCTTTGGTGGAATTGGTGCTTGCATAGGAAATCCACTTTTCACAACAGATACCGCTGCTGCACTGAGAGCTTCTGAAATCAATGCAGATGTTGTCCTGAAAGGTATTGCTGGAGATGATGAATATGGTTGTCCTCCTAGAAGCAACAGTAATGCACTATTTGAGCACATCTCATTTAGGGAGTTAGCGGCAAGAGGAACAAGCAGCATGGATATGACAGCAATTACATGTTGTGAGGAGAACAATATTCCTGTTGTCATCTTTAACATGTTAGAGCCTGGTAACATCTCAAGAGCGATTTGTGGAGATCAAGTAGGTACTTTAGTTGACCAATCAGGAAGGATTACTTAA |
|  | Protein | MASCDDDFGLLGDDAHPAPQPSAQPPPPQQAQTFCFVDAPATGSGSGPFVPAQEEGNHSSERGKASHHSKRRRERAEEFSDGGEYCSYISSSAGGRKGRGGGGGGSSDYRKDREEWTDGAISSLLDAYTERFEQLNRGNLRGRDWEDVAAAVTDGQGKGGAGKSVEQCKNKIDNLKKRYKVECTRNGGAGAASVSHWPWYRQMEQIIGNSSSPGTSKPLAPTNDEKPRQLLQNANKRYPSSGTGPPTMVPCSRLTPLSNPKWKRVLLKIGGTALAGEAPHNVDPKVIMLIAREVQVACRHGVEVAIVMGARNIFCGDNWVAATGTDRASTHPVGMMAAVMNAVLLQASLEKIGVETRVQTALMMQEVAEPYIRRRAMRHLQKGRVVIFGGIGACIGNPLFTTDTAAALRASEINADVVLKGIAGDDEYGCPPRSNSNALFEHISFRELAARGTSSMDMTAITCCEENNIPVVIFNMLEPGNISRAICGDQVGTLVDQSGRIT |
| BdGT26 | CDS | ATGGACGACGACGACGTGCACGGGGACGTCTCCCCGTCGCCCACGCCCTCGCCGACCTCCTCCTCCCCGCTCCCGGTCGCCTCCGCGCTCCCCGTCGCCGACCCCGTCACGGTCGCCTCCGCCCCGCCCGGCGGCGGCCTCATGACGCTCGCGCTCCCGATCCAGAAGCACGGGGTCTATCCCAGCCACGGCGGCGGCGGCGGCGGCGGCGGTGGGAGGGAGGACGCGTGGAGCGAGGGCGCCACCTCCGCGCTCATCGACGCATGGGGGGAGCGCTTCGTCGCGCTCGGCCGCGGCAGCCTCCGCCACCCCCAGTGGCAGGAGGTCGCCGACTCCGTCTCCGCCCGCGAGAGCTACTCCAAGGCCGCCAAGTCCGACATCCAGTGCAAGAACCGCATCGACACGCTCAAGAAGAAGTACAAGATCGAGAGGGCCAAGCCGGTCTCGTCCTGGCAGTTCTACGACCGACTCGACGTGCTCCTTGCGCCCACCTACAACCAGAAGCCATCCGCCCATCCTAACGGAAGGAACCCGGTGCCCACAGCGCTGCGTGCCGGCTTTCCCAAGCGCAGCCGCACGCCCCTTATGCCCGCGGCGGCGCCTGTCAAGCAGAGGGCGCCGTCTCCGGAGCCGTCAGCGTCGTCCGAGTCTTCGGATGGGTTCCCGCCAGAGCCGGTGCTCCCGGTGGCGAACGGGAAGAGGAGGAGGACAGACGAAGGTCGTGGGGACGGCAGCAGCGGCAATGACCGCGTGCAGGGCCTGCGGGAGCTAGCGCAGGCGATACGGCGCTTCGGCGAGGCATATGAACGCATGGAGACAGCGAAGCTGGAGCAGGCTACGGAGATGGAACGCCGGCGCCTGGACTTTGCGCGTGAGCTGGAATCGCAGCGTGTACAGTTCTTCCTCAACACGCAGAGGGAGCTGTCGCAGGCCAAGAAGCACAACTCTCTGGCTGCCGCGGCTTCCCTGGCTGGTGCCACCATTGTTGGTGGTTCATCTAGGAGAATCGCATCGATTCCAGATCCCACCAGTAGGAGAAAGACATCGGTTCCTACTGTCAGGACGAGCAGCAATCACCATGGCCGCTACCACATTAGCGAGTCTAGCAGGCACCAACATCATCAGCATCGCCCTGCCCCACGGCCACATTATCAATACCATGACAATAATTTTGCTGCTTCTGCAGCTGCCAATGATGGTGAGCAGAGCGATGATGAAGAGGAGGAGGACGAGGGCGATGAAGAAGAGAGCCAGTGA |
|  | Protein | MDDDDVHGDVSPSPTPSPTSSSPLPVASALPVADPVTVASAPPGGGLMTLALPIQKHGVYPSHGGGGGGGGGREDAWSEGATSALIDAWGERFVALGRGSLRHPQWQEVADSVSARESYSKAAKSDIQCKNRIDTLKKKYKIERAKPVSSWQFYDRLDVLLAPTYNQKPSAHPNGRNPVPTALRAGFPKRSRTPLMPAAAPVKQRAPSPEPSASSESSDGFPPEPVLPVANGKRRRTDEGRGDGSSGNDRVQGLRELAQAIRRFGEAYERMETAKLEQATEMERRRLDFARELESQRVQFFLNTQRELSQAKKHNSLAAAASLAGATIVGGSSRRIASIPDPTSRRKTSVPTVRTSSNHHGRYHISESSRHQHHQHRPAPRPHYQYHDNNFAASAAANDGEQSDDEEEEDEGDEEESQ |
| BdGT27 | CDS | ATGCTCCTCTCGGGCCCGCAGCCCACCCCGCCGCTGCTGCTCCCGGAGAGCAGCGGCGAGGACGGCGGCGGCGGCGCCCACGACCTCGACTCCTCCTCGCGCGGCGGTGGCGCCGCCTCGGCCCCGAAGAAGCGCGCCGAGACGTGGGTCCAGGACGAGACGCTCAGCCTCATCGCGCTGCGCCGGGAGATGGACAACCACTTCAACACCTCCAAGTCCAACAAGCACCTCTGGGAGGCCATCTCCGCCAAGATGCGCGACCAGGGCTTCGACCGCTCCCCGACCATGTGCACGGACAAGTGGCGCAACCTCCTCAAGGAGTTCAAGAAGGCGCGCAGCCACGCCCGCAGCAGCGGCGCCGCCGGTGGGAACGGCGCCGCCAAGATGGCCTACTACAAGGAGATCGATGACCTGCTCAAGCGCCGCGGGAAGGCGGCCGGACCGGCCGCGGGGGCTGGCGGCGGAGGAGGCGCCCCGAAGAGCCCCACGCCCACCTCCAAGATCGAGTCCTTCCTCCAATTCGCGGATAAGGGTTTCGAAGATGCTAACATTCCATTTGGCCCTGTTGAAGCGAACGGTAGATCATTGCTGGGCGTTGATGATCGTCTGGAGCCTGACAGGCATCCCCTTGCCTTAACCACAGCTGATGCAGTTGTGGCCAGTGGTGTGAACCCATGGAATTGGAGAGACACTTCAACTAATGGTGGAGATAATCAAGCCACTTTTGGTGGGAGAGTTATTTTAGTCAAGTGGGGTGACTACACTAAAAGAATAGGCATTGATGGCACTGCTGAGGCGATTAAAGAGGCCATCAAAGCGGCATTTGGCTTAAGAACAAAACGAGCTTTTTGGCTTGAAGATGAAGATGAGGTTGTTCGTTCCTTGGACAGAGATATGCCCATCGGAACATATGCCCTGCATCTTGATGATGGGATACCAATCAAGCTCTGCACAGATGCTGACCGCATGACAGTCCGGACAGAAGATAAGACGTTCTACACTGAAGACGACTTCAGAGATTTCCTGTCGCGGCAAGGTTGGACACTCCTCAGGGAGTATAGTGGCTACAGAGTCGTTGATACTCTGGATGATCTTCGCCCTGGTGTGATTTATCAGGGGATGCGTTCGCTTATCGATTGA |
|  | Protein | MLLSGPQPTPPLLLPESSGEDGGGGAHDLDSSSRGGGAASAPKKRAETWVQDETLSLIALRREMDNHFNTSKSNKHLWEAISAKMRDQGFDRSPTMCTDKWRNLLKEFKKARSHARSSGAAGGNGAAKMAYYKEIDDLLKRRGKAAGPAAGAGGGGGAPKSPTPTSKIESFLQFADKGFEDANIPFGPVEANGRSLLGVDDRLEPDRHPLALTTADAVVASGVNPWNWRDTSTNGGDNQATFGGRVILVKWGDYTKRIGIDGTAEAIKEAIKAAFGLRTKRAFWLEDEDEVVRSLDRDMPIGTYALHLDDGIPIKLCTDADRMTVRTEDKTFYTEDDFRDFLSRQGWTLLREYSGYRVVDTLDDLRPGVIYQGMRSLID |
| BdGT28 | CDS | ATGCAGCAGCACCAGGGTGGCGGGTCCCAGTACGGGGCGCCGCCGCCGCAGGACATGGGGCCGTTCTCGACGGCGCCGCCGGCTCCCGGCCCGGTGCCGCTGAGCAGCCGGCCGCCCGCGCCGACGCAGCAGCAGCAGCCGACCTACGAGGAATTGGCGGCCGCGTCCGGCGCGGGCGCTGGCAGCGGCGGCTTCCCCGACGACGACATGCTCGGCGGCGACAACAGCGGCGGCGGTTTGGGCGCGAGCGGCGGCAACCGGTGGCCGCGGGAGGAGACGCTGGCTCTCATCAGGATCCGCTCCGAGATGGACGCTACCTTCCGCGACGCCACCCTCAAGGGTCCCCTCTGGGAAGAGGTCTCCAGGAAGCTTGCGGAGTTGGGCTACAAGAGGAACGCCAAGAAGTGCAAGGAGAAGTTCGAGAACGTGCACAAGTACTACAAGCGCACCAAGGAAGGCCGCACCGGCAGGCAGGACGGCAAGAGCTACCGCTTCTTCTCTGAGCTCGAGGCGCTGCATGCCACCACCACCGCGCAGCACCACCAGGAGCAGCAGCTGCCGCCGGTCAGCAGCGCCGCCGCCCCGCCGCAGCTGCAGCACGCCTTCGCGGCGCCCGCCGTATCCGCGCCGCTGCCGATGAGCACAATGCCGCCGCCACCGGGGCCGATCCAGCCGGCGCCCATATCTTCGGCGGCTCCGGCGGCACATGTAGCGGAGCTCCACCAGGCACCACCGCCCCTCGGCCTGCAAGGCTTGAGCTTCCCGTCCATGTCCGAGTCAGAGTCGGACGACGAGTCTGAGGATGACGAGATGACGGCGGAGACCGGCGGCGGCAGCGGCAGCCAGGATGGCCTCGGCAAGCGCAAGCACGGCGGCAGCAAGAAGCTGATGGCCTTCTTCGAGGGCCTCATGAAGCAGGTCATCCAGAGGCAAGAGGAGATGCAGCGGCGGTTCCTGGAGACGATGGAGAAGCGAGAGGCGGAGCGCATGGCAAGGGAGGAGGCGTGGCGCAAGCAGGAGGTGGCCCGCCTGAACCGCGAGCAGGAGATCCTCGCGCACGAGCGCGCCGCCGCCGCGTCCCGCGACGCGTCCATCATCGCCTTCCTCCAGCGCGTCGGCGCGGGGCAAGCCGTGCAAGTCCCCGCCCCCGTCGTCATCCCGATGCCCGCGCCGATGCAGGTCCAGACCCCAAGGCAACCTCCTCGACAGCAGCTCCCTCCTCCGCCGCCGCCCGCACAGGCCACGGCGCCACCGCCGCAGCCCATCCCAGCCGCGCCGCTCCAACAGCAACCGCCTCCACCACAGCCACAGCACAAGGAAACGACGATGGCGCGCCAAGAGGCCGTGACAACGCCGCGCAGCGCGCCGACACCGACACCGACACCGGCTTCCGGGGGCACATCACTGGCGCTGGTTCCCGTCTCTGAGCAGCAGGTTGAGAGTCATGGTCTCGGAGGAGGAGGGGATCATGGCGGGGCCGCGTCGTCGTCGCGGTGGCCCAAGACGGAGGTGCACGCGCTGATCCAGCTGCGGATGGACATGGACAACCGGTACCAGGAGAACGGGCCCAAGGGCCCGCTGTGGGAGGAGATCTCGTCGGGGATGCGGCGGCTGGGTTACAACAGGAACCCGAAGCGGTGCAAGGAGAAGTGGGAGAACATCAACAAGTACTTCAAGAAGGTAAAGGAGAGCAACAAGAGGCGGCCCGAGGACTCCAAGACTTGCCCCTACTTCCACCAGCTCGAGGCCATCTACCGCAAGAAGCACAACGGCGCCGGCGCCGCTGCCGCCGTGTCCGCCGCCAACGCCGCCGCCGTCGCGTCTGTCTGTGCTGTCACCACTGATCAGCAGCAGTCCCTGAGCCTGAACCGGCACGAGATCGAGGGCAAGAAGATCAACGACAACGACAAGAGGAACAACGGAGGAGTTGGAGGAGCCACACAGACGCAGGTGCCGACCAGTAACGGCGAGACAACGCCGACGACGACCCCGCCGCTCGACTTCAGTGAGAAAAAGCCAGAAGACACTGTGAGGGAGCTGAGCGAGCAGCAGCAGCACCGGGAGTTCACGACGGACGAGACCGACAGTGACGACATGGGCGATGACTACACTGACGGCGAGGACGGCGAGGACGATGGCAAAATGCAGTACAGGATACAGTTCCAGACGCCAAACCCCGTCGGCACTAACAATGCACCTCCACCGGCAACGACCCAGACGACCGCCGTTCCGCCGACTTCGACTCCATCGAGCTCCTTCCTCGCCATGGTTCAGTAG |
|  | Protein | MQQHQGGGSQYGAPPPQDMGPFSTAPPAPGPVPLSSRPPAPTQQQQPTYEELAAASGAGAGSGGFPDDDMLGGDNSGGGLGASGGNRWPREETLALIRIRSEMDATFRDATLKGPLWEEVSRKLAELGYKRNAKKCKEKFENVHKYYKRTKEGRTGRQDGKSYRFFSELEALHATTTAQHHQEQQLPPVSSAAAPPQLQHAFAAPAVSAPLPMSTMPPPPGPIQPAPISSAAPAAHVAELHQAPPPLGLQGLSFPSMSESESDDESEDDEMTAETGGGSGSQDGLGKRKHGGSKKLMAFFEGLMKQVIQRQEEMQRRFLETMEKREAERMAREEAWRKQEVARLNREQEILAHERAAAASRDASIIAFLQRVGAGQAVQVPAPVVIPMPAPMQVQTPRQPPRQQLPPPPPPAQATAPPPQPIPAAPLQQQPPPPQPQHKETTMARQEAVTTPRSAPTPTPTPASGGTSLALVPVSEQQVESHGLGGGGDHGGAASSSRWPKTEVHALIQLRMDMDNRYQENGPKGPLWEEISSGMRRLGYNRNPKRCKEKWENINKYFKKVKESNKRRPEDSKTCPYFHQLEAIYRKKHNGAGAAAAVSAANAAAVASVCAVTTDQQQSLSLNRHEIEGKKINDNDKRNNGGVGGATQTQVPTSNGETTPTTTPPLDFSEKKPEDTVRELSEQQQHREFTTDETDSDDMGDDYTDGEDGEDDGKMQYRIQFQTPNPVGTNNAPPPATTQTTAVPPTSTPSSSFLAMVQ |
| BdGT29 | CDS | ATGACGACTTCCCCTCCTTCTCCCACAACCAACGCGATGATGCCCTCCGCCCGCCGCGTGCCGCCCCCGTGCTGGACCCCCGACGAGACCCTCGCGCTCGCCCGCGCCTACACCGCGCGCCGCCTCGCCGTCGGCCGCGCCCACCTCGCCTCCGCCGACTGGGCCGCCGTCGCCGCCGAGGCCACCCCCTCCAAGACCGCCAGGCAGTGCCGCCACAAGATCGAGAAGCTCCGCCGCCGCCTCCGCTCCAAGCGCCGCCGCCCCTTCCCGCTCCTCGACGGCATAGATCTGCTCGACGACCCTTCCCCGTCGCCCTCCCACTCCCGCTCCCAATCCCCGTCCCCGCCGCCACCGGCTTCCCCGCCTCCTCCCTCCGCCTCCCCGCCTAAGAAGAGGAAACGGCCCGATGCCGCGGAGAGCGACGTGGTCAAGGCGCTGAAGGCGATCGGAGAGGGGTTCGTGCGCGCGGAGCTGAGGAGGATGGAGGCGGCGAGAGAGACGCAGCGGATGCGCATGGAGATGGCGCTGCGACACCTCGACGCCCAGCGGCAGCTCGTGGACGGCCTTGTTGGCCGCATCGTCGACGCGCTGGAATGA |
|  | Protein | MTTSPPSPTTNAMMPSARRVPPPCWTPDETLALARAYTARRLAVGRAHLASADWAAVAAEATPSKTARQCRHKIEKLRRRLRSKRRRPFPLLDGIDLLDDPSPSPSHSRSQSPSPPPPASPPPPSASPPKKRKRPDAAESDVVKALKAIGEGFVRAELRRMEAARETQRMRMEMALRHLDAQRQLVDGLVGRIVDALE |
| BdGT30 | CDS | ATGGAGGCGGGCGTAGGAGGAGGAGGGTTTAGGGACGAGAGGGTGCCGCCGTGGGGCACGCAGGAGACGCGCGAGCTGATCGCGGCGCGCGGGGAGCTGGAGCGGGAGGCCGCTGCTGCTTCCGCCGGCGCCGCCGCGAGCCGTAGCGCCAAGACGCTCTGGGAGGCGGTGGCGGCCAGGCTCCGGGCGCGCGGGTACCGCCGCACCGCCGACCAGTGCAAGTGCAAGTGGAAGAACCTCGTCAACCGCTACAAGGGGAAAGAAACATCTAATCCGGAAAATGGTAGACAGTGTCCCTTCTTTGAAGAATTACATGCAGTCTTCACTGAACGTGCTAGAAATATGCAACGGGAACTCCTTCAGTCTGAATCAGGGGCTTCAGTTAAAATGAAACTAAAGCGAACCAGTGATGACCGGTCATCCGGGGAGTCTGATGATGAGGAAGATGAAGTTGAAGTAAGCGAGGATGAGAAACCCATGCGAAGCAGAAAGCGGAAGGCTGGTGATAAGGGGCAGCAATTACGACGAACAGTGGAAAATTCAAATACGGGCAGGTCAAGCATCCGTGACCTGTTGCATGATTTCCTGGTTCAACAACAACACATGGATATCCAGTGGCGTGAGGTGATGGAGCGACGTGCCCAGGAACGGCTCGTTTTCGAACAAGAATGGAGGCGGTCGATGCAGAAGCTGGAGCAGGAGAGGCTGATGTTGGAGCACTCATGGATGGAACGAGAGGAACAGCGGAGGATGAGAGAAGAAGCAAGAGCTCAGAAAAGGGATTCCCTCCTGACCACCCTACTGAACAAACTCTTACAAGATCTATAG |
|  | Protein | MEAGVGGGGFRDERVPPWGTQETRELIAARGELEREAAAASAGAAASRSAKTLWEAVAARLRARGYRRTADQCKCKWKNLVNRYKGKETSNPENGRQCPFFEELHAVFTERARNMQRELLQSESGASVKMKLKRTSDDRSSGESDDEEDEVEVSEDEKPMRSRKRKAGDKGQQLRRTVENSNTGRSSIRDLLHDFLVQQQHMDIQWREVMERRAQERLVFEQEWRRSMQKLEQERLMLEHSWMEREEQRRMREEARAQKRDSLLTTLLNKLLQDL |
| BdGT31 | CDS | ATGTCGGCGTCGCCGGACCCGTCGCCGTCTTCCTCCACGCCGGCGGGCCACCTCCGCACTCACCCCTACCCACACCACCACAACCTCCACCTCTCAACTCCTCCTCCGCCGCAGCCCCACCCGCCGGCGGCGCCGGGCCCGCTCGCCGTGACGCCGCCGCACCAGCAAGCTCTGGCCCCGTACTCCGCCCCACCCCCTTCCCCTGCGTCGACCCCTCGAGACTACCGGAAGGGCAACTGGACGCTCCACGAGACGCTCGTGCTCATCACCGCCAAGCGCCTCGACGACGACCGCCGCGCCGGTGGCGGCGTCGCCGGGTCCTCGTCTTCGTCTCCGGCCGGGCCGAGTGCGCTGCCGAGGTCGGCGGAGCAGCGGTGGAAGTGGGTGGAGAACTACTGCTGGTCCCAGGGGTGCCTGCGCAGCCAGAACCAGTGCAACGACAAGTGGGACAACCTGCTCAGGGACTACAAGAAGGTCCGCGACTTCCACAACCGCCTCGCTTCCAACTCCACCGCCCCTTCCGGCTCCGGCGCCGCTCCGGCTGCGGCGGCGCCATTGCCGACCTCGTACTGGGCCATGGAGAGGCACGAGAGGAAGGAGCGTAACCTGCCGACCAACCTGGCGGCCGAGGTCTACGACGCGCTCTGCGACGTGCTCTCCCGCCGCGCCGCCCGCAGGGGCGGCGCCCCCGTCGCGCCCTCGCCGCCCCCGCCGCTCGCGCTCCCGGCGCCACCGCTTCATCCGCCGCCGAAGCCGCTCCTCTCGCAGCAGCAGCAGCGGATTCCTCCTCACCACCCCCCGCTGCTGCAGCTCCCGCCGCCGCCCGTTGCGGTGGCGCCGCCGCCGACGACGACGTCCGTTTCCGAGGATGAGATGACGGGGTCGTCGTCGGACTCCGGCTCCTCGTCGGGGCGAGCTGGCGGCGGCGGGGACGAGCCGGAGGCGAAGCGGCAGCGGCGGCAACGAGGGCGGGTGAGCGACAGGGGACGGGTGGGATCGAGCGTTGTGCGGGGCGCGACGGTGCTGGCTCGCACGCTGGTGGCGTGCGAGGAGAAGCGGGAGCGGCGGCACCGGGAGCGGCTGGAGCTGGAGGAGCGGCGGCTGCGGCTGGAGGCGGAGCGCGCCGAGGCCCGGCGCCAGGGCTTCGCGGGCCTCATCTCCGCCGTCCACTCCCTCTCCGGCGCCATCCACGCCCTCGTCTCCGACCACGCCCGCAGCGGCGACTCCTCCAGGTGA |
|  | Protein | MSASPDPSPSSSTPAGHLRTHPYPHHHNLHLSTPPPPQPHPPAAPGPLAVTPPHQQALAPYSAPPPSPASTPRDYRKGNWTLHETLVLITAKRLDDDRRAGGGVAGSSSSSPAGPSALPRSAEQRWKWVENYCWSQGCLRSQNQCNDKWDNLLRDYKKVRDFHNRLASNSTAPSGSGAAPAAAAPLPTSYWAMERHERKERNLPTNLAAEVYDALCDVLSRRAARRGGAPVAPSPPPPLALPAPPLHPPPKPLLSQQQQRIPPHHPPLLQLPPPPVAVAPPPTTTSVSEDEMTGSSSDSGSSSGRAGGGGDEPEAKRQRRQRGRVSDRGRVGSSVVRGATVLARTLVACEEKRERRHRERLELEERRLRLEAERAEARRQGFAGLISAVHSLSGAIHALVSDHARSGDSSR |
