# Supplementary material for: Identification and in Silico Characterization of GT Factors Involved in Phytohormone and Abiotic Stresses Responses in Brachypodium distachyon
Source: Int J Mol Sci. 2019 Aug 23;20(17):4115. doi: 10.3390/ijms20174115 (PMC6747514; doi:10.3390/ijms20174115)
Supplement: Supplementary file 1 [file ijms-20-04115-s001.zip › supplementary file/Table S2.doc]

Table S2 The list of qRT-PCR primers of *BdGT* genes.

| **Gene Name** | **Forward Primer** | **Reverse Primer** |
| --- | --- | --- |
| **BdGT1** | CGTTTTGTTCTTGGGGGCG | TATTCCTGCCGATGCCGATG |
| **BdGT2** | CTCGGTTCCCCACGGACT | ACCCCTCCGTTTTCGTGATG |
| **BdGT3** | AAGACCTGCCCCTACTTCCA | CGTTGCTGTTGCCATTACCA |
| **BdGT4** | CTCCATGCTCTGGCTCCTTC | TTGCTGTTGAGGCTGTGGAA |
| **BdGT5** | GGGATAGTGGTGTCAGCGAG | CTTCACCCTTGCGAGCTTCA |
| **BdGT6** | AAAAAGGCCAAGGGGAAGCA | TGTTGATTTCCTCCTCCGCT |
| **BdGT7** | TCTGTTTCGTCTCGCTCTGG | CGCTTCAGCTCCAGATCCTT |
| **BdGT8** | CGTTATTCGTGGGGTGTGGA | TGTGCTGCCTCTTGCTACTC |
| **BdGT9** | CCTCCTCCCAGCACGAATG | GCCTCCGTCACCTGTCTC |
| **BdGT10** | CATAGCACAAGCAGCAGCAG | GGCTTGTAGATGGCCTCCAG |
| **BdGT11** | GAACGAGCCGAAAAAGAGCC | TTCAGGTAGCTCACAAGCCC |
| **BdGT12** | AGCCGGTAAAGCCGGTAAAG | CGTCCTCCTCCGTTTCTTCC |
| **BdGT13** | GACCTACCGCTTCTTCCAGC | ATCTTCCTCGTCCTCGTCCT |
| **BdGT14** | CTTCTTCCCGCTCCTCCAC | TTGGGGATCGTAAACCGCAG |
| **BdGT15** | ACAGGGTGAAGGCAGAGGA | GCGGCTATGGTGTCCTGAAT |
| **BdGT16** | GGTTGTCGCCTTGCCGAT | GGATCTCCAGTAGCTTGCCC |
| **BdGT17** | GGGAAATCGGCATGAACTGC | GATAACCCAAGGCAACGCAC |
| **BdGT18** | CTGGCGGTCTATCTGCTGAG | CCAACCGCTCATTCTCCAGT |
| **BdGT19** | CGCTGTTATGATTCCGCGTT | GACGCTGCTATCTGCTCCAT |
| **BdGT20** | AGTCCGATGACGAGGAGGAA | CCATTCGCACTTTATGCGGG |
| **BdGT21** | CTGAACTGCGGGAAGAGGAG | GCAAGCAATACCGCAACCAA |
| **BdGT22** | CGACAGAGAGAGCCGAGAAG | AGCCAGCAACTCAATGCTCT |
| **BdGT23** | TAGGAGGTGGGAGGAAGAGC | GCTGGATTTGCCACTACCCT |
| **BdGT24** | TGGAAGAGGAGGGGAAGGAG | CCTCGTTCTTCTTGTCGGGT |
| **BdGT25** | ATCATCGGCAACTCCTCGTC | AGCACCCTCTTCCATTTCGG |
| **BdGT26** | CGAGTCTTCGGATGGGTTCC | CGTTCCATCTCCGTAGCCTG |
| **BdGT27** | CCATTTGGCCCTGTTGAAGC | CTCTCCCACCAAAAGTGGCT |
| **BdGT28** | GAGGATGACGAGATGACGGC | CCTCTCGCTTCTCCATCGTC |
| **BdGT29** | AGAGGAAACGGCCCGATG | GACGATGCGGCCAACAAG |
| **BdGT30** | ATACGGGCAGGTCAAGCATC | CATCCTCCGCTGTTCCTCTC |
| **BdGT31** | GGGTGGAGAACTACTGCTGG | TTCCTCTCGTGCCTCTCCAT |
