# Supplementary material for: Identification and in Silico Characterization of GT Factors Involved in Phytohormone and Abiotic Stresses Responses in Brachypodium distachyon
Source: Int J Mol Sci. 2019 Aug 23;20(17):4115. doi: 10.3390/ijms20174115 (PMC6747514; doi:10.3390/ijms20174115)
Supplement: Supplementary file 1 [file ijms-20-04115-s001.zip › supplementary file/Table S3.doc]

Table S3A Expression data of *BdGT* genes after phytohormone treatment.

|  | **6-BA** | | |  | **ABA** | | |  | **GA** | | |  | **NAA** | | |
| --- | --- | --- | --- | --- | --- | --- | --- | --- | --- | --- | --- | --- | --- | --- | --- |
|  | fold-change | p-value | up/down |  | fold-change | p-value | up/down |  | fold-change | p-value | up/down |  | fold-change | p-value | up/down |
| **BdGT1** | -8.04 | 0.00 | down |  | -4.63 | 0.07 |  |  | -0.49 | 0.37 |  |  | -8.33 | 0.00 | down |
| **BdGT2** | 5.29 | 0.02 | up |  | 7.26 | 0.00 | up |  | 10.46 | 0.00 | up |  | -0.56 | 0.75 |  |
| **BdGT3** | 7.50 | 0.02 | up |  | -8.89 | 0.00 | down |  | -0.77 | 0.30 |  |  | -3.89 | 0.02 | down |
| **BdGT4** | -0.68 | 0.03 |  |  | 1.68 | 0.00 | up |  | 2.53 | 0.00 | up |  | -1.41 | 0.00 | down |
| **BdGT5** | -2.59 | 0.01 | down |  | -0.96 | 0.09 |  |  | -1.47 | 0.09 |  |  | 0.61 | 0.03 |  |
| **BdGT6** | -7.64 | 0.02 | down |  | 0.03 | 0.90 |  |  | -4.87 | 0.04 | down |  | -6.01 | 0.02 | down |
| **BdGT7** | -2.81 | 0.00 | down |  | 0.66 | 0.25 |  |  | -2.19 | 0.22 |  |  | 2.27 | 0.00 | up |
| **BdGT8** | -2.56 | 0.01 | down |  | -1.12 | 0.02 | down |  | -9.62 | 0.00 | down |  | 0.47 | 0.17 |  |
| **BdGT9** | -5.00 | 0.01 | down |  | 0.29 | 0.57 |  |  | 2.13 | 0.05 | up |  | 1.44 | 0.02 | up |
| **BdGT10** | -0.34 | 0.15 |  |  | -1.67 | 0.00 | down |  | -1.07 | 0.01 | down |  | -7.72 | 0.01 | down |
| **BdGT11** | 0.90 | 0.09 |  |  | -0.12 | 0.87 |  |  | 0.89 | 0.17 |  |  | -3.85 | 0.04 | down |
| **BdGT12** | -9.26 | 0.00 | down |  | -2.03 | 0.03 | down |  | -3.90 | 0.00 | down |  | -8.87 | 0.00 | down |
| **BdGT13** | 1.90 | 0.02 | up |  | 3.41 | 0.00 | up |  | -1.45 | 0.01 | down |  | -1.14 | 0.18 |  |
| **BdGT14** | -2.22 | 0.01 | down |  | -0.26 | 0.66 |  |  | -1.35 | 0.25 |  |  | -4.96 | 0.00 | down |
| **BdGT15** | -2.47 | 0.01 | down |  | -0.99 | 0.31 |  |  | -8.42 | 0.00 | down |  | -6.23 | 0.00 | down |
| **BdGT16** | -0.86 | 0.06 |  |  | 0.06 | 0.91 |  |  | 2.15 | 0.00 | up |  | 1.18 | 0.02 | up |
| **BdGT17** | -1.73 | 0.10 |  |  | -1.30 | 0.02 | down |  | -6.50 | 0.00 | down |  | -2.30 | 0.02 | down |
| **BdGT18** | 0.60 | 0.44 |  |  | -1.13 | 0.20 |  |  | -0.52 | 0.51 |  |  | -4.92 | 0.00 | down |
| **BdGT19** | 8.38 | 0.00 | up |  | -4.47 | 0.26 |  |  | -0.31 | 0.64 |  |  | -4.05 | 0.03 | down |
| **BdGT20** | 3.94 | 0.00 | up |  | -8.88 | 0.00 | down |  | -7.47 | 0.00 | down |  | -9.94 | 0.00 | down |
| **BdGT21** | 1.02 | 0.05 | up |  | 1.32 | 0.03 | up |  | 1.59 | 0.03 | up |  | 2.20 | 0.01 | up |
| **BdGT22** | -1.06 | 0.06 |  |  | -1.57 | 0.04 | down |  | -0.42 | 0.28 |  |  | 1.60 | 0.03 | up |
| **BdGT23** | -0.89 | 0.22 |  |  | -0.35 | 0.36 |  |  | 0.48 | 0.19 |  |  | 3.05 | 0.00 | up |
| **BdGT24** | 1.28 | 0.02 | up |  | 3.13 | 0.00 | up |  | 2.80 | 0.00 | up |  | -0.16 | 0.70 |  |
| **BdGT25** | -1.02 | 0.33 |  |  | 1.75 | 0.01 | up |  | 2.31 | 0.01 | up |  | -0.74 | 0.35 |  |
| **BdGT26** | -1.70 | 0.05 | down |  | -0.68 | 0.35 |  |  | -4.16 | 0.04 | down |  | -2.50 | 0.02 | down |
| **BdGT27** | 0.55 | 0.27 |  |  | 2.35 | 0.01 | up |  | 2.87 | 0.01 | up |  | 1.20 | 0.02 | up |
| **BdGT28** | -4.99 | 0.01 | down |  | -1.01 | 0.05 | down |  | -1.58 | 0.01 | down |  | -7.07 | 0.00 | down |
| **BdGT29** | -6.94 | 0.00 | down |  | -3.63 | 0.01 | down |  | -0.54 | 0.42 |  |  | -4.62 | 0.00 | down |
| **BdGT30** | 3.67 | 0.00 | up |  | 1.27 | 0.01 | up |  | 1.70 | 0.03 | up |  | 3.85 | 0.00 | up |
| **BdGT31** | 0.83 | 0.04 |  |  | 2.63 | 0.00 | up |  | 1.79 | 0.00 | up |  | -4.65 | 0.02 | down |

Table S3B Expression data of *BdGT* genes after abiotic stresses.

|  | Cold | | |  | Heat | | |  | H_2_O_2_ | | |  | Salt | | |  | PEG | | |
| --- | --- | --- | --- | --- | --- | --- | --- | --- | --- | --- | --- | --- | --- | --- | --- | --- | --- | --- | --- |
|  | fold-change | p-value | up/down |  | fold-change | p-value | up/down |  | fold-change | p-value | up/down |  | fold-change | p-value | up/down |  | fold-change | p-value | up/down |
| **BdGT1** | -5.33 | 0.02 | down |  | 2.93 | 0.01 | up |  | -5.45 | 0.00 | down |  | -8.51 | 0.00 | down |  | -6.86 | 0.00 | down |
| **BdGT2** | -2.44 | 0.00 | down |  | 5.16 | 0.00 | up |  | -2.69 | 0.01 | down |  | -1.46 | 0.18 |  |  | 5.36 | 0.00 | up |
| **BdGT3** | -9.60 | 0.00 | down |  | -6.28 | 0.00 | down |  | -2.76 | 0.02 | down |  | -4.63 | 0.00 | down |  | 1.74 | 0.00 | up |
| **BdGT4** | -1.42 | 0.06 |  |  | 2.07 | 0.00 | up |  | 0.42 | 0.15 |  |  | -0.83 | 0.01 |  |  | -1.58 | 0.02 | down |
| **BdGT5** | -1.39 | 0.06 |  |  | -4.09 | 0.04 | down |  | -1.42 | 0.04 | down |  | 1.18 | 0.01 | up |  | -2.28 | 0.00 | down |
| **BdGT6** | -4.09 | 0.05 | down |  | -5.66 | 0.06 |  |  | -0.97 | 0.01 |  |  | -5.42 | 0.01 | down |  | -9.14 | 0.01 | down |
| **BdGT7** | 2.23 | 0.02 | up |  | -0.94 | 0.25 |  |  | -0.71 | 0.15 |  |  | 2.13 | 0.01 | up |  | 3.37 | 0.00 | up |
| **BdGT8** | -0.53 | 0.21 |  |  | -8.31 | 0.00 | down |  | -1.48 | 0.01 | down |  | -0.28 | 0.51 |  |  | 0.65 | 0.07 |  |
| **BdGT9** | -2.22 | 0.00 | down |  | 5.01 | 0.00 | up |  | -0.15 | 0.80 |  |  | -0.32 | 0.87 |  |  | 4.25 | 0.00 | up |
| **BdGT10** | -6.35 | 0.00 | down |  | -3.15 | 0.02 | down |  | -2.66 | 0.00 | down |  | -3.71 | 0.00 | down |  | -5.07 | 0.00 | down |
| **BdGT11** | 1.44 | 0.03 | up |  | -2.62 | 0.11 |  |  | 2.14 | 0.01 | up |  | 3.24 | 0.00 | up |  | -0.35 | 0.52 |  |
| **BdGT12** | -8.78 | 0.00 | down |  | -6.01 | 0.00 | down |  | -10.41 | 0.00 | down |  | -6.03 | 0.00 | down |  | -9.85 | 0.00 | down |
| **BdGT13** | 3.44 | 0.00 | up |  | 2.59 | 0.02 | up |  | 2.68 | 0.01 | up |  | -4.88 | 0.01 | down |  | -2.95 | 0.00 | down |
| **BdGT14** | -1.88 | 0.06 |  |  | 3.11 | 0.00 | up |  | -0.66 | 0.20 |  |  | -3.38 | 0.00 | down |  | -0.82 | 0.07 |  |
| **BdGT15** | -1.80 | 0.02 | down |  | -3.99 | 0.01 | down |  | -10.35 | 0.00 | down |  | -7.47 | 0.00 | down |  | -1.84 | 0.02 | down |
| **BdGT16** | 1.13 | 0.04 | up |  | -1.05 | 0.04 | down |  | -4.11 | 0.00 | down |  | -2.44 | 0.03 | down |  | 0.38 | 0.34 |  |
| **BdGT17** | -6.22 | 0.01 | down |  | -5.93 | 0.00 | down |  | -1.39 | 0.01 | down |  | -2.33 | 0.01 | down |  | -4.08 | 0.00 | down |
| **BdGT18** | 0.32 | 0.67 |  |  | -2.93 | 0.03 | down |  | -0.87 | 0.31 |  |  | 0.92 | 0.28 |  |  | -1.81 | 0.08 |  |
| **BdGT19** | -5.50 | 0.06 |  |  | -6.26 | 0.00 | down |  | -2.56 | 0.09 |  |  | -2.48 | 0.03 | down |  | 2.31 | 0.03 | up |
| **BdGT20** | -9.66 | 0.00 | down |  | -0.86 | 0.03 |  |  | -9.39 | 0.00 | down |  | -1.54 | 0.00 | down |  | -2.20 | 0.02 | down |
| **BdGT21** | -0.56 | 0.18 |  |  | -0.50 | 0.25 |  |  | 1.64 | 0.02 | up |  | 1.51 | 0.02 | up |  | -0.66 | 0.14 |  |
| **BdGT22** | 0.70 | 0.11 |  |  | -1.43 | 0.03 | down |  | -1.84 | 0.01 | down |  | 0.70 | 0.13 |  |  | 0.00 | 0.99 |  |
| **BdGT23** | 1.36 | 0.02 | up |  | 2.03 | 0.00 | up |  | 1.11 | 0.15 |  |  | 0.05 | 0.92 |  |  | 1.08 | 0.01 | up |
| **BdGT24** | 0.37 | 0.27 |  |  | 4.86 | 0.00 | up |  | 1.77 | 0.01 | up |  | -0.92 | 0.15 |  |  | -0.48 | 0.22 |  |
| **BdGT25** | -1.24 | 0.06 |  |  | -2.76 | 0.01 | down |  | 1.57 | 0.02 | up |  | 3.77 | 0.00 | up |  | 1.42 | 0.09 |  |
| **BdGT26** | 1.07 | 0.18 |  |  | 0.16 | 0.77 |  |  | -2.05 | 0.05 | down |  | -5.23 | 0.04 | down |  | -3.11 | 0.02 | down |
| **BdGT27** | 3.61 | 0.00 | up |  | 4.81 | 0.00 | up |  | 2.80 | 0.00 | up |  | 2.10 | 0.00 | up |  | -0.18 | 0.59 |  |
| **BdGT28** | -8.15 | 0.00 | down |  | -0.84 | 0.07 |  |  | -1.23 | 0.16 |  |  | -5.87 | 0.03 | down |  | -11.16 | 0.00 | down |
| **BdGT29** | -7.36 | 0.00 | down |  | -2.83 | 0.02 | down |  | -2.84 | 0.04 | down |  | -3.23 | 0.02 | down |  | -7.26 | 0.00 | down |
| **BdGT30** | 3.31 | 0.00 | up |  | -0.72 | 0.59 |  |  | 0.38 | 0.52 |  |  | 1.46 | 0.01 | up |  | 1.56 | 0.01 | up |
| **BdGT31** | -2.27 | 0.00 | down |  | 4.88 | 0.00 | up |  | 1.47 | 0.00 | up |  | -2.33 | 0.00 | down |  | -3.19 | 0.01 | down |
